# Patient perspectives on how to demonstrate respect: Implications for clinicians and healthcare organizations

**DOI:** 10.1371/journal.pone.0250999

**Published:** 2021-04-29

**Authors:** Celina Bridges, Devan M. Duenas, Hannah Lewis, Katherine Anderson, Douglas J. Opel, Benjamin S. Wilfond, Stephanie A. Kraft

**Affiliations:** 1 University of Washington School of Medicine, Seattle, Washington, United States of America; 2 Treuman Katz Center for Pediatric Bioethics, Seattle Children’s Research Institute, Seattle, Washington, United States of America; 3 Denver Health Ambulatory Care Services, Denver, Colorado, United States of America; 4 Division of Bioethics and Palliative Care, Department of Pediatrics, University of Washington School of Medicine, Seattle, Washington, United States of America; Universidade Estadual Paulista Julio de Mesquita Filho Faculdade de Medicina Campus de Botucatu, BRAZIL

## Abstract

**Objective:**

Clinicians and healthcare organizations are ethically obligated to treat patients with respect, yet it is not clear what actions best demonstrate respect to patients. This exploratory qualitative study aimed to understand what actions on both an individual and organizational level effectively demonstrate respect for primary care patients.

**Methods:**

We conducted semi-structured telephone interviews with primary care patients in an integrated healthcare delivery system in Oregon and an integrated safety net health system in Colorado who were participating in a genomics implementation research study of a hereditary cancer screening program. We systematically coded interview transcripts using a coding framework developed based on iterative review of the interview guide and transcripts. We further analyzed the data coded with sub-codes relating to patients’ experiences with respect in healthcare using a descriptive content analysis approach.

**Results:**

We interviewed 40 English-speaking (n = 30, 75%) and Spanish-speaking (n = 10, 25%) patients. Most interviewees identified as female (n = 35, 88%) and either Hispanic/Latino(a) (n = 17, 43%) or White or European American (n = 15, 38%). Interviewees identified two categories of efforts by individual clinicians that demonstrate respect: engaging with patients and being transparent. They identified five efforts by healthcare organizations: promoting safety and inclusivity, protecting patient privacy, communicating about scheduling, navigating financial barriers to care, and ensuring continuity of care.

**Conclusions:**

Our findings suggest that patients’ experiences of respect depend on efforts by individual clinicians as well as healthcare organizations. Our findings offer insight into how clinicians can build stronger partnerships with patients and how organizations can seek to promote access to care and patient safety and comfort. They also illustrate areas for future research and quality improvement to more effectively respect patients.

## Introduction

All patients deserve to be treated with respect [1]. In clinical care, respect is foundational to the formation of genuine relationships, strengthening clinicians’ moral commitment to their patients and encouraging authentic interactions [2]. Effectively conveying respect may also have positive effects on equitable health outcomes, patient satisfaction, and mutual trust [3–7]. Because clinical care takes place within an organizational context, healthcare organizations as well as individual clinicians must consider their roles in ensuring patients feel respected.

Clinicians and bioethicists have often defined “respect for persons” with a focus on supporting individual autonomy [8], but treating patients with respect entails broader responsibilities. Beach and colleagues define respect as the “recognition of the unconditional value of patients as persons” [9], which has been expanded upon to include attention to needs [10,11] and “actions that honor or acknowledge a person’s dignity” [12]. While these definitions, which draw on work in cardiac care [11], critical care [13,14], and community settings [15], describe generally what respect may mean, respect is perceived uniquely by each patient and may differ across cultures and communities [16], so understanding specific actions and behaviors that demonstrate respect for those perceiving it within a particular context is essential.

While some studies have examined interpersonal aspects of respect, and recent work has highlighted the need for healthcare organizations to be accountable for systems-level factors that contribute to patients’ experiences of disrespect [17,18], there has been comparatively less attention paid to how healthcare organizations contribute, in combination with individual clinicians, to patients’ experiences of respect. Interpersonal interactions are informed, directly and indirectly, by systemic factors that in some cases stem from, or can be mitigated by, organizational policies and practices. Further, we hypothesize that patients’ experiences of respect reflect not only interpersonal interactions with individual clinicians, but also their overall experience interacting with a healthcare organization, much of which depends on choices made at an organizational level. Exploring these dynamics is particularly important in settings such as primary care where there may be an opportunity to build respectful relationships over time across repeated interactions with one or more clinicians within an organizational setting. To fill this gap and examine how patients perceive the role of their clinicians and healthcare institutions in demonstrating respect, we conducted this qualitative study of primary care patients’ perspectives on respect in healthcare.

## Methods

We interviewed participants in a study implementing clinical exome sequencing for hereditary cancer syndromes in diverse primary care settings. Interviews addressed patients’ experiences with and opinions on respect and trust in healthcare and research; this manuscript examines responses related to respect in healthcare. Other results are reported elsewhere [19]. We analyzed our data using a descriptive qualitative approach, which allowed us to understand the experience and meaning of respect in depth in patients’ own words [20,21].

### Participants

Interviewees were identified through their participation in the Cancer Health Assessments Reaching Many (CHARM) study, which evaluated the implementation of a screening and genetic testing program for hereditary cancer risk in diverse primary care settings. CHARM participants were English- or Spanish-speaking patients, age 18–49 years, at Kaiser Permanente Northwest, an integrated healthcare delivery system in Oregon, and Denver Health, an integrated safety net health system in Colorado, who screened at high risk of hereditary breast and ovarian cancer syndrome and/or Lynch syndrome based on family history or insufficient knowledge of family history. The CHARM study is further described elsewhere [22].

CHARM participants were eligible to be interviewed if they had enrolled but not yet received genetic test results or received negative results within the past month. We used purposive sampling based on race and ethnicity, socioeconomic status, and language to include individuals with diverse backgrounds. We recruited eligible individuals by email or postal mail and followed up by phone. This study was approved by the Kaiser Permanente Northwest IRB as part of the CHARM study (Study 733).

### Interviews

We developed and pilot-tested a semi-structured, individual interview guide including questions about respect and trust in healthcare and research. Questions about experiences with respect in healthcare served the dual role of introducing the concept of respect and collecting data. Our questions about this topic were broad, as our goal was to understand the range of experiences that interviewees reported as demonstrating respect or a lack thereof. We assumed that participants would have personal experiences that they would classify as respectful or not within the medical setting, stemming from both individual and institutional actions. We included prompts about individual and institutional actors to explore respect at each level. We also included a prompt asking about any experiences feeling a lack of respect from a response to the interviewee’s personal characteristics or appearance, as prior work has emphasized being recognized as a valued individual as a central component of respect and we wanted to explicitly open space for interviewees to address any experiences of a lack of respect resulting from discrimination if they felt comfortable doing so (Table 1), Four experienced interviewers (DMD, SAK, ARH, DR) were trained on the interview guide, conducted all interviews by phone, and took field notes during or immediately after each interview. Interviewees consented to be contacted for interviews at the time they consented to the CHARM study, and they provided verbal consent after hearing information about the interview study at the start of each interview, which was documented by the interviewer. Interviews lasted approximately 30–45 minutes each. Interviews were audio recorded, professionally transcribed and (for Spanish interviews) translated into English by certified translators, reviewed for accuracy and removal of potentially identifying information by the interviewer, and uploaded to the cloud-based qualitative analysis program Dedoose (dedoose.com) for data management and analysis.

**Table 1:** Interview questions about respect in healthcare.

Topic	Question
**Respect in medical care**	To get started, I’d like to ask you to think about your experiences with medical care, including the doctors you see and the clinics you visit. Could you give me some examples of the sorts of things that make you feel respected in your medical care? Probes if needed: • What is it about those things that make you feel respected? • Please tell me about a time when you felt respect from your medical care. • What about individual medical providers or staff? How could they show respect? • What about the policies that a medical institution has in place? What kinds of policies would show respect?
**Lack of respect in medical care**	Could you give me some examples of the sorts of things that make you feel a lack of respect from your medical care? Probes if needed: • What is it about those things that make you feel a lack of respect? • Please tell me about a time when you felt a lack of respect from your medical care. • Have you ever felt a lack of respect in the medical setting based on who you are, how you look, or what you believe? Would you mind telling me about that/those experience(s)? • Could you imagine anything that might make you feel a lack of respect? What about your friends or family? • What about individual medical providers or staff? How could they show a lack of respect? • What about the policies that a medical institution has in place? What kinds of policies would show a lack of respect?

### Analysis

We used a descriptive content analysis approach to analyze our data, drawing on interviewees’ own words to identify our analytic domains wherever possible [20,21]. We developed a coding framework beginning with our interview guide then expanding on it using iterative open coding and review of interview transcripts to inductively identify the range of ways in which patients described their experiences and perspectives. Two coders (DMD, HL) systematically coded all transcripts, of which 25% were coded to consensus and reviewed by a third co-author as tie-breaker (BSW or SAK) to ensure consistency. We further modified the codebook as we coded, applying new codes retroactively as needed, ultimately identifying 13 sub-codes that described patients’ experiences with respect in healthcare. We then collated all transcript excerpts that were coded as one or more of these sub-codes and summarized each category. Two authors (CB, SAK) iteratively reviewed and sorted the summaries, evaluating theoretical saturation within each category of the data and identifying areas of overlap where sub-codes could be grouped together [23], then produced a preliminary list of domains describing the predominant ways interviewees experience respect or a lack thereof in healthcare, which were sorted by whether the domain focused on individual versus organizational actions. These domains were further refined through discussion with the full author team. We did not identify major distinctions between sub-groups of interviewees and therefore present results collectively, except where Spanish speakers discussed language and interpretation, as noted in the results below.

Throughout our analyses, the author team sought to reflect on our positionality, recognizing that our situatedness cannot fully be separated from our analyses. The author team included women and men based in Seattle, WA and Denver, CO with professional backgrounds in medicine, law, and bioethics at a range of career levels, including some who were directly involved with the CHARM study and others who were not. The first author is a mixed-race, native English-speaking cisgender woman attending medical school. The senior author and principal investigator is a White, native English-speaking cisgender woman trained in law and bioethics who was also a co-investigator on CHARM involved in patient stakeholder activities.

## Results

### Interviewee characteristics

We conducted 40 interviews out of 71 invited (56%); of those who were invited but did not complete an interview, four actively declined, citing lack of time or interest, ten scheduled an interview but did not answer when we called to conduct the interview and did not respond to requests to reschedule, and the remainder did not respond to our email or phone outreach attempts. One-quarter of our interviews were conducted in Spanish. Most interviewees identified as female (88%) and either Hispanic/Latino(a) (43%) or White or European American (38%). Detailed demographics are shown in Table 2. Interviewees identified a range of activities at the individual and organizational levels that demonstrated respect in healthcare.

**Table 2:** Interviewee characteristics (n = 40).

	n (%)
**Recruitment site**	
Denver Health	20 (50)
Kaiser Permanente Northwest	20 (50)
**Preferred language**	
English	30 (75)
Spanish	10 (25)
**Mean age (range)**	36 (23–49)
**Gender**	
Female	35 (87.5)
Male	3 (7.5)
No response	2 (5)
**Race/ethnicity**	
Asian	4 (10)
Hispanic/Latino(a)	17 (42.5)
Middle Eastern or North African/Mediterranean	1 (2.5)
Native Hawaiian/Pacific Islander	1 (2.5)
White or European American	15 (37.5)
Multiple responses (American Indian, Native American, or Alaska Native and White or European American)	1 (2.5)
Prefer not to answer	1 (2.5)
**Highest level of education**	
Less than high school	5 (12.5)
Some high school, no diploma	2 (5)
High school diploma, GED, or equivalent	6 (15)
Some post-high school training, no degree or certificate	8 (20)
Associate college degree, or completed post-high school training with degree or certificate	5 (12.5)
Bachelor’s degree	8 (20)
Graduate or professional degree	4 (10)
No response	2 (5)
**Annual household income**	
Less than $20,000	8 (20)
$20,000 to $39,999	11 (27.5)
$40,000 to $59,999	9 (22.5)
$60,000 to $79,999	4 (10)
$80,000 to $99,999	1 (2.5)
$100,000 to $139,999	3 (7.5)
$140,000 or more	2 (5)
No response	2 (5)

### Individual efforts

Interviewees described two overarching types of activities that demonstrate respect from individual clinicians: engaging with patients and being transparent. Exemplar quotes are shown in Table 3.

**Table 3:** Exemplar quotes–individual efforts that demonstrate respect in healthcare.

Domain	Exemplar quotes
Engaging with patients	*“When I feel respected is when the provider actually sits down and looks at me and talks to me*. *They don’t act rushed or at least have some perception and that they’re really there to listen to kind of my chief complaints or concerns or worries*.*” (Participant 123*) *“I am pretty obviously a woman of color*. *I look Asian*. *I sometimes feel like I don’t have as much of a rapport with clinicians as I might have if I were white*.*” (Participant 126)* *“I like when they listen and kind of include me in I guess the decisions that are being made*. *Not just telling me*, *‘You need to do this and this and this*.*’… My doctor says*, *‘Well*, *you know*, *how about if we try this*? *What do you think*?*’ I guess having the patient participate in the care as well and not the doctor just making all the decisions*.*” (Participant 122)*
Being transparent	*“I feel more respected when people are just honest and look you straight in the eye and tell you what the conditions or what their concerns or what you need to do get better*. *Instead of going around the bush and saying*, *‘Well*, *maybe you could try this*,*’ or*, *‘Maybe if you try that*.*’ I’d rather just be frank and honest and I find that more respectful than beating around the bush*.*” (Participant 113)* *“I know that in school doctors are told to use the proper terms for things*, *but it’s frustrating when they don’t want to break it down into layman’s terms for you*.*" (Participant 154)*

#### Engaging with patients

Most interviewees said they perceive respect when clinicians listen to them and show empathy, including taking their symptoms seriously, answering their questions, and exploring alternative explanations for why they are experiencing a health problem. Specific actions to show respect included maintaining eye contact, acknowledging family members, and generally having good “customer service” skills. On the other hand, lecturing to, dismissing, or otherwise not hearing a patient could convey a lack of respect, whether due to the clinician’s attitude or not having sufficient time. A few interviewees described having been treated with a lack of respect due to their racial or ethnic identity or their personal choices, such as being a former cigarette smoker. About half of interviewees also discussed clinicians engaging with them in decision-making. They emphasized the importance of not feeling forced to make a decision, instead having enough time to thoroughly review the relevant information and working together to reach a decision. Through this process, they wanted clinicians to recognize and account for their preferences and empower them to make their own choices.

#### Being transparent

About half of interviewees said transparency was important for demonstrating respect, for example by being forthright about a patient’s likely health outcomes. Conversely, a lack of transparency could contribute to a perceived lack of respect, for example if a clinician did not admit they were uncertain or did not give an accurate sense of the time a procedure or recovery would take. For some, an important part of feeling respected was making their care understandable by meeting them where they were in their processing of their condition. This included explaining procedures and diagnoses—including risks and benefits—clearly and concisely, providing relevant information about a patient’s care, and clarifying next steps.

### Organizational efforts

Interviewees described five types of organizational efforts that show respect: promoting safety and inclusivity, protecting patient privacy, communicating about scheduling, navigating financial barriers to care, and ensuring continuity of care. Exemplar quotes are shown in Table 4.

**Table 4:** Exemplar quotes–organizational efforts that demonstrate respect in healthcare.

Domain	Exemplar quotes
Promoting safety and inclusivity	*“There’s no reason to misgender someone*.*… There should be policies that make sure that LGBT communities feel safe and that they’re able to be safe within an institution*. *Because if you can’t trust your medical providers*, *who can you trust*.*” (Participant 114)* *“A lack of respect would be to not help other people who come from countries where neither Spanish nor English is spoken*, *because we’ve seen a lot of people who come here*, *for example*, *from Guatemala where there are other languages and no*, *I mean*, *it’s very difficult to obtain medical attention for them*.*… And it would be a lack of respect to look down on someone else or give them a dirty look*.*” (Participant 127)* *“For them to*, *since I don’t speak English*, *bring me a translator*. *Almost always in [my clinic]*, *it’s almost always in person*, *never by phone call*, *and that kind of makes me feel more sure about what I’m understanding and of what I’m trying to say*.*” (Participant 153)*
Protecting patient privacy	*“I think compliance with HIPAA is very respectful*. *My healthcare information should be private and so when they honor that*, *whether it’s calling my name back to take me back to the office where they keep my chart*. *And even when you’re picking up prescriptions*, *just being mindful of my privacy*.*” (Participant 123)* *“Yeah*, *just like patient privacy*, *like HIPAA*, *that sort of thing*. *Not being able to disclose things to family members or friends or whatnot without permission from the patient*.*” (Participant 116)*
Communicating about scheduling	*“One example is that my [family member] had this pain and he went to the clinic and they told him that they were going to run some tests on him and*, *well*, *they never called him to—they were going to call him to make an appointment in another clinic and they never called to schedule it*. *He called them and they told him to wait because they had said they would call him*, *but they never did*.*” (Participant 127)* *“Communicating well*, *keeping me up-to-date if there’s a long wait for whatever reason*, *just letting me know*, *not just leaving me sitting in the waiting room forever*.*” (Participant 131)*
Navigating financial barriers to care	*“If a hospital’s focused on cost cutting*, *they’re going to really try to limit the amount of time that providers spend with patients*, *for example*. *So that’ll cut into how much time I have to spend with a provider*, *and that’ll affect how the provider handles our consult*.*” (Participant 126)* *“I felt that when I didn’t have insurance*, *I was basically just*, *I guess*, *a burden to them at that point without a doubt*. *I ended up with $6000 of medical bills because I had to go back into the ER multiple times to get a skin infection taken care of*.*” (Participant 114)*
Ensuring continuity of care	*“I didn’t have a consistent person that I saw other than the doctor who did my ultrasounds*. *But he deliberately made the choice to be like*, *‘I’m going to do her ultrasounds*. *I don’t want anyone else doing them*.*’ I didn’t know at the time that you can do things like that*. *I just thought you had to go with whoever they gave you*. *So it was really nice having a consistent person who saw you*.*” (Participant 106)* *“Instead of repeating or telling the doctor*, *‘This is what I have*,*’ having them know exactly what I have and not repeating the information myself*. *That’s awesome*. *“(Participant 161)*

#### Promoting safety and inclusivity

Most interviewees discussed the importance of safe and inclusive spaces for feeling respected in healthcare. A few identified programs and policies that promote inclusion, safety, and comfort, while several others described times when they or others felt unsafe because of a lack of policies to protect patients who are from historically marginalized communities. We asked interviewees to reflect on experiences when they might have felt a lack of respect in healthcare based on an identifiable characteristic. Those who reported having experienced this referenced characteristics including gender, race/ethnicity, beliefs (e.g., culture, religion, values), weight, education, sexual orientation, and attire. Interviewees discussed how treating others inequitably or making assumptions about a patient would show a lack of respect. A few also mentioned the importance of recognizing and understanding patients’ cultures, including religion and national background, when working with their communities. Further, half of Spanish-speaking interviewees explicitly mentioned the importance of language in their perception of respect. These interviewees said they felt respected when clinic staff tried to speak in their preferred language or provided an interpreter; one noted how having an interpreter helps make them feel more sure of themselves in healthcare interactions. Conversely, treating a patient poorly due to their difficulties speaking English would show a lack of respect, which a few English-speaking interviewees also discussed.

#### Protecting patient privacy

About half of interviewees discussed clinician-patient confidentiality and privacy protections such as those guaranteed in the U.S. under HIPAA [Health Insurance Portability and Accountability Act of 1996] as demonstrating respect. Several interviewees specified that privacy policies were important to establish that the patient and clinician are on the same page with regard to release of information to third parties, although one found these agreements to be a frustrating additional barrier. One interviewee said they appreciated the value placed on confidentiality among clinic employees, and another described feeling respected when a receptionist confirmed their information before their appointment to protect their privacy.

#### Communicating about scheduling

About half of interviewees identified communication about appointments, delays, and other scheduling issues as a component of respect. A few spoke generally about feeling respected when they received updates about appointments, and one discussed problems relatives have had with scheduling appointments. Some interviewees spoke about the timeliness of communication, mentioning that promptness felt respectful while delays, particularly when not communicated clearly, conveyed a lack of respect.

#### Navigating financial barriers to care

Some interviewees discussed financial barriers to healthcare in relation to feeling respected. They discussed how health insurance coverage and status affect both the patient’s access to care and the clinician’s ability to have enough time to interact with patients. While these interviewees described healthcare costs as a barrier, they recognized that they are typically out of the hands of an individual clinician. Instead, they perceived respect based on how their clinicians and healthcare institutions responded to this barrier—including treating them with care regardless of their ability to pay and recognizing how finances may affect patients’ decisions. For example, one person described a positive experience when they received care despite not having insurance and the clinic helped them get coverage. In contrast, another described the lack of respect they felt from being made to feel like “a burden” after accruing significant medical bills due to repeated misdiagnoses and multiple visits to urgent care while uninsured. Another pointed to the importance of incorporating costs into decision-making by noting they would feel a lack of respect if they did not get a full picture of the costs upfront.

#### Ensuring continuity of care

Some interviewees said they felt respected when they were able to be seen by the same clinician over time. These interviewees said it was important for their clinician to know their past medical history, with one commenting that they did not like to tell the same story repeatedly. Others discussed how a relationship could contribute to a sense of safety by ensuring their clinician knew them and their history and would be able to appropriately manage their care.

### Discussion

This exploratory qualitative study highlights individual and organizational efforts that may demonstrate respect to patients. Our findings emphasize the importance of respectful clinical relationships, as well as the interconnectivity of individual and organizational healthcare actors with regard to conveying respect in a way that promotes access to care and patient safety.

Our findings reaffirm work in other settings, as well as existing ethical guidelines for building mutually respectful clinician-patient partnerships [24], that have identified individual efforts toward communicating with empathy and honesty as central to respect. Prior studies have shown that patients perceive respect in clinical relationships when they are listened to, treated with honesty, and treated as “a person” and “an equal” [11,15,25]. As Beach and colleagues emphasize, patients understand “respect for persons” to be inclusive of, but broader than, simply respecting autonomy and obtaining informed consent. Our findings are similar; engagement and transparency were described as elements of respectful clinical relationships in their own right, but sometimes were also framed as key underlying factors to support informed decision-making about treatment options [26]. This supportive role of interpersonal relationships aligns with Ubel and colleagues’ description of the physician’s role in promoting patient autonomy by educating and helping patients “align their choices with their values” [27]. These impacts on relationships and decision-making illustrate that it is essential a patient feel respected by their clinician to effectively engage as a member of their healthcare team and feel empowered to advocate for themselves.

While clinicians’ actions are necessary to show respect, our findings suggest that respect is not always fully realized through individual actions. Organizational actions are integral to patient-perceived respect and may inform how interpersonal relationships take shape. Our findings highlight the critical role of institutional policies and procedures in supporting the development of collaborative, respectful relationships. Policies and procedures can create accessible opportunities for relationships to form by supporting and improving continuity of care, ability to schedule, and financial access, and can ensure patient safety by drawing boundaries on discriminatory behavior and privacy violations.

The impact of individual as well as organizational actions on patient-clinician relationships described by our interviewees has implications for how we might think about access to care. Access includes considerations of cost and affordability, and the extent to which clinicians can form meaningful connections with their patients that support patients’ willingness and ability to seek medical care [28]. Our findings related to individual actions that promote strong relationships and institutional policies that ensure patients can comfortably seek care suggest that patient-perceived respect may play a role in ensuring meaningful access to care. That is, an organization that works to embody a culture of respect may be more accessible to patients, insofar as it both supports respectful clinical relationships and promotes patient comfort. This is especially important for patients and communities who have historically lacked meaningful access to care and suffer negative health outcomes as a result. Future research should examine how respectful relationships affect access to care and health outcomes in different communities [29–34].

Our findings also highlight an overarching theme of patient safety, or protecting patients from harm [35], that is embedded within patient-perceived respect. Prior work on patient safety has focused on medical and administrative errors such as prescribing the wrong medication or breaching patient privacy [36,37]. Our results echo the importance of physical safety and privacy as elements of respect, but also underscore the necessity of psychological safety and comfort. A respectful patient-provider relationship may contribute to a sense of safety and comfort that patients will be treated by someone who knows them and their medical history. Further, our findings related to promoting inclusivity highlight the importance of psychological safety that for some interviewees was entrenched in feeling welcome and included in a healthcare setting. Some of our interviewees described having personally experienced, or having loved ones who had experienced, outright discrimination based on observed or presumed characteristics (e.g., skin color or gender), and several also discussed experiences feeling looked down on for characteristics including their weight or their status as a smoker, facing structural inequities such as a lack of appropriate translation or interpretation support, or experiencing more subtle forms of bias during activities like appointment scheduling. These examples illustrate that discrimination, racism, and other forms of bias in healthcare operate on both an interpersonal and structural level and warrant focused attention and decisive responses at each level. They also highlight a need for institutional policy efforts to incorporate attention to structural inequities like lack of access to high-quality translations and financial barriers to care, and to protect patients from subtle discrimination that occurs through microaggressions, or “brief and subtle” messages that “ambiguously disempower racial minorities” [38] and other historically marginalized social groups [39]. Additionally, cultural differences in patient-perceived respect should be recognized, and future research should examine cultural considerations for showing respect in healthcare.

### Clinical implications

Our findings highlight opportunities for clinicians and healthcare organizations to thoughtfully consider how they approach demonstrating respect for patients. Clinicians should enter patient interactions with a clear intention to build respectful partnerships and view each patient as a unique individual. Healthcare organizations should consider how to demonstrate respect for patients through their policies and practices, incorporating the perspectives of patients and community members to the extent possible. In particular, equitable access to care should be a priority, meaning that organizations should consider ways to address financial barriers, ability to schedule, and equity in appointment timing. Further, organizations should acknowledge the role of respect in promoting meaningful access to care and evaluate the relationship between improved experiences of respect and actualized access. Healthcare organizations should also prioritize responses to, and prevention of, discriminatory or biased behaviors among clinicians and staff, by implementing anti-racist and anti-discrimination policies and requiring ongoing education for members of their workforce about various forms of bias and their impact on patients. Finally, individual clinicians and organizations should seek out and be responsive to feedback from patients about how best to build a patient-centered culture of respect, for example by working to develop ongoing relationships with patient and community advisors. These efforts will require investment of time and resources, and some may require making trade-offs, but the role of patients’ experiences of respect in building clinical partnerships and promoting positive health outcomes suggests it is important to think critically about how to demonstrate respect in primary care settings.

### Limitations

This study has some limitations. Interviewees were patients at two clinical institutions with limited demographic diversity, with most identifying as female and either Hispanic/Latino(a) or White or European American, and only included English and Spanish speakers. Patients who receive care at other institutions, have different demographic characteristics, and/or speak other languages may have different perspectives. Additionally, interviewees were enrolled in an ongoing research study about hereditary cancer risk, which may have influenced their perspectives about their healthcare institutions. The fact that all interviewees had agreed to participate in a research study also raises the possibility that some individuals with negative experiences may not have been enrolled, and therefore interviewees may not represent all viewpoints.

## Conclusion

Our findings highlight key domains that reflect the interconnectivity between individual and organizational actions toward showing respect for patients, including the critical role of clinical relationships, meaningful access to care, and safe healthcare institutions. Our findings from this exploratory study offer important insight into opportunities for future research and quality improvement efforts to institute changes that better fulfill the ethical duty to respect patients.

## Supporting information

S1 File Interview excerpts by code. (DOCX)

S2 File COREQ checklist. (DOCX)
