# Supplementary material for: Patient perspectives on how to demonstrate respect: Implications for clinicians and healthcare organizations
Source: PLoS One. 2021 Apr 29;16(4):e0250999. doi: 10.1371/journal.pone.0250999 (PMC8084197; doi:10.1371/journal.pone.0250999)
Supplement: S1 File — (DOCX) [file pone.0250999.s001.docx]

**Interview excerpts by code**

Dedoose export of excerpts tagged under subcodes of ***Respect in medical care****: Characteristics or behaviors of healthcare providers or institutions that, in their presence or absence, convey respect or lack of respect*

**Appointment timing** : Provider being late/keeping patient waiting, being rushed or hurried in appointments (not including provider attitude), or institutional policies that affect provider time

Excerpt - Document: 127 Respect Interview - SP English translation - cleaned.docx, Position: 3161-3962

*So since you don’t have examples, what about if you could imagine for yourself something that would make you feel disrespected or that your friends or family members have been disrespected? If you can, think of examples. Or something that just wouldn’t make you feel respected.*

*S2 04:10 Yes. One example is that my [family member] had this pain and he went to the clinic and they told him that they were going to run some tests on him and, well, they never called him to-- they were going to call him to make an appointment in another clinic and they never called to schedule it. He called them and they told him to wait because they had said they would call him, but they never did. Those are things that don’t--*

*S1 04:32 Yes. Right, that --*

*S2 04:34 --that wouldn’t be good. But that’s not the case.*

Excerpt - Document: 114 Respect Interview-cleaned.docx, Position: 4928-6829

*And this sort of goes to a couple of the issues that you’ve brought up but I wanted to ask it explicitly. Have you ever felt the lack of respect in the medical setting based on who you are, what you look like, what you believe, any of those sorts of traits?*

*S2 10:23 In a study?*

*S1 10:24 In medical care or in a study, I guess, either way.*

*S2 10:28 This is actually my first study but in medical care, yeah, I have felt sort of ignored. My concerns are kind of brushed off and when I was bringing certain things to the forefront saying, “Hey, I think this may be what my issue is,” they kind of just brushed me off and went, “Well no. I know that’s not a problem. This isn’t this.” They didn’t do any tests. I mean, there was plenty of times that I left the ER after experiencing severe stomach pain and they’re just like, “Well, we don’t know what the issue is. Go home.” And of course, that was when I didn’t have insurance. I felt that when I didn’t have insurance, I was basically just, I guess, a burden to them at that point without a doubt. I ended up with $6000 of medical bills because I had to go back into the ER multiple times to get a skin infection taken care of. The first time I actually went into the ER, I had gone in with a temperature of 106 degrees and I waited in the empty waiting room for four hours in the morning. And when they brought me back, my temperature had already dropped and when I told them what it had been, he’s like, “Really, are you sure you didn’t read it wrong?” So I was a little upset. But then they also didn’t run a proper test and so they didn’t find out what it was that was giving me the infection so they didn’t give me the proper antibiotics.*

*And then the next time they gave me the wrong antibiotics that didn’t have good kidney penetration so it wasn’t getting rid of the kidney infection. So I felt there was a lot of oversight in that instance.*

Excerpt - Document: 101 Respect Interview-cleaned.docx, Position: 394-1004

*So could you give me some examples of the sort of things that make you feel respected in your medical care?*

*S2 02:53 I guess just when they actually diagnose the problem right makes me feel respected. But it’s kind of one of those things where sometimes it feels like they’re guessing more than they’re actually diagnosing. Sometimes I may just feel-- when you feel like they’re guessing, you don’t really feel that much respect than them trying to take the time. But there are a lot of doctors out there-- my doctor now takes a lot of time and a lot of care in trying to figure out what’s really going on, so.*

Excerpt - Document: 101 Respect Interview-cleaned.docx, Position: 5720-6814

*has there ever been an encounter in the past with a previous doctor or someone else where you experienced a lack of respect?*

*S2 10:42 Yeah. I mean, I’ve had some emergency room doctors in the past where you go in and it feels like they’re just trying to kick you out. They don’t want to really deal with your issues of what’s really going on. So I have had that where it just seems like they got so much going on that they try to just quickly diagnose you. I mean, the first time I went in for the [issue related to my surgery], for example, they tried to diagnose it as just [being related to a preexisting condition], and they wouldn’t do a second ultrasound. They ended up sending me home with some Zantac. And then two days later, I had to go back to the emergency room, and they finally decided that, yeah, [I needed to have surgery]. Even though the first time I was in, the CAT scan showed [the affected area], and the doctor said that they knew that it needed to be taken out. But the surgeon said that they couldn’t see clearly enough on the ultrasound, so they weren’t going to do it.*

Excerpt - Document: 118 Respect Interview-cleaned.docx, Position: 387-936

*So could you give me some examples of the sorts of things that make you feel respected in your medical care?*

*S2 02:40 I think number one, being the provider being on time or at least communicating if there's a delay with the appointment. I think that that's huge. I think that providers taking the time to acknowledge that-- especially, if you've been through something kind of scary or traumatic, taking the time to acknowledge that and answer your questions maybe more in-depth then what you might even be aware that you have deeper questions on.*

Excerpt - Document: 118 Respect Interview-cleaned.docx, Position: 1508-2077

*can you think of any policies that the medical institution could have in place that would show respect?*

*S2 04:54 Any policies.*

*[silence]*

*S2 05:13 As far as the institution goes, I think just having plans in place for if a provider is late or needs to cancel. Just making sure that there's a good communication, checking in as well, I think, too to make sure-- surveys to see possibly how your appointment went if there was any concerns that you felt that should have been addressed that maybe wasn't. I think that's kind of what I'm thinking at least, at this point.*

Excerpt - Document: 118 Respect Interview-cleaned.docx, Position: 2145-2956

*could you give me some examples of the sorts of things that might make you feel a lack of respect from medical care?*

*S2 06:18 Are you referring to doctors in general or the whole clinical experience from when I get there to when I leave?*

*S1 06:29 All of it. Whatever [laughter] comes to mind for you.*

*S2 06:31 Okay. I feel that if I feel like I'm not being listened to if I feel that-- just providers, nurses, receptionists, check with me-- check you in, just having an attitude. They're not happy to be there or they've got something going on and it shows when you go to check-in. Just overall, not listening to concerns as being someone who's there because there's an issue. Feeling rushed a lot. The providers checking their watch or-- you just feel like you're being hurried out, is I think, big as well.*

Excerpt - Document: 161 Respect Interview-cleaned.docx, Position: 383-1576

*could you give me some examples of the sorts of things that make you feel respected in your medical care?*

*S2 02:52 Oh, yes, exactly. Yeah. Especially during my doctors' visits, having our providers or pretty much my doctor know exactly what I have instead of having myself repeat for the second time or third time what I have and my condition. Instead of repeating or telling the doctor, "This is what I have," having them know exactly what I have and not repeating the information myself. That's awesome. I really like that with my providers, and my providers do know me. They know exactly what I have, and I don't have to repeat the information. So it's really helpful that way. You save time, and we go straight to what needs to be done. I really like that from my providers knowing that they know what I have. And even though we met for so long, they still know what I'm going through, what I need, and so that's really awesome. And especially, it does help when you see the same provider. That really helps a lot too. When you get to see the same provider, you get to establish a patient-provider relationship, and that's really awesome. It's also really helpful, and it helps your care.*

Excerpt - Document: 136 Respect Interview-cleaned.docx, Position: 419-869

*can you give me some examples of the sorts of things that make you feel respected in your medical care?*

*S2 03:21 Let's see. Being waited on time. That's a pretty big one. If I'm not feeling well I'd like to be seen as soon as possible. And being explained everything, especially me. I like to look up a lot of things to get more information and it's really helpful when the doctor explains more in-depth. And I think those two are the primary, yeah.*

Excerpt - Document: 136 Respect Interview-cleaned.docx, Position: 1558-2082

*If you feel you're struggling with things that you know that they do find respectful sometimes it is helpful to talk about the things that you don't find respectful. So maybe something that they did or that wasn't respectful, you could talk about as well, for sure.*

*S2 05:29 Okay. Well, I've mostly had good experiences. The only downside would be when they go into emergency it usually takes a long time to be seen.*

*S1 05:45 Okay. So it sounds like maybe wait times are an important thing of respect for you?*

*S2 05:50 Yeah.*

Excerpt - Document: 136 Respect Interview-cleaned.docx, Position: 3226-3632

*Could you now give me some examples of the sorts of things that would make you feel maybe a lack of respect in your medical care?*

*S2 07:54 Yeah. Maybe getting my information wrong, like my name or my chart. Not getting my questions answered. Kind of getting brushed off. Wow, there's so many examples. Having an even longer wait time [laughter]. Or having rude doctors or nurses. Is that enough [laughter]?*

Excerpt - Document: 136 Respect Interview-cleaned.docx, Position: 5734-6275

*Can you imagine anything that might make you feel a lack of respect in a medical setting or maybe your friends and family have experienced things that you thought were disrespectful in a medical setting? Anything you could share there?*

*S2 03:08 Well, my husband and my daughter are the only ones that have the same insurance. And, no, I've never had any complaints. I mean, I could imagine I would be angry if another family came after us and they got attended first. That would make me pretty mad, but that's never actually happened though.*

Excerpt - Document: 136 Respect Interview-cleaned.docx, Position: 19168-19820

*so if you could just go into why you answered those questions that way?*

*S2 23:51 Yeah. So I gave four to the medical field because of the wait time. That's the only downside. Everything else is good. Just the wait time, like an emergency, is long. That's the only reason. And then, I mean, I've never had any personal experiences with other studies, this is the first study I [inaudible]. And like I had mentioned before, because of studies we know so much and I'm grateful for that. So I gave it a five. And then the CHARM study, I mean, you guys have been awesome with me, very professional, no complaints, so that's why I gave it a five [inaudible].*

Excerpt - Document: 122 Respect Interview-cleaned.docx, Position: 412-1009

*So could you give me some examples of sorts of things that make you feel respected in your medical care?*

*S2 03:11 I guess a doctor not feeling like they’re rushing me to get through to get to the next patient. I like when they listen and kind of include me in I guess the decisions that are being made. I’m not just telling that you need to do this and this and this when they-- like one of the things that my doctor says, “Well, you know, how about if we try this? What do you think?” I guess having the patient participate in the care as well and not the doctors who’re making all the decisions.*

Excerpt - Document: 122 Respect Interview-cleaned.docx, Position: 1853-3455

*o now I kind of want to flip those questions on their head and ask you to give me some examples of the sorts of things that make you feel a lack of respect when it comes to your medical care?*

*S2 00:00 Recording back up.*

*S1 00:01 I do. All right. And we should be getting on. I’m so sorry about that.*

*S2 00:06 That’s okay. It happens. Well, I mean, I guess I can-- one thing that happened recently was actually with my son was I had taken him in for an urgency care appointment. And I kind of felt like the doctor just came in and was kind of like, “Okay. What’s the problem?” I mean, I swear he probably spent-- and actually I think he was a PA but I think he probably spent less than five minutes in the room and threw some antibiotics at us and out the door, we went. So, yeah. So that would be something as of recent that happened with my son. But as far as my care, I don’t go to the doctor, I mean, a whole lot. I’m just not a-- I don’t go unless there’s a really good reason I guess and so I go to the doctor to maybe three times a year max. So I feel really fortunate with the care team that I have that everything always is addressed and I feel respected and, so.*

*S1 01:26 Okay. So it sounds like from that example that you gave, it’s kind of the opposite of the things that you had mentioned with the first question. In that instance, it did feel like you’re being rushed, maybe didn’t feel like the doctor was listening and that the decision was kind of made for you without really consulting you. Does that sound, right?*

*S2 01:47 Right. Yeah, yeah. Exactly. There was no empathy or anything.*

Excerpt - Document: 123 Respect Interview-cleaned.docx, Position: 365-1584

*And I’d like to ask you to give me some examples of the sorts of things that make you feel respected in your medical care?*

*S2 02:15 I think when people make eye contact and when the providers would actually sit down. I’m sorry. Just someone’s knocking on my office door.*

*[interruption/discussion omitted]*

*S2 02:50 So going back to the question, when I feel respected is when the provider actually sits down and looks at me and talks to me. They don’t act rushed or at least have some perception and that they’re really there to listen to kind of my chief complaints or concerns or worries. And then, I like them to use hearing and reflecting and they kind of like regurgitate what I said back to make sure what I’m saying is what they’re hearing. I like people with gifted sign manners so that they have compassionate thinking and doesn’t minimize concerns but still is very pragmatic. So I like them if they’re focused but at least validate, “I hear that you’re concerned or something,” is when I feel the most respected.*

*S1 03:47 Great. So it sounds like people who are really listening but also able to, you said, pragmatically sort of solve the problem that you’re there for. Is that right?*

*S2 04:02 Correct. Yeah.*

Excerpt - Document: 105 Respect Interview-cleaned.docx, Position: 825-1322

*And what about the other healthcare staff, like the nurses or maybe some of the reception staff?*

*S2 03:49 Reception staff, probably maybe they can be a little more quicker about making sure everyone's checked in or just more time management, rather than just being on the desktop computer or their personal cell phone or whatever. They should care more about the patients and getting them all checked in and making sure that they're on time or they're early. And making sure that everyone's happy.*

Excerpt - Document: 105 Respect Interview-cleaned.docx, Position: 3089-4279

*Can you imagine anything that, say, the nurses could do that might show a lack of respect?*

*S2 08:20 Let me think. I'm not sure. No, I can't think of anything right now.*

*S1 08:37 Okay. I know we talked about the reception staff earlier. Is there anything that you can think of? I believe you mentioned if they're maybe not paying attention to the patient as much or kind of distracted with their phones. Would that be a sign of lack of respect on their part?*

*S2 09:00 Yeah, definitely. Even if you're just-- have to speak with the receptionist for a couple of minutes, they should be also concerned and get you checked in on time or earlier, to be able to see your house specialist or your doctor, nurse, whoever you may need to see. It's just if they're lacking in respect or actually don't want to-- seem to care to get you checked in or they're just on their personal phone or they're just worried about other paperwork and not concerned about their patient that needs to get checked in. Then that can be an issue because then that's a time-- like I said, a time management thing. Because what if the patient has another appointment following right after that or something else going on.*

Excerpt - Document: 142 Respect Interview- cleaned.docx, Position: 462-1181

*So with that in mind, can you give me some examples of the sorts of things that make you feel respected in your medical care?*

*S2 03:02 Definitely having that one-on-one and having the feeling that they're listening and actually making that eye-contact with me. And I know, initially, when I first have a new physician or nurse practitioner, it's hard to have that trust right away. So I would say, just having that care and sympathy towards me and the time, not feeling like I'm being rushed during my assessment with them. Yeah. So asking any pertinent questions and making me feel like my information is not shared with other people, and that I can feel comfortable with them. So maybe that reassurance as well. Yeah.*

Excerpt - Document: 149 Respect Interview - SP English Translation - cleaned.docx, Position: 3983-5656

*are there regulations at the clinic that make you feel respected when you know about these regulations or how the clinic functions? Are there steps that they follow that make you feel respected?*

*S2 05:39 Well the truth is, the test would be, if I still remember, because [inaudible] (there was one) appointment, and I couldn’t get there. Then, I felt really bad when I spoke over the phone and explained the reason to them as to why I couldn’t get there. So, for me it didn’t seem right, because they set my appointment not until [month] when I had to see the doctor right away. In that sense, yes, for me, it did look bad, because they didn’t set it until [month] I mean, in that aspect, because it’s not your fault if you can’t arrive on time for the appointment. Do you know what I mean? And, after that they set my appointment not until two months after, and no, that’s not fair to me, that it can’t be set earlier, not up until two months after, as it was in my situation. I don’t agree with that.*

*S1 06:50 Well, that’s a– like something that makes you feel not respected at the clinic, that they made you wait two months for an appointment.*

*S2 07:01 Well, I– I mean that setting policies around regulations that are dumb regulations. I mean, to have to wait up to two months for them to set another appointment. I mean, setting an appointment and those regulation policies, well, no. Well, for me it’s not at all right.*

*S1 07:24 Sure. Sure. No, well, yes. If you have to see the doctor immediately, and then you have to wait, like you said, two months, that’s a long way off.*

*S2 07:33 Exactly. But for that policy, to fix it or not, it doesn’t seem fair to me.*

Excerpt - Document: 108 Respect Interview-cleaned.docx, Position: 3962-4686

*Are there other things—actually, I guess you said that both of those examples made you less interested in engaging with medical care going forward. I’m curious if you could say a little bit more about that.*

*S2 07:51 I mean, in general, I guess I don’t have a super high opinion of most medical care providers. I mean, it’s not necessarily the individual but I just feel like in our culture, it’s really driven by money a lot and by insurance and that people aren’t always getting the best care because either the care providers are overworked. They don’t have enough time to really sit and listen to what’s going on to really know who they’re dealing with. It’s just sort of in and out, and so-- what was the question again?*

Excerpt - Document: 108 Respect Interview-cleaned.docx, Position: 4700-5485

*I just asked you to expand a little bit on, I guess, how those examples of not feeling respected made you feel about the medical setting [crosstalk].*

*S2 08:52 Not the ones I interact with, yeah. So, yeah. When you go in and they like rather than knowing who you are and why you’ve made a decision, or what education you’ve gotten before you made a decision, or like really listening to where you’re coming from. It’s just like blanket information that they give to every person which really is-- because we don’t have the time to get to know you or remember you because they’re seeing so many people and just the whole system is like overworked. And it does often feel like, “How many people can we get in and out of here?” instead of like, “Are we truly caring for these individuals?”*

Excerpt - Document: 126 Respect Interview-cleaned.docx, Position: 1583-2287

*And so kind of beyond the doctors or providers themselves and thinking more largely to maybe the nursing staff and even some of the reception staff, can you think of any examples of how they show respect?*

*S2 05:06 I guess maybe a big thing is being respectful of my time. If I’m waiting a while-- I guess that’s not really something that they’re usually in control of though. That’s maybe a bigger administrative or a system-wide thing. But thinking back to, I guess, my interactions with medical assistants, I feel like a big part of what helps me feel respected is just how they interact with me. If they are smiling or just being really friendly and approachable, that’s a big part of it for me. Yeah.*

Excerpt - Document: 126 Respect Interview-cleaned.docx, Position: 2515-3382

*what sorts of policies that the medical institution has in place that would show respect. So you kind of jumped to my question there a little bit, but [inaudible]--*

*S2 06:29 Yeah, okay. So I guess to address the administrative part, I just feel like just healthcare, in general, is really focused on cost cutting and finances, and that just seems, in a lot of ways, to be not conducive to making patients feel respected. Those two goals seem to be at odds in a lot of ways. Because, I mean, if a hospital’s focused on cost cutting, they’re going to really try to limit the amount of time that providers spend with patients, for example. So that’ll cut into how much time I have to spend with a provider, and that’ll affect how the provider handles our consult. If they’re under time pressures and-- yeah. I mean, that could affect a lot of things about the encounter.*

Excerpt - Document: 156 Respect Interview-cleaned.docx, Position: 1623-2092

*but are there anyone else-- say, whether it's the intake nurses or the reception staff or just other people that are there in the clinic or the hospital, is there anything that those folks could do that would show respect?*

*S2 04:55 I don't know. No. I've had pretty good experiences with that kind of stuff. I think the same thing, just listening, maybe slowing down a little bit, not being in such a rush for intake nurses and everything, but I think that would be it.*

Excerpt - Document: 156 Respect Interview-cleaned.docx, Position: 2474-2747

*And then you mentioned earlier the kind of slowing down or not rushing through with the intake. Do you see that as sort of a common problem in your medical care? And if so, is that something that maybe you think would benefit from some changes?*

*S2 06:37 Yes and yeah, I do.*

Excerpt - Document: 158 Respect Interview - SP English translation - cleaned.docx, Position: 1200-1515

*Could you give me some examples of the things that make you feel respected in your medical care?*

*S2 03:21 I like it a lot, and I go to my clinic because they respect my schedule, they respect my privacy. I’m at ease because they serve me well, they are respectful to me. I’m comfortable, due to many things, really.*

Excerpt - Document: 158 Respect Interview - SP English translation - cleaned.docx, Position: 1783-2124

*As an example of how they treat me or what it’s like? When you make an appointment, I make an appointment, they make it quickly or as quickly as possible. That’s something that I love, that when I call the clinic, if they don't have it for that same day, they give it to me for another day or so, as quickly as they can, they make it for me.*

Excerpt - Document: 133 Respect Interview-cleaned.docx, Position: 2768-3385

*could you give me some examples of the sorts of things that make you feel respected in medical care?*

*S2 03:11 Yeah. So, the doctors and the medical staff being on time, getting prompt visits definitely makes me feel respected. Just getting the information timely, if I had test done I do want some information quickly so that I can see what my results are. That’s mainly what I feel would-- yeah, as far as doctors go, I don’t see very many doctors. So I would say I’m a pretty and generally healthy person. So there’s not many occurrences where I have gone in and did not feel respected on my visit with a provider.*

Excerpt - Document: 133 Respect Interview-cleaned.docx, Position: 3503-3951

*And so could you maybe go into those a little bit more and tell me what it is about that exactly that makes you feel respected?*

*S2 04:28 Yeah. It makes me feel like my time is valued as well. And I know in doctors you have the higher [inaudible] and whatnot, and their time is valuable as well. But just going that extra mile and staying on time or giving me that prompt information, makes me feel like my time or my health issues are top priority.*

Excerpt - Document: 157 Respect Interview-cleaned.docx, Position: 382-993

*So could you give me some examples of the sorts of things that make you feel respected when it comes to your medical care?*

*S2 02:33 Let's see. Maybe just basic stuff to start with like when they ask you what you want to be called by and then they ask you your opinions or just maybe options regarding your healthcare or just different thoughts you have. Maybe they pay attention to you, and they give you enough time to ask all your questions, and they don't rush you, and they don't forget everything you say. They addressed every part of what you said. Yeah. That would be some things to start with, I guess.*

Excerpt - Document: 157 Respect Interview-cleaned.docx, Position: 2517-3514

*o you kind of touched on some aspects of the next question that I wanted to ask, but could you give me some examples of the sorts of things that would show a lack of respect? And I know that you just mentioned a lack of eye contact or people being in a bad mood. Was there anything else that you can think of?*

*S2 07:15 Let's see. Yeah. I mean, lately, I've just had good experiences, I think, but whenever someone's in a bad mood, you're definitely not sure if they're just having a bad day, or you just kind of feel like they're taking it out on you. You want to be in a good mood when you're discussing your health stuff even if they're having a bad day. And I think just following up to make sure you have any questions afterwards and giving you lots of chances to ask questions. Because sometimes things can be really busy, and you might forget to ask questions or something like that, and that kind of can be frustrating. Well, yeah, just feeling like you're not rushed is probably a big one.*

Excerpt - Document: 143 Respect Interview-cleaned.docx, Position: 385-1416

*So could you give me some examples of the sorts of things that make you feel respected in your medical care?*

*S2 [02:41] I mean, I always appreciate at the beginning that everybody always double checks they’ve got the right patient [laughter] before they get going. There’s probably a name that sounds very similar to mine, and I’ve gone in and then found out I wasn’t the right person. So that’s always a good thing to do [laughter]. Most recently, something that did kind of bother me at a recent doctor’s visit, in the lobby in the waiting room, there’s a chart with all the doctors and if they’re late, by how long. My doctor’s thing said it was on time. It never got changed. And I still waited about 45 minutes before my appointment. And this was an early morning appointment, so I wasn’t expecting it to be a delay yet. So that was a bit frustrating. Let’s see. Other things that I find respectful. Well, when the chart is updated, I find that more respectful, I guess. Having trouble thinking of other specifics right now.*

Excerpt - Document: 143 Respect Interview-cleaned.docx, Position: 3522-4187

*But could you give me some examples of the sorts of things that might make you feel a lack of respect in medical care?*

*S2 [02:33] Like I said previously, when there’s a delay and there’s no communication about the delay, that’s frustrating because I know that they’re time is important, but mine might be, too. And let’s see. [inaudible]. I guess if they just come in, they call your name in the waiting room and then they just bring you to the doctor’s office and there’s no communication at all between that, it feels like they don’t want to be there. And I mean, I know it’s their job and they might not want to, but that makes me feel a little more stressed.*

Excerpt - Document: 106 Respect Interview-cleaned.docx, Position: 5320-6039

*Could you give me some examples of sorts of things that make you feel a lack of respect when it comes to medical care? And I know we touched on a few of them, but if you have any other ones, please feel free.*

*S2 09:42 Yeah, I think just making it seem like they don't have time for you. That they just have a million other appointments coming through, so. They kind of seem hurried sometimes. So that makes it feel like there's no respect. Yeah, they're not being able to be truthful and admit when you don't have the answer, you've never dealt with something like this before so you'll have to consult. To not being upright, honest. And then, yeah, the lack of follow-up because they just switch people so frequently.*

Excerpt - Document: 131 Respect Interview-cleaned.docx, Position: 1474-2136

*can you think about how the other individuals that you might interact with, say, the nurses or the reception staff, can you think of any ways that they might show respect?*

*S2 04:25 Well, I think good customer service skills, obviously, which I know is really hard after long days and things. But [laughter] good customer service is one. Again, communicating well, keeping me up-to-date if there’s a long wait for whatever reason, just letting me know, not just leaving me sitting in the waiting room forever [laughter]. Maybe just, I guess, feeling acknowledged, listened to, and that I’m not just another number in the line of the list of people to get through.*

Excerpt - Document: 145 Respect Interview-cleaned.docx, Position: 431-1115

*So could you give me some examples of the sorts of things that make you feel respected in your medical care?*

*S2 02:48 I think the number 1 thing that's important to me especially if you're going to see the doctors since I'm someone who doesn't seek out medical care very often is in just knowing that my provider is there and going to listen to all of my concerns. And then not feeling rushed during that time because a lot of the times I go, I know you have another appointment right after. But sometimes it can come off as dismissive. So I like having that at least demeanor that I'm being heard, and that all my questions are being taken seriously and answered thoroughly, I guess.*

Excerpt - Document: 145 Respect Interview-cleaned.docx, Position: 2110-3095

*So now, I want to kind of flip all those questions on their head and ask you if you could give me some examples of sorts of things that might make you feel a lack of respect from your medical care?*

*S2 05:15 Not following up in a timely manner if you're waiting for any type of results. I don't know if it's disrespectful, but I think it goes back to what I was saying before. Just making sure that someone gives you time and then again, that feeling where actually makes me feel like my issue is not necessarily important. And not having a professional demeanor. I mean, I know we all have bad days, but sometimes people get snappy and healthcare is a stressful job. Let's be honest. So I get it to some degree but not taking that out on your patients.*

*S1 06:09 Okay.*

*S2 06:19 Let's see. And I think if you don't get a full picture of what medical costs are going to be. So I guess not being thorough and transparent and sort of fees-- or again what's happening and why it's happening.*

**Continuity of care**: Seeing the same provider across visits

Excerpt - Document: 114 Respect Interview-cleaned.docx, Position: 2003-2924

*Can you think of any experiences you’ve had where you felt, “I feel really respected in this moment in medical care?”*

*S2 05:08 Yeah. Actually with my primary care physician. She is extremely good at listening to me and asking the right questions and leading me to-- basically, I several times come in and say, “I think this may be the issue and she doesn’t discount what I say. She will think and say, “Okay. We’ll check it out if that may be the issue.” If she doesn’t believe it’s an issue then she’s like she’ll explain it very clearly as to why it shouldn’t be something I worry about. I feel not trying to talk above my head basically. She has learned basically where I am as a person, how I am able to communicate, and so I feel like she’s put in that effort to know me as a patient. And so because of that, I really enjoy being with her and I feel like she respects me as a person and not just as another patient.*

Excerpt - Document: 154 Respect Interview-cleaned.docx, Position: 2573-3194

*So I was curious if you could kind of give me an idea of the sorts of things that make you feel a lack of respect.*

*S2 05:38 I feel like I kind of just laid that out. It's kind of the same thing, I mean, regardless of who it's coming from, dismissive behavior, maybe not making eye contact. Oh, kind of passing the bucks and, "It's not our responsibility. It's somebody else's responsibility," that's frustrating. That's something I've dealt with before. Yeah. I mean, even if it's not your responsibility to do something or find the answer to something, it'd be nice to say, "Hey, let me get you somebody who can." Right?*

Excerpt - Document: 161 Respect Interview-cleaned.docx, Position: 383-1576

*could you give me some examples of the sorts of things that make you feel respected in your medical care?*

*S2 02:52 Oh, yes, exactly. Yeah. Especially during my doctors' visits, having our providers or pretty much my doctor know exactly what I have instead of having myself repeat for the second time or third time what I have and my condition. Instead of repeating or telling the doctor, "This is what I have," having them know exactly what I have and not repeating the information myself. That's awesome. I really like that with my providers, and my providers do know me. They know exactly what I have, and I don't have to repeat the information. So it's really helpful that way. You save time, and we go straight to what needs to be done. I really like that from my providers knowing that they know what I have. And even though we met for so long, they still know what I'm going through, what I need, and so that's really awesome. And especially, it does help when you see the same provider. That really helps a lot too. When you get to see the same provider, you get to establish a patient-provider relationship, and that's really awesome. It's also really helpful, and it helps your care.*

Excerpt - Document: 161 Respect Interview-cleaned.docx, Position: 1610-2723

*So interesting, one of the things that kind of jumped out to me about the things that you just mentioned was this idea of providers coming into an appointment or a meeting prepared and ready to kind of just dive right in.*

*S2 04:46 Exactly.*

*S1 04:47 So that's awesome. All right.*

*S2 04:49 Yeah, exactly. Yeah. And it really does help when you get to see the same doctor that's taking care of you because that does help with your care move smoothly and forward. And I do understand that sometimes it's not possible. I totally get it when-- and it has not been when I get to see other providers because they might not have appointments available, or they're out, or you get to see someone else because it's emergent or urgent, and you need to see someone else because the actual provider that you normally see is not available. And that's totally fine. I mean, I totally get it, and that has happened. But, yeah, it's really awesome when you get to see the same provider that you're assigned to, and they're the ones that know everything pretty much, and they're seeing it through. And it just makes it a lot easier.*

Excerpt - Document: 136 Respect Interview-cleaned.docx, Position: 2098-3167

*So what about any policies that a medical institution might have in place? Can you think of any policies that would show respect by chance?*

*S2 06:06 Oh, yes. Recently my daughter had gotten chicken pox and I wanted to make sure it was chicken pox, and I wanted to be seen by her pediatrician. And at first, one of the nurses had told me that it was not impossible to go to, to get seen, but since it's contagious that she recommended me not going in. And I had asked her if there was any way that we could still be seen? Maybe if there was a-- if there was a contaminating room that she can go into and she had to go and ask a doctor to see if that's something that could be done and eventually it was, but I had to ask her. Because at first she kind of said no. So that was, I guess, a little disrespectful.*

*S1 07:18 Okay.*

*S2 07:18 I mean, there should be a policy for that.*

*S1 07:22 Okay. So it sounds like there was a policy may be for it, but it wasn't communicated to you, is that right?*

*S2 07:32 It wasn't really communicated to the nurse because she had to ask.*

Excerpt - Document: 146 Respect Interview- cleaned.docx, Position: 426-4844

*So with that in mind, can you give me some examples of the sorts of things that make you feel respected in your medical care?*

*S2 03:05 Well, my personal experience is the ones that actually listen to what my concerns are, not what they think my concerns are, the ones that actually take in why I'm there. And then the ones that actually take that and - how do I say this? - try to help solve the issue instead of just plopping a Band-Aid on it and saying, "Oh, it's just this. You're fine," when then I have to go back and get a second or third opinion. And the second and third opinion don't match the first opinion, and they're calling the doctor idiot or whatever.*

*S1 03:52 Okay. So could you expand on that a little bit, and I want to just ask what it is about that that makes you feel respected.*

*S2 04:02 Well, for me, personally, I've battled some ovary cyst issues the last few years. We’re not sure if it's my IUD or not, but it's caused other symptoms. So my biggest concern was I wanted my IUD out. My first and second doctor, including my OB, were like, "No. That's not it. You're just gaining weight just because you're not active," and this and that. I went and saw a new doctor, same [healthcare institution] clinic, and a new OB, and they go, "Yeah. You're not as active as you were, but your IUD is clearly playing a role in your problem." Then they asked me-- the new two doctors asked me, "Okay. Well, what are your other symptoms? What else do you have going on?" But the second set of doctors actually had the time to go through my notes, my hospital notes, everything, and not notice that, "Hey, you've had a miscarriage in the last year. Hey, you showed up at urgent care bleeding profusely with a cyst ruptured. We need to get you in now and figure out what's going on." It was those second two doctors. They're the ones that said, "We need the ultrasound immediately. We need to do the Pap smear immediately. We need to do all these tests immediately."*

*S2 05:25 Well, when I called in to-- on the ultrasound schedule, because it was put in as an emergency ultrasound, they told me it was going to be three to six weeks for me to get seen. Okay, fine. Let's get it in. We got in, I think, four weeks after the initial rupture of my cyst that we thought was a miscarriage, but luckily, that one wasn't a miscarriage. That one was an actual cyst that ruptured. It just felt like I was going into labor. The doctor called me back the next day, the new doctor. My new OB called me back the next day and said, "This is unacceptable. I cannot believe they're making you wait four weeks to get seen. We need to have you go in now." Later that afternoon, I got a phone call from the ultrasound department because there's only two on the east side that I can go to. Because of [healthcare institution], we're limited to where we can go. She was able to get me in the next day. Because of her due diligence, because of her actually listening to me and taking the time to read over my notes, she understood that this wasn't just me having cramps, and the weight gain wasn't just over a period of time. This was a rapid weight gain.*

*S2 06:38 Everything else about me was healthy, but the rapid weight gain, the fatigue, all of my symptoms were like, "No. Something else is going on. I honestly think it is your IUD. We need to have that ultrasound to figure out what's going on." If it wasn't for her, I would've never had the ultrasound done. And then still to this day, two and a half years later, still battling the cysts. So, for me, it's one of those things like-- and, of course, that doctor ended leaving on maternity leave and never came back. So now I have a new doctor that's just like my first doctor, and it's like, "Oh, well, it's not a big deal. You are in your mid-30s. You're healthy. You're overweight, but, yeah, you're healthy." I'm like, "No. There's something going on. I know my body. There's clearly something going on. It shows on the ultrasound." But the new doctor is just as bad as the first one. The second doctor was fantastic, absolutely fantastic. I'm sad she's gone. She is the first one to actually truly listen to what my concerns were. She was talking like a friend, not like a professional.*

*S1 07:53 Okay. So that's really helpful. It sounds like listening to the concerns, but also, there being a follow-up with the urgency and action and it being timely as well. Does that sound like what--?*

*S2 08:05 Yes.*

Excerpt - Document: 106 Respect Interview-cleaned.docx, Position: 1624-3117

*are there any maybe specific actions that a medical provider or the nurses or doctors kind of performed, for lack of a better word, that really exemplified respect?*

*S2 04:46 Yeah. For one knowing my case. It was really hard for me when I came in and someone was scribbling on a piece of scrap paper [laughter] and didn't seem to know what was really going on with me. And I'm like, "Oh, my God." It made this appointment-- you should have read up a little bit before you came into the room. So that kind of stuff would be frustrating. And then in the healthcare system I was in, they kind of just scheduled you with whoever was available that day, so I felt like that made it hard too. I didn't have a consistent person that I saw other than the doctor who did my ultrasounds. But he deliberately made the choice to be like, "I'm going to do her ultrasounds. I don't want anyone else doing them." I didn't know at the time that you can do things like that. I just thought you had to go with whoever they gave you. So it was really nice having a consistent person who saw you. And then if someone didn't know you, that they followed up and-- follow-up was huge too. Feeling like, "Okay, this person is going to call me about the results," or they want me to come back in two months, or whatever, to make sure I'm not having these symptoms again. It was more like I had to make that call, so that was just difficult for me to navigate and feel that people really cared about what was happening.*

Excerpt - Document: 117 Respect Interview-cleaned.docx, Position: 16140-16939

*And I’m curious with medical care, for example, you said it was a five for how much you feel respected and a three for how much you trust it. I’m just curious what you see as a difference.*

*S2 29:31 Yeah. There was one incident, not incident which my surgical doctor could not do the surgery that day so they have a substitute or a different person perform or do my surgery. And my doctor told him how to go in and replace my shunt. But the guy wanted to go from a different spot which he went in, then it was kind of hard to replace my shunt the way he went in. Then so he went back to what my original doctor told him to do to replace my shunt. And the result of that him going in, the first time he went in have my left leg numb for three years. I mean, I trust medical care but after that-- yeah.*

**Convenience/ accessibility**: How easy or hard it is to schedule appointments or otherwise interact with healthcare system

Excerpt - Document: 127 Respect Interview - SP English translation - cleaned.docx, Position: 3161-3962

*So since you don’t have examples, what about if you could imagine for yourself something that would make you feel disrespected or that your friends or family members have been disrespected? If you can, think of examples. Or something that just wouldn’t make you feel respected.*

*S2 04:10 Yes. One example is that my [family member] had this pain and he went to the clinic and they told him that they were going to run some tests on him and, well, they never called him to-- they were going to call him to make an appointment in another clinic and they never called to schedule it. He called them and they told him to wait because they had said they would call him, but they never did. Those are things that don’t--*

*S1 04:32 Yes. Right, that --*

*S2 04:34 --that wouldn’t be good. But that’s not the case.*

Excerpt - Document: 127 Respect Interview - SP English translation - cleaned.docx, Position: 5000-5787

*Okay, so what can you tell me about the policies that a medical institution might have in place? For example, as I mentioned to you previously, what types of policies would demonstrate a lack of respect? Let’s say, something that the institution or clinic might do or not do that would demonstrate a lack of respect.*

*S2 06:23 Like, maybe, a lack of respect would be to not help other people who come from countries where neither Spanish nor English is spoken, because we’ve seen a lot of people who come here, for example, from Guatemala where there are other languages and no, I mean, it’s very difficult to obtain medical attention for them.*

*S1 06:52 Uh-huh. Okay.*

*S2 06:53 And it would be a lack of respect to look down on someone else or give them a dirty look. Yes, that [laughter].*

Excerpt - Document: 153 Respect Interview - SP English translation - cleaned.docx, Position: 1370-1820

*Could you give me some examples of the things that make you feel respected in your medical care?*

*S2 03:27 For them to respect the individuality of the patient. I like that. For them to, since I don’t speak English, bring me a translator. Almost always in [clinic], it’s almost always in person, never by phone call, and that kind of makes me feel more sure about what I’m understanding and of what I’m trying to say. That has been really good for me.*

**Inclusivity**: Recognizing and acknowledging diversity (racial, ethnic, cultural, socioeconomic, etc.) or failing to do so, including displaying cultural humility, avoiding imposing personal views, and implicit/explicit bias or discrimination (e.g., labeling, stigmatization, disparate treatment)

Excerpt - Document: 127 Respect Interview - SP English translation - cleaned.docx, Position: 706-954

*could you give us some examples of the things that make you feel respected when you receive medical attention?*

*S2 00:16 Yes, well, since I speak very little English, I like to be-- they find me a translator and everything; that makes me feel good.*

Excerpt - Document: 127 Respect Interview - SP English translation - cleaned.docx, Position: 1174-1582

*And so can you think of other examples? That maybe specific people have done something to make you feel respected.*

*S2 01:06 Well, yes. I mean, the same as I mentioned, right? They make one feel comfortable, trying not to make them wait longer, speaking-- even if they don’t speak English to me, by trying to speak a little Spanish or if not, by finding a translator. But yeah, one feels that. I feel pleased.*

Excerpt - Document: 127 Respect Interview - SP English translation - cleaned.docx, Position: 2387-2515

*Where there other moments you can tell me about?*

*S2 02:41 Well, well, this in particular is all that I like. Mm-hmm. I mean, no.*

Excerpt - Document: 127 Respect Interview - SP English translation - cleaned.docx, Position: 2860-3133

*Have you ever felt that you weren’t duly respected in the medical environment because of, for example, who you are, your looks, your beliefs or something like that or have you always felt respected?*

*S2 03:44 Yes, always. I always feel that yes, that they have respected me.*

Excerpt - Document: 127 Respect Interview - SP English translation - cleaned.docx, Position: 5000-5787

*Okay, so what can you tell me about the policies that a medical institution might have in place? For example, as I mentioned to you previously, what types of policies would demonstrate a lack of respect? Let’s say, something that the institution or clinic might do or not do that would demonstrate a lack of respect.*

*S2 06:23 Like, maybe, a lack of respect would be to not help other people who come from countries where neither Spanish nor English is spoken, because we’ve seen a lot of people who come here, for example, from Guatemala where there are other languages and no, I mean, it’s very difficult to obtain medical attention for them.*

*S1 06:52 Uh-huh. Okay.*

*S2 06:53 And it would be a lack of respect to look down on someone else or give them a dirty look. Yes, that [laughter].*

Excerpt - Document: 114 Respect Interview-cleaned.docx, Position: 326-1715

*So could you give me some examples of the kinds of things that make you feel respected in your clinical care?*

*S2 02:23 Often time, I find one of the biggest things is to be listened to. I’ve had situations in the past where my questions weren’t answered or they didn’t focus on the one thing that I was trying to focus on and focused on something else entirely that I didn’t really want to talk about at that time. So for instance such as I’m talking about I guess this is more of a [inaudible] because this wasn’t specifically happening to me. But talking about, I would like to-- my brain stopped functioning. I’m sorry.*

*S1 03:11 No take all your time you need.*

*S2 03:13 Basically, I sprained my ankle but then you go in there and someone will say, “Well, you need to lose weight.” That kind of thing. And though I understand it’s like-- I understand, “Yes, I’m overweight. Yes. I know I need to lose weight. I have talked about this before,” but people tell when it gets brought up and shoved on my face again when that’s not the reason that I’m even there. It comes off as extremely disrespectful and demoralizing, I guess. I think sticking to the topic, maybe perhaps trying to find what the actual cause of specific injury or bruising, where there is-- well, it should be done and not focusing on things that aren’t necessary at that point. And I’m trying to think what else might--*

Excerpt - Document: 114 Respect Interview-cleaned.docx, Position: 3153-3829

*Can you think of any ways that a medical institution could have, any sort of policies that a medical institution might have that would show respect of show lack of respect?*

*S2 07:01 Well, that’s a good question. I guess, it sort of depends on what aspects we’re talking about. Personally, I’m not entirely sure but in thinking about myself, my family, and things that I’ve seen happen with friends and family in medical care. Being sure and having policies I suppose to make sure that if someone is-- if a doctor is able to keep his or her moral standpoint out of his medical practice. It’s especially related to youth. I don’t believe they have any place in medical practice.*

Excerpt - Document: 114 Respect Interview-cleaned.docx, Position: 3839-4495

*Can you think of examples of how that make them up?*

*S2 08:14 The LGBT community is very often persecuted and made to feel very different or wrong. My sister is a transwoman and so I’m very glad that she hasn’t had at least an issue that I’m aware of. But I know many other people have in which they’re being misgendered and basically people are-- there's no reason to misgender someone. I understand in medical practice, there is a necessity to understand the biological aspect of them but there’s still a matter of respect in that they should not be made to feel uncomfortable. I was going to [inaudible] this as well I’m trying to remember what was that.*

Excerpt - Document: 114 Respect Interview-cleaned.docx, Position: 4512-4911

*o that’s a great example. I guess I’m trying to bring it back to that what an institution can do like maybe what kind of policies do you think would address that?*

*S2 09:42 Well, that was my idea in that there should be policies that make sure that LGBT community feels safe and that they’re able to be safe within an institution. Because if you can’t trust your medical providers, who can you trust.*

Excerpt - Document: 114 Respect Interview-cleaned.docx, Position: 11458-12639

*And sometimes people say that their experiences in the medical setting effect whether they want to join research studies, was that true for you at all?*

*S2 19:25 Actually, I’d have to yes but in a positive way. Since I’ve started going to [healthcare institution], most of my experiences have been positive. I’ve had a few times where I didn’t feel terribly listened to and few of my questions were answered as much as I would have liked them to have been. But I went into [healthcare institution]. I had to have gallbladder surgery and I had to have it removed in emergency surgery. And I went in there terrified and because how everything went with the surgery, with recovery, I felt actually extremely comfortable and I’m no longer afraid of surgery. So I think almost everyone I’ve met has had a very good to me there. And I think I like [healthcare institution] most because of the fact they have so many inclusive programs to what not?*

*S1 20:36 So between those good experiences you had and the fact that they are an inclusive institution that made you-- it sounds like those made you more interested in joining the research study. Am I getting that right?*

*S2 20:51 Yes. Yes.*

Excerpt - Document: 101 Respect Interview-cleaned.docx, Position: 2498-3444

*And then, so what about the policies that the medical institution has in place? What kind of policies do you think show respect?*

*S2 06:03 I mean, I think now-- I mean, recently, I had [surgery], and I didn’t have insurance. So with them being able to actually still go in and do what they needed to do and not treat me, yeah, like a leper, pretty much, because I didn’t have insurance and still do everything that they would’ve normally done, that was pretty great. I was pretty freaked out when I had to go in and didn’t know what was going to happen since I didn’t have insurance at the time. So that policy of being able to still take care of the patient and take care of what they needed to do. And then I had people come in and help me get Medicaid set up and everything, so it was all taken care of anyway. And I had to stay for four days, so their staff treated me amazingly, made sure everything was taken care of with me and my wife, so.*

Excerpt - Document: 101 Respect Interview-cleaned.docx, Position: 4181-5608

*So could you give me some examples of the sort of things that might make you feel a lack of respect in your medical care?*

*S2 08:26 Pretty much just health insurance [laughter]. That’s about the main one that kind of kills me. I mean, I don’t have any problem with any of the doctors. It’s just cost. Years ago, I almost cut off my thumb on a table saw, and it was a big question of whether I wanted to go in and pay $600 to get it stitched up or if it would heal on its own.*

*S1 08:54 Yeah, okay. So that health insurance aspect is kind of the big factor when it comes to a lack of respect and what--*

*S2 09:11 Yeah. I mean, it’s not really respect for the health insurance-- I mean, not really respect for the health industry. It’s more a question if it’s worth me dealing with the pain or not, and it’s just not wanting to go in and having to figure out how to pay for it.*

*S1 09:31 Okay. So would you--?*

*S2 09:32 So, I mean, I make just enough money to where I can’t afford [inaudible] health insurance, but I make too much where I can’t be on Medicaid. So it’s kind of one of those where I’m kind of in the middle there where it makes it really tough.*

*S1 09:57 Yeah, yeah. So that seems like it’s-- it kind of sounds like it’s one of those policy issues where perhaps it’s a lack of respect for certain people who kind of fall in between what they can afford and what Medicaid is set up for. Is that right?*

*S2 10:18 Yeah, yep.*

Excerpt - Document: 101 Respect Interview-cleaned.docx, Position: 6830-7004

*And have you ever felt a lack of respect in the medical setting based on who you are or how you look or what you believe?*

*S2 12:08 No. No. Honestly, I haven’t ever seen that.*

Excerpt - Document: 150 Respect Interview-cleaned.docx, Position: 2775-2923

*All right. And have you ever felt a lack of respect in the medical setting based on who you are or how you look or what you believe?*

*S2 06:57 No.*

Excerpt - Document: 150 Respect Interview-cleaned.docx, Position: 2939-3451

*And thinking a little bit more, say, about your friends or your family, is there anything that you can imagine that might make-- or that they might perceive as a lack of respect that you've seen before in a medical setting?*

*S2 07:24 Yeah. I guess like your financial status, I've heard of before.*

*S1 07:37 Sorry, was that questioning someone's financial status, or--?*

*S2 07:41 No, just assuming that if you don't have a lot of money, then you probably aren't doing XYZ, when that's not necessarily the case.*

Excerpt - Document: 154 Respect Interview-cleaned.docx, Position: 3337-4997

*And have you ever felt a lack of respect in the medical setting based on who you are or how you look or what you believe?*

*S2 07:08 If it was, it never, to me, connected that that was the reason why. I've been reading more stuff, and more things are coming out, and I've read articles on stuff. I'm like, "Huh, huh." But, yeah, I never walked away thinking, "Oh, they were dismissive." Well, no, no, wait, I take that back. Yes, there have been interactions where it was like this idiot man doctor - excuse me - done some things that-- I know my body as a woman. There have been a couple of interactions like that, yes.*

*[silence]*

*S1 08:03 All right. And if you're okay with it, would you mind kind of talking to me about that experience?*

*S2 08:14 Okay. Hold on. Let me blow my nose.*

*S1 08:16 Oh, sure. Yeah, no problem.*

*S2 08:19 I've just gone into the office here. Years ago-- I mean, this is TMI too, but I didn't have any health insurance, so my mother called the advising nurse on her health insurance and asked if they would speak to me because I was bleeding anally quite a lot. I've had digestive issues off and on for years. But I spoke to a doctor on the phone, and he kept saying, "Are you sure you're not on your period? You're probably just on your period." And I'm like, "I know the difference between my vagina and my anus." I'm like in my mid-20s at this point. I think I know the difference. So, yeah, it still makes me mad when I think about it because I'm just like, "You're an idiot. Listen to what I'm saying."*

*S1 09:11 Yeah, no. So that's really one of those unfortunately terrible examples of doctors just not listening to patients. Yeah.*

Excerpt - Document: 172 Respect Interview - SP English translation - cleaned.docx, Position: 2545-3075

*have you ever felt that you weren’t respected in a medical environment because of who you are? For example, your appearance, your beliefs, your language, your education, your race, or things like that.*

*S2 05:13 No.*

*S1 05:13 No? Okay. Now, what about if you could imagine something that would make you feel like people didn’t respect you?*

*S2 05:23 That sometimes simply one’s race, when they look at someone and they are Hispanic, and sometimes they do seek to treat those people badly or they don’t want to treat them the same.*

Excerpt - Document: 132 Respect Interview-cleaned.docx, Position: 2791-4830

*What about policies that a medical institution has in place? Can you think of any policies that would show respect or show disrespect, by chance?*

*S2 06:14 Well, I know that a lot of offices and people in general are trying to be more inclusive and make sure like, “Okay. We’re for everybody, not just a certain group of people.” I feel like that’s important. Because there have been times in the past where I didn’t have health insurance. And I had an injury. And they just said, “Well, stay off of it.” Stay off of it, that’s all they told me. And quite a few years later, I had insurance at some point. I went in and got my knee checked for that injury for that [inaudible]. And they just said, “Oh, you have an ACL tear here that was quite old. And it’s so old that there’s nothing we can do about it. Because it’s just completely gone now.” And I knew at that moment that that injury I had was an ACL tear that could have been repaired. But because I didn’t have health insurance, they didn’t take the time to look at me. They just brushed me off. So I feel like-- I think I’m tying up two different things here. It’s getting confusing in my own head. To not be excluded because of what you look like or what race you are, stuff like that, those things, I find those respectful. And I appreciate those. I am a Caucasian female. So basically, I haven’t experienced some of that. But there are other people who have. But then, also, I think policies that say you deserve to be treated regardless of whether you have the money to pay for it or not-- and I feel like that that needs be more-- it’s stated, yet not always followed. Does that make sense?*

*S1 08:13 Yeah, yeah. Okay.*

*S2 08:15 So I guess it’s an unspoken thing that says, “We’re going to treat people who we know can pay rather than those who don’t.” That’s a disrespectful policy because every life has value. And if somebody can’t afford to pay for it does not mean that they shouldn’t have a chance to live or have a knee that’s not totally whacked out the rest of her life.*

Excerpt - Document: 110 Respect Interview_cleaned.docx, Position: 2098-2918

*can you give me some examples of the sorts of things that might make you feel a lack of respect from your medical care?*

*S2 05:49 So [laughter], excuse me, I'm sorry. I have a genetic condition that can present in many different ways in things that happen, and if [laughter] my doctor isn't very knowledgeable about the condition that I was born with, they kind of brush off my symptoms as "Well, it doesn't exist," or, "It doesn't matter." Because it's one of those things that anything could happen at any time. And it's like, "Well, you've never had that before, so why would that be a problem now?" So it's not really being heard from a point of view of "We know that you have this. We know that this can be associated with it," but they don't understand that and that's where it [laughter] can get very frustrating.*

Excerpt - Document: 110 Respect Interview_cleaned.docx, Position: 2941-3078

*And have you ever felt a lack of respect in the medical setting based on who you are, or how you look, or what you believe?*

*S2 07:11 No.*

Excerpt - Document: 110 Respect Interview_cleaned.docx, Position: 3128-3463

*are there any policies that medical institutions or any other medical staff can do that would be a sign of a lack of respect?*

*S2 07:38 I guess, not treating you because of your beliefs, or family values, or backgrounds. Any of that, I guess.*

*S1 07:58 Okay.*

*S2 08:01 Whether you can afford it or not, for treatment?*

*S1 08:03 Okay.*

Excerpt - Document: 161 Respect Interview-cleaned.docx, Position: 6883-13643

*what I'd like to do is kind of take that broader question of respect and kind of flip it around. So could you give me some examples of the sorts of things that might make you feel a lack of respect in medical care?*

*S2 11:50 Well, not so much a lack of respect but kind of more like the [inaudible]. I think, for example-- and I don't know. Well, I think it's really different because we're talking about respect. I've seen situations where it's gotten out of control, and it's been very disrespectful, and it's kind of interesting. It just depends on the patients as well. But it's been really disrespectful. A lot of times, and it does happen in hospitals where patients are really disrespectful to many staff. So that's really wow. And you try to help them out as much as you can, but it comes to those situations where they're really, really disrespectful. Either they say bad words. Either they don't follow through with their [indications?] some of their medical staff is recommending to them. I don't think they [inaudible].*

*S2 13:03 What the medical staff want is to get the patient well. They want the patient to be well, so they do a lot of recommendations, right? So if the patient doesn't follow through and things like that or someone has to call, then a lot of the patients get really angry. And do you expect them to listen to anyone? No. That occurs. So it's like, "Wow." [inaudible] but getting that good care. And it's the opposite of, yeah, the patient being good, just respectful to the staff. And I don't know if this could be the situation, I guess, that patients go through. But yeah, I've seen that opposite side. And then the last time I got disrespected [inaudible]. I think we do try our best to have our patients be content and have the care that they need and hear them out and get them waiting to be-- I mean, the patients come first. They really need to take care of them. But yeah, of course, you get what's in the [inaudible] and out there. So I've seen them too. But yeah, I am speaking more towards not the patient's part of it, but I think it's more of a [tangent?] [inaudible]. I think I'm getting out of that spot.*

*S2 14:49 Insurances, it's actually like insurances. When patients come in and their insurance is not contracted with the hospital, and they'd say, "Oh, my goodness," it's really hard. And especially when you don't have no insurance at all, and they get referred to the department of enrollment services where they do get the help. Actually, hospitals do have enrollment services departments where they help patients get coverage, whether it be a copay or a minimal copay. That's something just to cover the medical expenses. And that is really-- well, it's really interesting to see when a patient doesn't qualify for medical insurance or help because they're over budget or they're over-income. That really interests me because patients, they're in the middle. They get stuck in the middle where they don't have insurance. They can't go to their doctor's appointment because they don't have insurance. And of course, if they go, they get bills with this huge amount of money. So that's something that's really interesting, and that's really hard to see, too, because you do want to help them out. You do want to get them the care that they need. But because of that insurance that is blocking their way to care, that's really messed up. But then again policies and procedures, and it's like, "Oh, my God." I've seen a situation where-- and I have to see that because we try so much to help them out. A patient had to leave her job just for her to get coverage.*

*S1 16:40 Oh, wow.*

*S2 16:40 That is really messed up because her job is supposed to be offering her some sort of insurance or coverage if she's working 40 hours. If she's a full-time employee, then she should be getting a coverage or some sort of benefit from her job. Oh, my God, I was in shock when I heard that. And she wasn't able to get the care that she needed because of it. She wasn't able to qualify for anything because she was over-income. And it's not even that much because there are brackets from where patients qualify for the discount programs or Medicaid or things like that. There's procedures or brackets that patients need to have or the amount of money that they need to earn kind of thing. And I don't know how they calculate that, but I've heard about it. And when they're over that bracket, or they don't quality within that bracket, they don't qualify for anything. Oh, my God, so that patient was in that situation where she had to leave her job just because of it. And then after she left her job, she qualified for the discount program, more through [inaudible]. There should be more help in that sort of area of insurances for patients that are uninsured that they would have the help or the resources to get insurances like a normal person that works full time that has coverage from their own job. But we do have patients that don't get coverage. They don’t have insurance from their job. They have to rely on the discount programs or Medicaid to help them. And that's most of that Medicaid is-- but we have Medicaid, and we have discount program. But we have the separate part of this where uninsured patients that are underserved because that really helps them out.*

*S2 18:36 But then there are some situations that they could qualify, and that's been messed up. That's been pretty messed up quite a bit. When they qualify for them, it doesn't work. We tried so much to help them out. But then once that happens, there are situations where-- and I've also seen grants, like the grants the hospitals offer for patients. For example, breast cancer, there's grants in the hospital that come from [inaudible] or other foundations, nonprofit foundations that come in and help the patients out with their copays, which really awesome. Those organizations are really helpful for the hospitals to have because they help those patients get the care [inaudible] or schools and even follow-up appointments with the oncologist. That's really awesome. But then it really sucks because when the funds run out, then the patient is left with-- there's no funds anymore. You're going to have to cover your tests, the visit. And it's like, "Great." They have to go through the process again of going through enrollment services and getting coverage with the discount program," and that is really wow. So I've seen lots and lots of the insurance part of what patients go through just to get that coverage, just to get their medical care. And it's kind of something that I wish our government could do a bit more on and be more aware. But then, again, that's tangent to the whole thing, but I thought that was something important, so.*

Excerpt - Document: 136 Respect Interview-cleaned.docx, Position: 5312-5618

*Have you ever felt a lack of respect in the medical setting based on who you are or what you look like or what you believe? Do you ever feel like that's happened to you?*

*S2 02:29 No. No, on the contrary. Everybody has always been very respectful and kind and answered all of my questions [inaudible] to me.*

Excerpt - Document: 153 Respect Interview - SP English translation - cleaned.docx, Position: 1370-1820

*Could you give me some examples of the things that make you feel respected in your medical care?*

*S2 03:27 For them to respect the individuality of the patient. I like that. For them to, since I don’t speak English, bring me a translator. Almost always in [clinic], it’s almost always in person, never by phone call, and that kind of makes me feel more sure about what I’m understanding and of what I’m trying to say. That has been really good for me.*

Excerpt - Document: 153 Respect Interview - SP English translation - cleaned.docx, Position: 1887-2734

*Do you think you can tell me something about the providers? Something that they maybe do specifically, or maybe even the receptionist? Something that they do in particular or something that one person has done with specific action to make you feel respected?*

*S2 04:25 Yes. They always ask me before touching me or telling me something if I understand, if they can do it. My [family member] always accompanies me to the consultations and they don’t realize that he’s my [family member]. They always ask me who the person is at my side. Everyone is good, good; everyone has been very respectful and friendly towards me. I don’t have any bad experiences from anything that has happened to me, no. Even the translators, when the doctors are going to examine me, they turn their backs so as not to see where the doctors are touching me or examining me.*

Excerpt - Document: 153 Respect Interview - SP English translation - cleaned.docx, Position: 4248-4684

*Could you imagine something that would make you feel like you aren’t respected?*

*S2 07:24 For example, for them not to respect me, they would be rude to me, they might treat me badly for being a Hispanic woman, or for not speaking English. What else can I say? I don’t know. I think those would be the examples, but they haven’t happened to me. I wouldn’t know how to explain it well, but I think it would be something along those lines.*

Excerpt - Document: 153 Respect Interview - SP English translation - cleaned.docx, Position: 5157-5953

*just want to know, do you still remember when they invited you to participate in the study?*

*S2 08:35 Yes, of course I do. The oncologist invited me. She asked me if I had had a genetic study done, which I had tried to get done for myself, but they hadn’t let me because the insurance that I had didn’t cover it at the place where I was sent. So they had already let a lot of time pass and hadn’t run the study on me. When the doctor who was in the study invited me, asking me if I wanted to have it done, and that it was at the clinic, she told me all about and that it would be very simple for me, that it was a saliva sample and some questionnaires. So right there at the clinic they helped me so I could do it. And yes, it really did seem very simple to me and everything very well explained.*

Excerpt - Document: 153 Respect Interview - SP English translation - cleaned.docx, Position: 17721-18179

*Okay, and now, could you tell me a little more about why you responded to these questions about respect in this way?*

*S2 01:30 Yes. Well, because I’ve felt respected. I’ve felt that they take their time with people, at least in my case; that they’ve always treated me well. They speak to me in my language, which is Spanish, and for that I thank them very much. And, well, they’ve always based our interactions having good behavior and by speaking well to me.*

Excerpt - Document: 107 Respect Interview-cleaned.docx, Position: 2390-3576

*And it's okay if you don't have a specific example, but sometimes people say they-- sometimes people a lack of respect based on-- if they're treated differently based on who they are or how they look, or what they believe. Have you had any experiences like that?*

*S2 08:39 Can you say that again?*

*S1 08:41 Yeah. Have you ever felt a lack of respect based on who you are or how you look, what you believe, and anything? What language you speak, what your culture is, anything like that?*

*S2 08:55 I don't think so.*

*S1 08:56 Okay. Could you imagine anything-- it sounds like you don't have any specific examples of feeling a lack of respect in medical care, which is good. I'm glad to hear that. Can you imagine anything that might make you feel a lack of respect?*

*[silence]*

*S2 09:33 I guess [inaudible] just assume stuff about me.*

*S1 09:37 Yeah, and what kind of-- oh, go ahead.*

*S2 09:41 I guess assuming if I speak another language or something, or-- basically just assume who I am and not really ask, I guess.*

*S1 10:14 Okay. Yeah, and that sounds like it goes back to what you were saying earlier about them not really listening to what decisions you're making.*

*S2 10:24 Okay.*

Excerpt - Document: 123 Respect Interview-cleaned.docx, Position: 2212-4099

*And I guess I should ask can you think of any particular examples when you’ve really felt like you’re being really well respected?*

*S2 04:53 Yeah. So I was not well very much. I’ve had chronic stomach problems for over 20 years and I’m overweight but I’m not significantly overweight. I’m not like morbidly overweight. And so throughout the years, I got a new complaint with the stomach illness and I’ve had all sorts of weird experiences where people have drawn mice and some mice are skinny and some are fat. Last time, I was compared to fat donut which was really a weird thing. But the positive experience is that I was going to have maybe a stomach repair surgery and I went to see the surgeon. And he actually listened to all of my complaints and said, “Oh, okay. That makes sense. You’re a little overweight but you’re not that overweight and your symptomology doesn’t mean what you’re saying and then if I do this surgery without really checking into it, it can cause you a lot of harm.” He explained all the procedure to me, what that could look like and then he said I’m going to do some extra testing because I want to make sure. And then, knowing the cause because of the test and he referred me to-- they actually found the stomach illness that I’ve probably have had over 20 years, and it was because he actually listened to me and it wasn’t just like default, “Oh, you have to shed your pounds. This is severe.” Or this is like, “Hey, this doesn’t kind of match up.” I’m like, “Yeah. That’s it. That’s what I’ve been saying.” And so just that-- so his name was Dr. [name]. His attention to detail and really like, “What?” where he was just like curious about it too. I mean, it’s been a little bit of life-changing for me.*

*S1 06:33 Yeah. So it sounds like it wasn’t just a matter of showing respect but it actually had a clinical impact on your care.*

*S2 06:42 Yeah. It did.*

Excerpt - Document: 123 Respect Interview-cleaned.docx, Position: 4119-8710

*you sort of alluded to this but can you give some examples of times when you felt a lack of respect in medical care?*

*S2 06:52 Yeah. So on my journey with stomach stuff, when I was going in-- I mean, I was throwing up in my sleep and so I went into the examination room. So we don’t know if they’re primary care and the woman I saw, I said, “I’m throwing up almost twice a week in sleeping mode.” And she’s like, “Well, you only have two episodes. That’s good.” I’m like, “I don’t think that’s true.” You know, I get it. I’m a [occupation], I’m not a doctor but that doesn’t make sense because you can die when you’re hearing that phrase, that’s disrespect. And so she was just like, “No. Two episodes is good.” And I asked to talk to a specialist and she said I didn’t need to see a specialist. So she really didn’t validate my feelings nor did she wish my medical desire which I felt was ridiculous. And then, I went back. A week later, once again I was throwing up in my sleep. I went in and saw-- I don’t know if he was a nurse practitioner or something, and he was just like, “What? Some people are thin donuts and some people are fat donuts.” And he actually compared me to like a glazed, sprinkled Mustang donut and was saying that my digestive system is like the center of that donut and if I were just as more compact donut that was more fit, my food would just go through me. But since I was more spread out like that fat sprinkled donut, then I was going to continue to have problems, and I was just more shattered. Then, I said, “You know, as I’ve gone through this journey and I extraordinarily lost a bunch of weight and I’m still working out.” And then I got shin splint. I tore my rotator cuff. I’ve had a series of cortisone shots and all of these things, and I’m kind of afraid to jump back. And your family is like, “Well, a cortisone shot is better than not being fat.” And I think it could probably be two ways.*

*S2 08:50 So again, he was just so quick to jump on the weight bandwagon. He wasn’t listening to my symptomology at all, and he was just like, “Well, you know.” And then, this was like minimize shame and it seemed ridiculous to me also like is that really the best solution our medical providers can give us. It’s like, “Well, you’re obviously in some pain. Just go get another cortisone shot” which is weird because there’s research about cortisone shots that you’re only supposed to have so many and he was just dismissive of it. But then I had a really bad example where I had had a bad pedicure but not a chronic issue. But I had a bad pedicure and I had gotten an ingrown toenail. So I went in and I was telling the provider. They’re like, “Oh, just soak it.” So I did and it didn’t resolve. And so it ended up being where I had to have it removed. And I don’t what he was like a physician’s assistant or something. He had a student come in and said, “Do you mind the assistance?” I’m like, “You know, I really kind of prefer, no offense to you.” Whilst she’s trying to learn and now what has happened is-- so I felt pressured. He said, “She’s a very nice old student.” So I’m like, “Okay. She can assist.” They didn’t know my foot. My foot ended up being-- I don’t know. Well, at least 12 shots in my toe, so the toe ended up being bruised for a week. They removed half of my toenail. And because students used like a little bottlebrush with the root. They killed the toenail root. My toenail now can only grow about a quarter inch out and then it dies because they’ve permanently damaged my root. So I don’t know. Well, it wasn’t respectful. I didn’t want the student. They probably shouldn’t ask if a student is right there because it puts the patient in a precarious spot. When I said no, he didn’t accept no, “Oh, common. She’s just trying to learn; I’ll be here.” And then, she failed with that and now I have a permanent like-- does it kill me? No.*

*S2 10:52 But I’ve had to have that toenail removed a couple of times since then. I never had ingrown toenails before. It wasn’t like I had a chronic problem where I needed that level of treatment. And now, it literally dies off and it turns like almost a bluish color. It’s unsightly that doesn’t kill me but had he just listened to me in the first place perhaps I wouldn’t have this and I actually asked to see a specialist for it. And my providers said I didn’t need to.*

*S1 11:25 Yikes. Well, I’m sorry to hear you have so many examples of-- that sounds rough so I’m sorry you had to go through that.*

*S2 11:35 Like if I’m old, over 45 years, this wouldn’t have been bad, but I’m [in my early 30s].*

Excerpt - Document: 123 Respect Interview-cleaned.docx, Position: 9917-10618

*And some of your examples earlier might have spoken of this but I wanted to ask explicitly have you ever felt a lack of respect in the medical setting based on who you are, what you look like, or what you believe or anything like that?*

*S2 13:11 No. I mean, I think that I sometimes get-- no. I mean, I’m a Caucasian female and educated so for the most part, I never feel like that. I think sometimes people default to if you’re overweight and that’s the experience of my husband and, I mean, my sister as well but I don’t think it’s discriminatory. I think they just have to-- my guess is that it’s a stereotype. There’s this stereotype around heavy people and I have definitely been a victim of that.*

Excerpt - Document: 142 Respect Interview- cleaned.docx, Position: 5528-7631

*Have you ever felt a lack of respect in the medical setting based on who you are or how you look or what you believe?*

*S2 10:21 No. There's not a time that I can think of specifically. So I would say no to that.*

*S1 10:33 Okay. Can you imagine anything like that, that might make you feel a lack of respect?*

*S2 10:41 Yeah, I--*

*S1 10:44 Oh, go ahead.*

*S2 10:45 Yeah. I was going to say, I feel like just everyone being culturally different and religiously-- I mean, there's so much going on in the world too, so I can definitely imagine what some people might go through. I can't say I've witnessed any myself. But yeah, I can imagine some discrimination going on within the medical care.*

*S1 11:18 And so what about that? What about that would make you feel like it was disrespectful?*

*S2 11:28 So, I actually listened to a podcast that had talked about this, how the care varies from person to person based on their background, having similar cultural or religious beliefs.*

*S1 11:49 That's so funny. I think we listened to the same podcast [laughter].*

*S2 11:54 So, I mean, to answer your question, I feel like it is showing that people, they feel more relatable and respected when they have someone with similar beliefs. They feel like there are some differences when people are cared by medical professionals who don't have a similar belief system [laughter].*

*S1 12:16 And so how do you think that's tied to respect? The beliefs or the culture?*

*S2 12:23 Yeah. So I'm kind of relating to lots of podcasts that I just listened to. But it was interesting that people-- it was said that some people might find that racist. Or some people may find it as more the relatability of having care by someone who has a similar background as them. And so I think it's important to acknowledge that. Hopefully, most people in the medical field do want to provide the care for patients. But I think, just having that respect and having that relationship can make a big impact on their health.*

*S1 13:04 So kind of really knowing your patient and whether that be on a cultural and belief level is--*

*S2 13:13 Right. Exactly.*

Excerpt - Document: 149 Respect Interview - SP English Translation - cleaned.docx, Position: 6025-6494

*And, at any time have you felt that they didn’t respect you as in the tone of the doctor toward you or your beliefs? For example, have you felt that you weren’t shown respect due to your race, ethnicity, language, things like that?*

*S2 08:42 Well, right now, I don’t remember. No. I haven’t seen that.*

*S1 08:47 Okay.*

*S2 08:49 Nevertheless, [inaudible] (she has been) being kind to you when you go to an appointment. Even the secretaries are kind. No. I haven’t had--*

Excerpt - Document: 151 Respect Interview - SP English translation - cleaned.docx, Position: 3261-3558

*How about, have you ever, by chance, felt that you were not respected within the medical environment, because of who you are? For example, due to your appearance, your beliefs, your race, your ethnicity, your language or education or things like that.*

*S2 06:38 No, very good attention is provided.*

Excerpt - Document: 151 Respect Interview - SP English translation - cleaned.docx, Position: 3568-4094

*So, could you imagine something that would make you feel that you are disrespected? Or maybe you’ve been told that by one of your friends or your family. Can you think or imagine an example? If you can.*

*S2 07:06 I think that they lack respect if they don't pay attention to us, if they can't speak to us in our language, if they don't give us immediate help. I think that may be disrespectful, but it didn't happen to me, and I didn't / (hadn’t) realize(d) that (this had happened to me) they had [inaudible] me. I don’t know.*

Excerpt - Document: 151 Respect Interview - SP English translation - cleaned.docx, Position: 4105-4729

*That’s a good point that sometimes they aren’t showing you all the respect, because sometimes you don’t notice it. But you say that you haven’t noticed it, and you haven’t felt a lack of respect.*

*S2 07:47 No, no sir, they have taken great care of me. The whole process has been in my language. There’s been no obstacle for me to communicate with them or they with me, because they have the possibilities to have the interpreter. They speak in Spanish or immediately ask me, “Do you want English? Do you want Spanish? Do you want this to come out?” They are always with the patient, who is satisfied with the given treatment.*

Excerpt - Document: 129 Respect Interview-cleaned.docx, Position: 3679-3817

*And have you ever felt a lack of respect in the medical setting based on who you are or how you look or what you believe?*

*S2 07:38 No, no.*

Excerpt - Document: 108 Respect Interview-cleaned.docx, Position: 5753-6410

*have you ever felt the lack of respect in the medical setting based on who are you, how you look, what you believe, or anything like that?*

*S2 10:06 I mean, I guess like the vaccination thing is the belief or sometimes when I-- I do use a lot of plant medicine and energy medicine and that’s when I think sometimes that helps-- looked down upon in the medical community. But definitely, I mean, I’m just a regular white girl so I have not felt based on how I look or anything like that, that I’ve been discriminated against.*

*S1 10:49 I’m sorry. Did you say you have been discriminated against or you have not?*

*S2 10:53 No. No. I have not based on how I look.*

Excerpt - Document: 126 Respect Interview-cleaned.docx, Position: 5183-5957

*And have you ever felt a lack of respect in the medical setting based on who you are or how you look or what you believe?*

*S2 10:24 Yeah. I think so. I mean, I am pretty obviously a woman of color. I look Asian. I sometimes feel like I don’t have as much of a rapport with clinicians as I might have if I were white. Yeah. But that’s actually mostly been in [city 1], so I think that’s also maybe specific to culture, part of the country.*

*S1 10:55 Okay. And you mentioned that being specific to [city 1]. Have you had experiences in other cities or regions in the states that may be [crosstalk]?*

*S2 11:07 Oh, yeah. Yeah. I’m from [city 2], originally, and so it’s kind of a bit of a contrast in terms of how visible people of color are in a few places, and it’s pretty white.*

Excerpt - Document: 156 Respect Interview-cleaned.docx, Position: 3646-4196

*So have you ever felt a lack of respect in the medical setting based on who you are or how you look or what you believe?*

*S2 08:40 I used to smoke cigarettes, and I feel like that definitely changed some doctor's opinions on me, and I was treated differently after that. And then I feel like there were a couple of doctors [at some of the hospitals and clinics I visited] that were a little-- I don't know. I feel like their attitude changed when they found out that I was on state health insurance, which I thought was interesting, but those were it.*

Excerpt - Document: 158 Respect Interview - SP English translation - cleaned.docx, Position: 3642-4058

*And, how about, any time when you felt that you weren’t respected within a medical environment, because of who you are? For example, due to your beliefs or, for example, your race, your ethnicity, your gender, your language, your religion? Any of those things.*

*S2 07:04 No, never, not even with that. They have never been disrespectful due to religion or all of that. No, everything you mentioned to me, no, never.*

Excerpt - Document: 128 Respect Interview - SP English translation - cleaned.docx, Position: 1073-1831

*So could you give me some examples of the things that make you feel respected by your medical care?*

*S2 03:56 The people, you told me? Yes, correct?*

*S1 03:59 Yes. It could be about the people, or maybe about the clinic you go to. Basically, what are the things that make you feel respected?*

*S2 04:15 By making time for me, by listening to my needs; although it almost never happens, I like to be heard when I describe what I have come in for or what I need them to tend to all in my own language. The behavior of the people when they are attending to someone is at times absent; they aren’t there, they aren’t present, though maybe in their bodies, yes, but they aren’t present enough to be able to listen and be able to understand what someone really wants.*

Excerpt - Document: 128 Respect Interview - SP English translation - cleaned.docx, Position: 5441-5826

*I want to ask you, have you ever felt that you haven’t been respected in the medical environment due to who you are? For example, due to your looks, your beliefs or, for example, your language, or things like that.*

*S2 10:57 Yes. Sometimes I’ve felt the difference, when some doctors don’t distinguish between differences and others highlight them very clearly, and that’s very painful.*

Excerpt - Document: 128 Respect Interview - SP English translation - cleaned.docx, Position: 5847-6707

*Could you imagine something that would make you feel not - apart from what you’ve already told me, of course - that makes you feel that they have no respect for you? Maybe, for example, stories or narratives of what has happened to your friends or family members who might have gone through something similar in which they didn’t feel respected.*

*S2 11:46 Well, normally, some doctors do this, and maybe not all, and sometimes in my community people comment, “Yes, they do it to me, too,” but we also have encountered really nice commentary, like, “My doctor really is a good person.” But just as soon as we hear of a positive comment, we also hear that other type of commentary, including some people with terminal illnesses where they’re telling them, “No, you aren’t sick with this, and you don’t have such and such type of benefits, so we can’t attend you.”*

Excerpt - Document: 116 Respect Interview-cleaned.docx, Position: 2063-3190

*So now I want to kind of flip that larger question and ask you if you could give me some examples of the sorts of things that might make you feel a lack of respect from your medical care?*

*S2 05:56 I think that would be brushing off patient concerns, not really taking much time or thought into something they’re concerned about. I’m trying to think. I know one of my coworkers that I work with has had issues with her iron deficiency and such. And she’s tried to bring them up with her doctor and get some testing for it, when she found out one of the blood panels they did didn’t even search for the type of deficiency that she was curious about, but they just did that one and told her, no, she was fine, that sort of thing.*

*S1 06:41 I see. Okay. Huh.*

*S2 06:44 Yeah. Yeah, that sort of thing with the doctors and such not taking into consideration patient concerns. I’ve had other friends of mine that have had-- like they've been kind of dismissed of health concerns just because they’re overweight, like, ″You just need to lose weight and you’ll get better.″ Those comments were basically made. Yeah, stuff like that.*

Excerpt - Document: 146 Respect Interview- cleaned.docx, Position: 8606-9291

*Would you say that you've ever felt a lack of respect in the medical setting based on who you are or how you look or what you believe?*

*S2 12:38 I wouldn't think so, no. I mean, I know I'm overweight. I've never really had a doctor tell me, "Hey, you're obese. You need to do something about it," other than my chiropractor, but she has seen-- over the last six years, she has seen the weight gain since the first accident. She seen the 30-pound weight gain in like two months from the accident. But, I mean, I've always had really good blood pressure. My sugar levels are great. My cholesterol levels are low. So, for me, I guess, internally, I'm healthy other than being a big person.*

Excerpt - Document: 146 Respect Interview- cleaned.docx, Position: 14545-17957

*Can you think of any policies in a medical institution which show a lack of respect?*

*S2 19:16 This may sound like a [inaudible] on my part, but nowadays, if you ask somebody if they're male or female, that seems to get everybody in an uproar now because they don't identify with either, or their birth certificate says one, but they identify as the other, and then that causes problems. But I don't--*

*S1 19:40 So what about that makes you feel like it's a lack of respect, if you don’t mind expanding on that?*

*S2 19:47 For me, I don't care. Just whatever your birth certificate says, that's what you are. But for some that they're born male, and they feel that they're female, they view that as, "Oh, you called me by the wrong gender. That's not what I am. How dare you?" And then they get all uppity and pissy about it. It's like, "That's what's on your birth certificate. That's what's on your driver's license. If you haven't changed your driver's license over, that's nobody's fault but your own for not fixing it or changing it." But your birth certificate determines whether you're male or female when you're born, so it's one or the other. So there is no other gender because you're male or female, period. So, for me, I could see how the people in that community would be offended, but they get offended over everything else too. So everything's offensive nowadays to most people.*

*S1 20:41 Okay. Well, any other policies that you can think of in a medical institution that show a lack of respect?*

*S2 20:50 Well, I do think it's funny how-- I don't think it's offensive, but I just think it's funny how on the race thing when you're filling out paperwork, it's got all the different ones except it goes to white. It doesn't say Caucasian. It says white. So okay, like Island Pacificer, Hispanic, Latin, African American, all the different ones, but then it says white. So I think that's kind of comical. I could see that offending somebody too. But policy-wise--*

*S1 21:30 Sorry. I want to go back to that a little bit. What about that do you think you or other people could find offensive about it just to--?*

*S2 21:40 So we're in the state now where everything has to be politically correct. You can't say black. You have to say African American. You can't say Mexican. You have to say Hispanic. There's certain words you can't use. But when it comes to white people, then you can say white. White's okay, but you can't say black. You actually have to pronounce African American. Well, if they're not American, it can't be African American, so that doesn't work. But for white, they're slowly switching over to Caucasian because that's offending white people, which it doesn't offend me. I'm like, "Whatever. My skin is white. I'm white." But for my son, who is half Native American and half white, that's weird. White isn't a color when everything else is the definition, but white is white. But a few people get uppity about that. Like I said, I don't. I don't really care what color you are. I don't care about any of that, so.*

*S1 22:49 Okay. Thank you for sharing that. Any other examples that you can think of or things that you can imagine that would be offensive-- sorry, offensive. I just got that word stuck in my head. That would show a lack of respect in medical settings for you?*

*S2 23:10 No, I cannot. That would be it. Just the little things that people get upset about because of verbiage.*

Excerpt - Document: 148 Respect Interview - SP English Translation-cleaned.docx, Position: 3352-4543

*have you ever felt that the people in the medical care environment haven’t respected you due to who you are? For example, because of your race, your ethnicity, your language, things like that. You’ve never felt like you’ve had--?*

*S2 06:34 Well, yes, on one occasion, because I don’t speak perfect English.*

*S1 06:40 Could you tell me a little more about that experience, or--?*

*S2 06:47 When I was sent to a research study and the person who assisted me spoke English, and I answered her as best I could, that’s when she could tell that I didn’t speak English perfectly, and then afterwards she didn’t even face towards me to ask me things. She just packed up her things, in this rude way.*

*S1 07:11 That’s a very interesting example because sometimes one hears examples like what you said, when one can’t speak Spanish or English, and sometimes people get frustrated and don’t feel respected. Now, could you imagine something that, apart from that, would make you feel that someone doesn’t respect you? Apart from the language or communication problems?*

*S2 07:44 No, just that situation, the one I told you about, when she started to carry on with things as though she were upset, this person.*

Excerpt - Document: 148 Respect Interview - SP English Translation-cleaned.docx, Position: 4553-4924

*And now, I wonder if you can tell me about the policies that a medical institution has. For example, maybe you can think of some rules and a clinic might have, where you’d say, “That doesn’t demonstrate respect to me.”*

*S2 08:18 No. The rules that I’ve seen so far, I think, demonstrate respect to any person, independently of their race, according to the rules they have.*

Excerpt - Document: 133 Respect Interview-cleaned.docx, Position: 5747-6789

*Have you ever felt a lack of respect in a medical setting based on who you are or how you look or what you believe in?*

*S2 07:45 That’s a hard question to answer. Again, not so much a lack of respect but more ignorance a little bit just because of culture that they don’t fully understand. I don’t think I feel that they are disrespectful in that sense, but I do feel like they don’t truly understand culturally my background. For instance, with the study, why I try to do it with the CHARM Study, do the genetic test, I don’t know very much of my background because where I was born and where my family is from, they don’t have a good history of problems. And it’s pretty much a third world county. So coming from that, and then coming to modern medicine, I don’t have a lot of those answers. So not having some of those things, they’re not understanding the culture of it and why we don’t have that kind of record keeping. It does feel a little bit of a disrespect or ignorance, more like. But yeah, that’s really the only thing on that part*

Excerpt - Document: 133 Respect Interview-cleaned.docx, Position: 6986-7713

*But can you imagine anything that might make you feel a lack of respect truly or maybe something that you can imagine happening to your friends or family?*

*S2 09:38 Yeah. Insurance, I think, plays a big part. I know a lot of my family members, the older generation, who have come to the United States, a lot of them are on Medicaid. They have barely grade-school education and barely write their names, let alone speak the language. And when you go in to see a provider and them not giving you the time or the full treatment, would be totally disrespectful, I would think. And not treating them fairly or not treating them like they would another person who spoke the language, I think would be a total disrespect in healthcare.*

Excerpt - Document: 133 Respect Interview-cleaned.docx, Position: 8323-8860

*So can you think of any policies, maybe, that a medical institution might have that would show a lack of respect?*

*S2 11:51 Yeah. If they capped certain number of patients being seen with that specific type of insurance, I think that would be really disrespectful. I know state and federal insurances don’t-- or Medicare and Medicaid don’t take quite as much as, say, [inaudible] insurance by putting a top on that so that they can restrict the access, I think would be really disrespectful to them as humans, to deal with medical care.*

Excerpt - Document: 157 Respect Interview-cleaned.docx, Position: 3531-4703

*And so I know you mentioned some negative experiences you've had in the past, but have you ever felt a lack of respect in the medical setting based on who you are or how you look or what you believe?*

*S2 08:39 Sometimes I wonder if that will happen because, sometimes, I don't really dress up for my doctors' appointments. I may kind of look like a bum. I kind of rush. But I think, overall, I've been treated okay. I don't know. In the past, I've had some back problems, and I did have to go to the ER, and I don't think they believed me that it hurt as bad as it did because I had to lay there for a while before they would come see me. And then when they gave me a shot, it was like it wasn't enough. So I think with the opioids, it is hard whenever you really are in pain because they don't want to give you anything. So I think that's a delicate balance because you know you don't have any opioid addiction issues, and it's like that's why pain care is there, but I guess at the same time, the doctors have-- they don't want to enable people or whatever. I don't know. They just don't want to give any drugs to anybody, so it's like an issue when you really have pain.*

Excerpt - Document: 157 Respect Interview-cleaned.docx, Position: 4718-5771

*And so thinking a little bit about the policies again, in your opinion, do you think there are any policies that show a lack of respect?*

*S2 10:13 I think it just comes down to pain management, probably, yeah, related to that. It's probably hard for them too. But I was in a car wreck last year, and I couldn't move for two months. I literally couldn't move, but they didn't give me any pain medication. So I don't know. To me, that's kind of a problem with healthcare right now. And then also, I would say, too, maybe the lack of natural remedies. Of course, they always want to prescribe you something that costs a lot, but sometimes there are natural remedies. I don't know. One time I went to the urgent care because my shoulder was hurting really bad, and she was like, "Oh, well, you just need to do this exercise," and that was kind of nice to be able to have a solution that wasn't a pill. Yeah.*

*S1 11:17 So maybe providing you with some alternatives to the normal, like you mentioned, pills and those types of medication.*

*S2 11:28 Yeah, yeah.*

Excerpt - Document: 168 Respect Interview - SP English translation - cleaned.docx, Position: 2990-3503

*Okay, so how about this, could you imagine something that would make you feel disrespected?*

*S2 05:21 Imagine it? Well, once, regarding the administrative part, a person did treat me a little badly, but that was regarding the administrative part, in matters of, how do you say that? As if it were because of racism, like the matter of citizenship, that does make me a little bit, like--*

*S1 05:49 No, yes, sure, sure. So, you said that, this happened to you once?*

*S2 05:56 Just once, yes, an administrative issue.*

Excerpt - Document: 168 Respect Interview - SP English translation - cleaned.docx, Position: 4555-5316

*Sure, okay. Now, I want to ask you about the policies that a medical facility has. What types of policies would show disrespect?*

*S2 07:44 Once in a while, those that I mentioned, such as the immigration situation, that once in a while, once in a while, finding any obstacle, looking for any obstacle to deny the service. By not denying the services in question, of the hospital, because the hospital already has what is [inaudible] (the cover), to deny the administrative issue, like I’m telling you, denying, in terms of discounts, credit, yes, they find any obstacle.*

*S1 08:15 Okay.*

*S2 08:16 And, once in a while, also in the hospital, in that, yes, sometimes they take too long to check [crosstalk] that service, but the rest, it’s all really good.*

Excerpt - Document: 168 Respect Interview - SP English translation - cleaned.docx, Position: 15471-15879

*And now, again, could you tell me a little bit more about why you answered how you answered? You gave a four this time for trust, regarding medical attention.*

*S2 22:59 As I said, yes, once with an administrative issue, yes, like they treated me a bit badly. So, yes [inaudible](and, so well) , it was that, like that creates a little distrust regarding the [inaudible](question asked), but it was only that.*

Excerpt - Document: 143 Respect Interview-cleaned.docx, Position: 4230-5753

*That 45-minute wait that I talked about earlier, it wasn’t, I guess, so much the staff doing something as not doing something. Because during that time, there was another patient who decided she wanted to talk to me, and she was just saying all sorts of racist and sexist and just all sorts of things that I didn’t want to talk about, trying to talk about politics and all sorts of things when we’re waiting in the doctor’s office. And you could see that the other patients were uncomfortable including myself, and none of the staff came out and asked her to stop.*

*S1 [04:14] Okay. So inaction on the part of the staff and maybe not maintaining kind of a-- oh, what’s the word I’m-- well, not maintaining a respectful atmosphere for everyone.*

*S2 [04:29] Right. A trans woman came in, and she was talking so loudly about that he/she over there and like, “Urgh.” Once that person sat down, I went and moved over there. But it [inaudible].*

*[momentary loss of connection]*

*S1 [04:57] So you had mentioned that there was this other patient that was not being very respectful of the other people that were coming into the clinic.*

*S2 [05:06] Oh, yeah. Every person that came in, she had something negative to say about like, “Well, maybe you just--” and she kept complaining that she thought that the staff were moving her appointment farther back and back. And I wouldn’t be surprised if they had because of how rude she was [laughter], but they also didn’t do anything to stop her or help anyone else feel comfortable.*

Excerpt - Document: 143 Respect Interview-cleaned.docx, Position: 7827-8404

*But can you think of any other policies that a medical institution could have in place that might show a lack of respect?*

*S2 [08:49] Policies that show a lack of respect? Not really. I mean, I imagine there are some out there that I don’t know about because I’m just not that other person. And I don’t know what it’s like for trans people in the medical industry, but I didn’t get the impression that the one woman I talked to after I moved seats, it didn’t sound like she had had a great experience. But she was excited about new policy changes and that it might get better.*

Excerpt - Document: 106 Respect Interview-cleaned.docx, Position: 13055-14024

*Is there any other ways that you think might be?*

*S2 22:09 If it's looking at medical records?*

*S1 22:12 Yes.*

*S2 22:12 Like you're asking specifically [inaudible]. Yeah, I guess to-- I feel like it's so important to also have a team-- when they're talking about people to not be judging people [laughter]. Because I used to [share?] an office space with these doctor's and I was just shocked by how much they would just talk negatively about the patients while they were reviewing stuff. And I was just like, "Oh, my God, why can't people just be more neutral and not impose their judgments these people who are people?" But when you're just reading through stuff you're like-- so, yeah, I think just having a team that's not making fun of people's medical records. I know it's something you can't control for necessarily, but just being really professional about it would be a plus. I know the patient never hears it, but [laughter] there's other people that hear it.*

Excerpt - Document: 131 Respect Interview-cleaned.docx, Position: 3072-5095

*So for this next question, I’d like to kind of flip the previous question on its head and ask you to give me some examples of the sorts of things that might make you feel a lack of respect in medical care.*

*S2 07:16 Okay.*

*S1 07:17 So I imagine that a lot of it could be just kind of the reverse of what we had talked about before with communication, and confidentiality, and privacy. But if there’s anything else that stands out to you or maybe any experiences that you’ve had in the past--*

*S2 07:36 Sure. Well, yeah, like the thing I mentioned before, where random self issues come up and the doctors blames weight management for the problem, rather than listening to the problem. I would say that that is something that not only has applied to me in the past, but people I know, where they don’t even want to go to the doctor. Because they don’t want to get lectured about their weight, or their diabetes, or whatever disability they have that they feel like the doctor blames every health problem on the one thing rather than looking at the person as a whole. But not everything is all about their diagnoses. And yeah, just the opposites of what we talked about a moment ago, like just feeling lot listened to, or dismissed, or lectured, rather than sharing information and feeling like you’re supported.*

*S1 08:45 Okay.*

*S2 08:49 And I’m also a person who likes to ask a lot of questions. So I appreciate it when a doctor is honest with me, like, “Well, we don’t know why it’s this way. But what we’re going to do to try to find out is this, this, and this.” Or explaining what the diagnosis means, rather than just saying, “Oh, it’s this thing. Here’s your prescription. Call me if it doesn’t get better.”*

*S1 09:13 Okay. And so I think those are interesting. Because there are also aspects of that communication element that we talked about with honesty being kind of an open line of communication. Again, you just mentioned being given information instead of handed a prescription and sent out the door.*

*S2 09:44 Right.*

Excerpt - Document: 117 Respect Interview-cleaned.docx, Position: 2669-2830

*Have you ever felt a lack of respect in the medical setting based on who you are, what you look like, or what you believe or anything like that?*

*S2 07:13 No, no.*

Excerpt - Document: 145 Respect Interview-cleaned.docx, Position: 3169-3859

*And have you ever felt a lack of respect in the medical setting based on who you are or how you look or what you believe in?*

*S2 06:59 I think one time I saw an OB/GYN, and she asked me if I was planning on having kids. It was one of those kind of conversations. In terms of birth control. And I told her no. And her automatic response was why not? She was like, "I keep hearing this more and more these days." And it sounded more judgmental than like a curiosity of, "Well, why do you think that is?" It was just like, "Why are millennials not wanting children anymore?" Or something like that.*

*S1 07:31 Okay. So sort of a, as you said, judgmental response from a specialist.*

*S2 07:38 Yeah.*

**Logistical communication**: Communication and follow-up between provider/staff and patient, including timeliness of communication; does not include quality of conversations

Excerpt - Document: 127 Respect Interview - SP English translation - cleaned.docx, Position: 706-954

*could you give us some examples of the things that make you feel respected when you receive medical attention?*

*S2 00:16 Yes, well, since I speak very little English, I like to be-- they find me a translator and everything; that makes me feel good.*

Excerpt - Document: 127 Respect Interview - SP English translation - cleaned.docx, Position: 3161-3962

*So since you don’t have examples, what about if you could imagine for yourself something that would make you feel disrespected or that your friends or family members have been disrespected? If you can, think of examples. Or something that just wouldn’t make you feel respected.*

*S2 04:10 Yes. One example is that my [family member] had this pain and he went to the clinic and they told him that they were going to run some tests on him and, well, they never called him to-- they were going to call him to make an appointment in another clinic and they never called to schedule it. He called them and they told him to wait because they had said they would call him, but they never did. Those are things that don’t--*

*S1 04:32 Yes. Right, that --*

*S2 04:34 --that wouldn’t be good. But that’s not the case.*

Excerpt - Document: 118 Respect Interview-cleaned.docx, Position: 387-936

*So could you give me some examples of the sorts of things that make you feel respected in your medical care?*

*S2 02:40 I think number one, being the provider being on time or at least communicating if there's a delay with the appointment. I think that that's huge. I think that providers taking the time to acknowledge that-- especially, if you've been through something kind of scary or traumatic, taking the time to acknowledge that and answer your questions maybe more in-depth then what you might even be aware that you have deeper questions on.*

Excerpt - Document: 118 Respect Interview-cleaned.docx, Position: 964-1425

*So just thinking a little bit more broadly, about the larger medical staff, say the nurses or even some of the reception staff, can you think of anything that they can do that would show respect?*

*S2 03:52 I mean, other than just communication. A smile is always appreciated. I mean, really with the nursing staff, I mean, a pleasant attitude, a smile I think go a long ways. Yeah, other than that I can't really think of anything that pops into my head that...*

Excerpt - Document: 118 Respect Interview-cleaned.docx, Position: 1508-2077

*can you think of any policies that the medical institution could have in place that would show respect?*

*S2 04:54 Any policies.*

*[silence]*

*S2 05:13 As far as the institution goes, I think just having plans in place for if a provider is late or needs to cancel. Just making sure that there's a good communication, checking in as well, I think, too to make sure-- surveys to see possibly how your appointment went if there was any concerns that you felt that should have been addressed that maybe wasn't. I think that's kind of what I'm thinking at least, at this point.*

Excerpt - Document: 172 Respect Interview - SP English translation - cleaned.docx, Position: 1192-1374

*Could you give me some examples of things that make you feel respected in your medical care?*

*S2 02:54 One’s privacy and the communication they keep up with you. The trust one feels.*

Excerpt - Document: 136 Respect Interview-cleaned.docx, Position: 3226-3632

*Could you now give me some examples of the sorts of things that would make you feel maybe a lack of respect in your medical care?*

*S2 07:54 Yeah. Maybe getting my information wrong, like my name or my chart. Not getting my questions answered. Kind of getting brushed off. Wow, there's so many examples. Having an even longer wait time [laughter]. Or having rude doctors or nurses. Is that enough [laughter]?*

Excerpt - Document: 136 Respect Interview-cleaned.docx, Position: 3913-4428

*So you talked a little bit about getting information wrong, or not feeling listened to or being brushed off. So with that, are there any other examples that make you feel disrespected maybe? Or you can maybe dive into a little bit more about why those examples make you feel disrespected.*

*S2 00:52 Yeah, I can give further explanation on what I've already said. Getting my information mixed up, I mean that would be a really big issue. I mean if they didn't have the correct information how can they help me, right?*

Excerpt - Document: 123 Respect Interview-cleaned.docx, Position: 9072-9894

*I’m going to ask the same question as before, which is are there any policies that you could think of that might show a lack of respect from an institutional level?*

*S2 12:17 No. Well, I don’t know if they have the policy but I-- first of all, [healthcare institution] is not necessarily a teaching hospital. But I understand they provide the interns their practicums from a university. So I honor and respect that but I think that at a point, you should probably ask and I don’t know if they have policies about it. So maybe they should implement those where they ask the patients privately without the student being present so we’re not put in a precarious spot. And then, if there’s any hesitancy, you can explain that or you can just accept no and move on. I’m sure there’s plenty of patients that would gladly consent.*

Excerpt - Document: 142 Respect Interview- cleaned.docx, Position: 3098-4092

*People have answered that question differently. Some people bring up examples like reminders or policies that their hospital or clinic might have in place to remind them to come in or things like that. Privacy is a big one. So people answer differently. If you don't have anything to answer, no problem. We can move on.*

*S2 07:00 Okay. Yeah, I'm trying to think. So yes, I definitely-- I do appreciate because it's like we are so caught in our busy day-to-day lives, that it's nice to have a reminder of any follow-ups that may be needed. For example, when I went to my annual exam a few months ago and that was my first time meeting with [inaudible] physicians besides the doctor I'm with, I had questions regarding some cancer history within my family. So the nurse practitioner [inaudible] had provided some resources. And I appreciated that, knowing that I could rely on some medical professionals to provide information in regards to what my results were and how I can get more information.*

Excerpt - Document: 151 Respect Interview - SP English translation - cleaned.docx, Position: 2178-2926

*So, how about the clinics where you go, are there certain rules or policies that a medical institution has--? As I said, are there rules that the clinic has, that you think show respect?*

*S2 04:47 How? I don’t understand.*

*S1 04:59 Sure. For example, in some clinics when they call out your name to ask questions, they have you stand, to be next to the receptionist, so that other people can’t hear your medical problems. Do they, sometimes, do that at the clinics?*

*S2 05:22 No, the people that I’ve gone there with, there haven’t been occasions (unable to make out inaudible) [inaudible] confidential. I think it’s normal that I have to be with the indicated person, not for privacy, to stand up and go, with respect towards the person at the front.*

Excerpt - Document: 151 Respect Interview - SP English translation - cleaned.docx, Position: 4105-4729

*That’s a good point that sometimes they aren’t showing you all the respect, because sometimes you don’t notice it. But you say that you haven’t noticed it, and you haven’t felt a lack of respect.*

*S2 07:47 No, no sir, they have taken great care of me. The whole process has been in my language. There’s been no obstacle for me to communicate with them or they with me, because they have the possibilities to have the interpreter. They speak in Spanish or immediately ask me, “Do you want English? Do you want Spanish? Do you want this to come out?” They are always with the patient, who is satisfied with the given treatment.*

Excerpt - Document: 129 Respect Interview-cleaned.docx, Position: 2302-3652

*So with this one, can you give me some examples of the sorts of things that might make you feel a lack of respect with your medical care?*

*S2 05:36 Well, I do feel and maybe I’m-- I mean, it’s just the way I feel. And I’m sorry to just kind of bring back again my pregnancy and stuff but that’s been my biggest experience with the hospital and medical care in the state. So I would have to say that I kind of felt a little pressure to have an induced delivery which I was not really-- which I did not want. So I did feel like they were kind of pressuring me too much and at the moment, I was-- well, of course, it was the first time for me having a baby so I was not really thinking and I just felt I got too much pressure into telling me, “You have to do this and you cannot leave,” and that kind of thing. So that was the only thing that I was not very happy with anybody. I just wanted like a little bit of time and maybe like some more time to talk to doctors and that they will kind of sit and tell me more like consequences and all those things.*

*S1 06:59 Okay. So feeling a little bit of pressure and not having the opportunity to kind of start that line of dialogue or the communication that we spoke about earlier.*

*S2 07:10 Yes. I mean, before that I had like-- I don’t really have any complaints but just like for my delivery, I do have some.*

Excerpt - Document: 129 Respect Interview-cleaned.docx, Position: 17985-18724

*All righty. And so how does your trust in research compared to your trust in medical care?*

*S2 28:37 Good question. Let me think about it.*

*S1 28:42 Take your time.*

*S2 28:47 Well, I don’t know if it’s-- I don’t know like research for some reason made it for me very easy to trust that this was going to be well legit and I was going to get the information and they were very, very clear with emails or phone calls of the next step or what it was going to happen which was really good. I would say that sometimes it might be harder to get a hold of my medical team. I guess like my doctor and staff, I find it a little more difficult. Also, I always feel very comfortable when I see my doctor. I don’t know if that might answer your question.*

Excerpt - Document: 126 Respect Interview-cleaned.docx, Position: 4289-5162

*And is there any specific examples when it comes to maybe any of your interactions that you’ve had with providers or, again, the nursing staff, reception staff that come to mind for you when it comes to a lack of respect?*

*S2 09:11 I can’t think of any specific interactions, but I guess I feel like-- I mean, I know whenever I call to make an appointment, it’s always very formulaic. They always ask for my medical record number and all these numbers that I have to give before I get to actual interaction with anyone. That’s something that comes to mind. Yeah.*

*S1 09:44 And is that usually through some sort of automated system, or is it just the person on the other end of the phone just asking for numbers?*

*S2 09:50 No. It’s the person on the other end of the line. Yeah. Yeah. Sometimes it’s automated, but, yeah, I’m thinking of more like when I’m talking to a person.*

Excerpt - Document: 158 Respect Interview - SP English translation - cleaned.docx, Position: 1783-2124

*As an example of how they treat me or what it’s like? When you make an appointment, I make an appointment, they make it quickly or as quickly as possible. That’s something that I love, that when I call the clinic, if they don't have it for that same day, they give it to me for another day or so, as quickly as they can, they make it for me.*

Excerpt - Document: 116 Respect Interview-cleaned.docx, Position: 433-990

*So could you give me some examples of the sorts of things that make you feel respected in your medical care?*

*S2 02:58 I think, so far - knock on wood - I haven’t had to get much medical care lately. But when I have, I like-- I’m trying to think here. I guess the big things for me would be consideration of privacy and their timeliness on how they get back to me about results on stuff if I have any tests done or whatnot. And just giving me as much information as possible about any questions I may ask about medical issues that I have, that sort of thing.*

Excerpt - Document: 113 Respect Interview_cleaned.docx, Position: 3167-3783

*So to kind of flip that first question, could you give me some examples of the sorts of things that might make you feel a lack of respect from your medical care? I know you mentioned a couple when we talked about the staff. But if there's anything else that comes to mind?*

*S2 06:30 I know this is a problem but if you get a-- when you're trying to get a hold of the medical staff for either a question or a concern and I understand that they're not available. But when they promise they call back and the call-back doesn't happen till four or five days later. I feel that's a lack of respect for the patient's care.*

Excerpt - Document: 146 Respect Interview- cleaned.docx, Position: 5111-6698

*Can you think of any policies that a medical institution has in place that makes you feel like they're respectful? What kind of policies show respect?*

*S2 08:41 I mean, I guess it shows respect with a policy that drives me nuts. Every time I go in for an appointment, whether it's my annual whatever or if I've seen three different doctors that day at the same clinic, every single one asks, "What's your last name? What's your date of birth? Why are you here?" blah, blah, blah. I guess they have to ask that, but when I'm in the same office for three hours seeing three different doctors, it drives me up the wall. I mean, it's great practice because you're verifying who you're talking to but literally have not left the room at all, and you send three different people in back to back. Yeah. I mean, it's good practice, but it's annoying as heck. I'll tell you that. It drives me up the wall. Like, "I know she handed you over my file immediately," but two seconds in between when the doctor left and the new one came in. I'm like, "Hmm, but you got to ask and verify everything again?" But I get it. You have to do it just in case. It takes that one person to mess things up and then lawsuit central. I get it, but it's definitely a bit annoying, especially when you have to go in every six months and see the same doctor, and then they ask, "Why are you here?" "Really? Look at my notes." They always ask you, "Oh, well, why do you need to be seen?" I tell them why. "Why are you asking why do I need to be seen?" But I get it. They have to. They're consistent. I'll give them that.*

Excerpt - Document: 133 Respect Interview-cleaned.docx, Position: 2768-3385

*could you give me some examples of the sorts of things that make you feel respected in medical care?*

*S2 03:11 Yeah. So, the doctors and the medical staff being on time, getting prompt visits definitely makes me feel respected. Just getting the information timely, if I had test done I do want some information quickly so that I can see what my results are. That’s mainly what I feel would-- yeah, as far as doctors go, I don’t see very many doctors. So I would say I’m a pretty and generally healthy person. So there’s not many occurrences where I have gone in and did not feel respected on my visit with a provider.*

Excerpt - Document: 133 Respect Interview-cleaned.docx, Position: 3503-3951

*And so could you maybe go into those a little bit more and tell me what it is about that exactly that makes you feel respected?*

*S2 04:28 Yeah. It makes me feel like my time is valued as well. And I know in doctors you have the higher [inaudible] and whatnot, and their time is valuable as well. But just going that extra mile and staying on time or giving me that prompt information, makes me feel like my time or my health issues are top priority.*

Excerpt - Document: 143 Respect Interview-cleaned.docx, Position: 3522-4187

*But could you give me some examples of the sorts of things that might make you feel a lack of respect in medical care?*

*S2 [02:33] Like I said previously, when there’s a delay and there’s no communication about the delay, that’s frustrating because I know that they’re time is important, but mine might be, too. And let’s see. [inaudible]. I guess if they just come in, they call your name in the waiting room and then they just bring you to the doctor’s office and there’s no communication at all between that, it feels like they don’t want to be there. And I mean, I know it’s their job and they might not want to, but that makes me feel a little more stressed.*

Excerpt - Document: 106 Respect Interview-cleaned.docx, Position: 1624-3117

*are there any maybe specific actions that a medical provider or the nurses or doctors kind of performed, for lack of a better word, that really exemplified respect?*

*S2 04:46 Yeah. For one knowing my case. It was really hard for me when I came in and someone was scribbling on a piece of scrap paper [laughter] and didn't seem to know what was really going on with me. And I'm like, "Oh, my God." It made this appointment-- you should have read up a little bit before you came into the room. So that kind of stuff would be frustrating. And then in the healthcare system I was in, they kind of just scheduled you with whoever was available that day, so I felt like that made it hard too. I didn't have a consistent person that I saw other than the doctor who did my ultrasounds. But he deliberately made the choice to be like, "I'm going to do her ultrasounds. I don't want anyone else doing them." I didn't know at the time that you can do things like that. I just thought you had to go with whoever they gave you. So it was really nice having a consistent person who saw you. And then if someone didn't know you, that they followed up and-- follow-up was huge too. Feeling like, "Okay, this person is going to call me about the results," or they want me to come back in two months, or whatever, to make sure I'm not having these symptoms again. It was more like I had to make that call, so that was just difficult for me to navigate and feel that people really cared about what was happening.*

Excerpt - Document: 131 Respect Interview-cleaned.docx, Position: 1474-2136

*can you think about how the other individuals that you might interact with, say, the nurses or the reception staff, can you think of any ways that they might show respect?*

*S2 04:25 Well, I think good customer service skills, obviously, which I know is really hard after long days and things. But [laughter] good customer service is one. Again, communicating well, keeping me up-to-date if there’s a long wait for whatever reason, just letting me know, not just leaving me sitting in the waiting room forever [laughter]. Maybe just, I guess, feeling acknowledged, listened to, and that I’m not just another number in the line of the list of people to get through.*

Excerpt - Document: 131 Respect Interview-cleaned.docx, Position: 2163-3044

*And to kind of think about this in an even broader sense, if you think about the medication institution itself, whether that’s your healthcare system or this clinic, can you think of any policies that these institutions might have that can show respect?*

*S2 05:48 Well, I don’t know of any policies, specifically, if that’s what you’re asking. I’m not sure if I understand the question entirely.*

*S1 06:03 Or is there any that you could-- if there’s anything that you can think of that might be a good policy for maybe some clinics and healthcare systems to implement.*

*S2 06:15 Gotcha.*

*S1 06:16 Yeah.*

*S2 06:17 Yeah. Let’s see. Well, I think good policies would be returning phone calls in a timely fashion during business hours, making sure that confidentiality is a high priority. In general, having good training and support for their team so that everybody is happy in their jobs.*

Excerpt - Document: 145 Respect Interview-cleaned.docx, Position: 2110-3095

*So now, I want to kind of flip all those questions on their head and ask you if you could give me some examples of sorts of things that might make you feel a lack of respect from your medical care?*

*S2 05:15 Not following up in a timely manner if you're waiting for any type of results. I don't know if it's disrespectful, but I think it goes back to what I was saying before. Just making sure that someone gives you time and then again, that feeling where actually makes me feel like my issue is not necessarily important. And not having a professional demeanor. I mean, I know we all have bad days, but sometimes people get snappy and healthcare is a stressful job. Let's be honest. So I get it to some degree but not taking that out on your patients.*

*S1 06:09 Okay.*

*S2 06:19 Let's see. And I think if you don't get a full picture of what medical costs are going to be. So I guess not being thorough and transparent and sort of fees-- or again what's happening and why it's happening.*

**Neutrality**: Encouraging decisional autonomy, i.e., allowing and supporting patient to make own decisions

Excerpt - Document: 107 Respect Interview-cleaned.docx, Position: 538-2060

*Yeah. So, for example, some people think about the way that medical staff interacts with them. Other people talk about maybe the sorts of policies that a medical institution has. Do any of those-- can you think of any examples of things that would make you feel respected from either of those perspectives?*

*[silence]*

*S1 03:36 Another way to maybe think about is if there is-- if you can think of a specific example when you felt respect from your medical care.*

*S2 03:45 Okay.*

*[silence]*

*S2 04:11 When I've made a choice with my care and my doctor understands it, I guess.*

*S1 04:29 So having a choice about the sort of-- do you mean about what doctor you see or do you mean a choice about whether you do something that they recommend or not?*

*S2 04:44 Yeah, that one.*

*S1 04:45 The choice about what you do with your medical care?*

*S2 04:50 Yeah.*

*S1 04:50 Can you think of any examples of when you've been able to do that? Or maybe if you have a doctor who's really good at helping you or allowing you to have that choice?*

*[silence]*

*S2 05:35 Well, [there's one that?] I think-- I may have chosen not to continue taking a certain-- I don't remember what medication it was, but it was not helpful, so I decided that it wasn't working and told my doctor about that.*

*S1 06:03 And how did your doctor react when you said that it wasn't working?*

*[silence]*

*S2 06:43 Just said that we can stop it.*

*S1 06:45 So it sounds like your doctor actually listened to you and let you make that decision for yourself.*

*S2 06:55 Yes.*

Excerpt - Document: 122 Respect Interview-cleaned.docx, Position: 412-1009

*So could you give me some examples of sorts of things that make you feel respected in your medical care?*

*S2 03:11 I guess a doctor not feeling like they’re rushing me to get through to get to the next patient. I like when they listen and kind of include me in I guess the decisions that are being made. I’m not just telling that you need to do this and this and this when they-- like one of the things that my doctor says, “Well, you know, how about if we try this? What do you think?” I guess having the patient participate in the care as well and not the doctors who’re making all the decisions.*

Excerpt - Document: 122 Respect Interview-cleaned.docx, Position: 9328-10832

*So sometimes people say that their experiences in the medical setting can affect whether they want to join research studies. Was that at all same for you?*

*S2 11:29 Not, not really. But I can say I guess in some ways I can relate for some people which feel more comfortable, the way that the information came to me via computer that, “Hey, we’re doing this study. Are you interested in joining? Then, maybe I guess being in the clinic and having somebody approach somebody and feeling on the spot. Everybody’s so individual but you know a lot of people are a little bit more nervous and uptight when they go to the doctor anyway. And so I guess sometimes just having that information to sit and read over it and discuss, I guess could be not in-- I'm not getting the word that I’m looking for. You don’t feel quite as pressured.*

*S1 12:23 Okay. So with that in person interaction especially when you’re kind of at the doctor for a specific purpose-- sorry, go ahead.*

*S2 12:32 Yeah. Right. Yeah. You would feel more pressure in person than being able to say, “Hey, we’re doing this study. Here’s the information, read over it and if you’re interested, contact us” or whatever that kind of a thing. Because I think sometimes some people-- no. I wouldn’t personally but I know how other people are and obviously being in healthcare myself, I see a lot of phobia in patients and so I think, yeah, sometimes people make decisions quickly or feel pressured and that really isn’t what they want or whatever, so.*

Excerpt - Document: 123 Respect Interview-cleaned.docx, Position: 4119-8710

*you sort of alluded to this but can you give some examples of times when you felt a lack of respect in medical care?*

*S2 06:52 Yeah. So on my journey with stomach stuff, when I was going in-- I mean, I was throwing up in my sleep and so I went into the examination room. So we don’t know if they’re primary care and the woman I saw, I said, “I’m throwing up almost twice a week in sleeping mode.” And she’s like, “Well, you only have two episodes. That’s good.” I’m like, “I don’t think that’s true.” You know, I get it. I’m a [occupation], I’m not a doctor but that doesn’t make sense because you can die when you’re hearing that phrase, that’s disrespect. And so she was just like, “No. Two episodes is good.” And I asked to talk to a specialist and she said I didn’t need to see a specialist. So she really didn’t validate my feelings nor did she wish my medical desire which I felt was ridiculous. And then, I went back. A week later, once again I was throwing up in my sleep. I went in and saw-- I don’t know if he was a nurse practitioner or something, and he was just like, “What? Some people are thin donuts and some people are fat donuts.” And he actually compared me to like a glazed, sprinkled Mustang donut and was saying that my digestive system is like the center of that donut and if I were just as more compact donut that was more fit, my food would just go through me. But since I was more spread out like that fat sprinkled donut, then I was going to continue to have problems, and I was just more shattered. Then, I said, “You know, as I’ve gone through this journey and I extraordinarily lost a bunch of weight and I’m still working out.” And then I got shin splint. I tore my rotator cuff. I’ve had a series of cortisone shots and all of these things, and I’m kind of afraid to jump back. And your family is like, “Well, a cortisone shot is better than not being fat.” And I think it could probably be two ways.*

*S2 08:50 So again, he was just so quick to jump on the weight bandwagon. He wasn’t listening to my symptomology at all, and he was just like, “Well, you know.” And then, this was like minimize shame and it seemed ridiculous to me also like is that really the best solution our medical providers can give us. It’s like, “Well, you’re obviously in some pain. Just go get another cortisone shot” which is weird because there’s research about cortisone shots that you’re only supposed to have so many and he was just dismissive of it. But then I had a really bad example where I had had a bad pedicure but not a chronic issue. But I had a bad pedicure and I had gotten an ingrown toenail. So I went in and I was telling the provider. They’re like, “Oh, just soak it.” So I did and it didn’t resolve. And so it ended up being where I had to have it removed. And I don’t what he was like a physician’s assistant or something. He had a student come in and said, “Do you mind the assistance?” I’m like, “You know, I really kind of prefer, no offense to you.” Whilst she’s trying to learn and now what has happened is-- so I felt pressured. He said, “She’s a very nice old student.” So I’m like, “Okay. She can assist.” They didn’t know my foot. My foot ended up being-- I don’t know. Well, at least 12 shots in my toe, so the toe ended up being bruised for a week. They removed half of my toenail. And because students used like a little bottlebrush with the root. They killed the toenail root. My toenail now can only grow about a quarter inch out and then it dies because they’ve permanently damaged my root. So I don’t know. Well, it wasn’t respectful. I didn’t want the student. They probably shouldn’t ask if a student is right there because it puts the patient in a precarious spot. When I said no, he didn’t accept no, “Oh, common. She’s just trying to learn; I’ll be here.” And then, she failed with that and now I have a permanent like-- does it kill me? No.*

*S2 10:52 But I’ve had to have that toenail removed a couple of times since then. I never had ingrown toenails before. It wasn’t like I had a chronic problem where I needed that level of treatment. And now, it literally dies off and it turns like almost a bluish color. It’s unsightly that doesn’t kill me but had he just listened to me in the first place perhaps I wouldn’t have this and I actually asked to see a specialist for it. And my providers said I didn’t need to.*

*S1 11:25 Yikes. Well, I’m sorry to hear you have so many examples of-- that sounds rough so I’m sorry you had to go through that.*

*S2 11:35 Like if I’m old, over 45 years, this wouldn’t have been bad, but I’m [in my early 30s].*

Excerpt - Document: 115 Respect Interview-cleaned.docx, Position: 430-824

*So could you give me some examples of the sorts of things that make you feel respected in your medical care?*

*S2 03:20 I’m going to look to my last appointment, which was yesterday. And I like some of the doctors. They try to help me with my problems. But the only thing that I don’t like is that all the options they give me, it doesn’t really help. Besides that, I’m not sure what else to say.*

Excerpt - Document: 129 Respect Interview-cleaned.docx, Position: 980-1978

*And so I know you mentioned the doctors but is there anything when we think about the other staff in medical care so whether it’s the nurses or even the reception staff?*

*S2 03:42 Oh. I have to be honest, I’ve always had a good experience with nurses and any people checking you in and everybody else that I’ve talked to. I don’t really have any complaints about it.*

*S1 04:00 That’s good to hear. All right. And so you said that you’ve had good experiences so far. Could you give me some examples of how they’ve treated you with respect?*

*S2 04:16 For example, the one that I can remember is like when I was in labor, the nurses were very respectful to what I wanted and if I either wanted or not wanted people in the room and not really making any comments even during or after labor and delivery. It was very, very nice and they were really good.*

*S1 04:46 All right. So that kind of being - what’s the word that I’m looking for? - kind of receptive to your needs and your wants.*

*S2 04:56 Yes, yes.*

Excerpt - Document: 129 Respect Interview-cleaned.docx, Position: 2302-3652

*So with this one, can you give me some examples of the sorts of things that might make you feel a lack of respect with your medical care?*

*S2 05:36 Well, I do feel and maybe I’m-- I mean, it’s just the way I feel. And I’m sorry to just kind of bring back again my pregnancy and stuff but that’s been my biggest experience with the hospital and medical care in the state. So I would have to say that I kind of felt a little pressure to have an induced delivery which I was not really-- which I did not want. So I did feel like they were kind of pressuring me too much and at the moment, I was-- well, of course, it was the first time for me having a baby so I was not really thinking and I just felt I got too much pressure into telling me, “You have to do this and you cannot leave,” and that kind of thing. So that was the only thing that I was not very happy with anybody. I just wanted like a little bit of time and maybe like some more time to talk to doctors and that they will kind of sit and tell me more like consequences and all those things.*

*S1 06:59 Okay. So feeling a little bit of pressure and not having the opportunity to kind of start that line of dialogue or the communication that we spoke about earlier.*

*S2 07:10 Yes. I mean, before that I had like-- I don’t really have any complaints but just like for my delivery, I do have some.*

Excerpt - Document: 108 Respect Interview-cleaned.docx, Position: 1039-1237

*When my decisions are honored whether or not the care provider agrees with those decisions instead of feeling like I’m trying to be persuaded in a different direction than what I’ve already decided.*

Excerpt - Document: 108 Respect Interview-cleaned.docx, Position: 2741-3255

*I promised I would ask the opposite question which is if you have any examples of things that make you feel a lack of respect in medical care?*

*S2 06:01 Mm-hmm. And so the two things that came to my mind. One of them was when I was like 15 years ago or something but I had decided not to vaccinate my kids and the doctors were like really-- I mean, made me feel really bad and uncomfortable, and I never wanted to go back to a doctor again because they made me feel like I was making a bad decision for my children.*

Excerpt - Document: 156 Respect Interview-cleaned.docx, Position: 999-1315

*So what is it about those things that makes you feel respected?*

*S2 03:35 I think just that-- I don't know. Maybe that I know myself and that they're aware-- that they're there to help, not just be the be-all and end-all of the answer, I guess. It's more of like a corroboration than just one person making decisions.*

Excerpt - Document: 148 Respect Interview - SP English Translation-cleaned.docx, Position: 939-1477

*Could you give me some examples of the things that make you feel respected by your medical care?*

*S2 02:45 By medical care?*

*S1 02:46 Yes.*

*S2 02:48 The way that the assistants as well as the doctors treat me is always respectful.*

*S1 02:57 Okay. And do you have examples of things that the nurses or doctors at the clinic have done?*

*S2 03:14 For example, at any checkup, before anything else, they first ask me if it’s okay for them to perform whatever procedures may be necessary. They don’t do anything without asking for my opinion first.*

Excerpt - Document: 148 Respect Interview - SP English Translation-cleaned.docx, Position: 1898-2705

*So can you tell me about the regulations that a clinic or medical institution may have? For example, maybe some rules that the clinic or hospital has that might have made you think, “That makes me feel respected,” or something to that tune?*

*S2 04:45 I’m not sure, exactly. All the rules that I’ve seen mention first of all that nothing will be done without-- that’s where they mention, first of all, that everything will be done with one’s consent. They can’t make any decisions if one doesn’t give his or her consent. I think that they take into account the patient’s opinion, first of all.*

*S1 05:12 So, as you’ve said, it sounds like it’s very important that they have your consent and that you have all the information - it seems - before you make decisions. And so that’s important to you?*

*S2 05:24 Yes.*

Excerpt - Document: 168 Respect Interview - SP English translation - cleaned.docx, Position: 3668-4545

*But well, thank you very much for saying that. So, have there been things that your family members or friends have gone through, that they saw, about not feeling respected?*

*S2 06:27 No, just me, but not my family members, no one else. They’ve all been treated very, very well.*

*S1 06:32 Okay. That’s fine, good. Again, besides what you’ve told me, could you imagine something that would make you feel that they didn’t show you respect?*

*S2 06:47 Imagine that they don’t respect me? Once in a while, like no - that is, to imagine, that they don’t show-- like they normally do, that they don’t provide the option of being able to help us. Once in a while: “What do you need?,” [Inaudible](Having the option of) sooner, like once in a while: "No, then call later,” that would be an issue of not [inaudible] (something imaginary, something that to) us, probably, it would be that.*

Excerpt - Document: 106 Respect Interview-cleaned.docx, Position: 4546-5158

*Oh, actually there was one appointment I went to where I was telling my doctor I had pain in my pelvic area and she basically - before I could even answer her - made the decision for me that I did not want to get a pelvic exam done at this time, and that if in the future I want to let her know. Before I could even be like, "Yes," "No," or "Wait, why do we need to do that?" she was just like, "But you just seem like you don't want to do that right now, so if you do in the future just let me know and I can schedule something for you." So I felt like she just made decisions for me without letting me answer.*

Excerpt - Document: 160 Respect Interview-cleaned.docx, Position: 1246-1617

*So thinking about maybe some of the other people that you interact with when you do go into the clinic or to see a doctor, so thinking about maybe the intake nurses or the intake staff, can you think of anything with your interactions with them that make you feel respected?*

*S2 04:52 Yeah. This is what I'm saying; respect me as taking as an active role on my own health.*

Excerpt - Document: 160 Respect Interview-cleaned.docx, Position: 2332-2776

*So if you're to kind that just think hypothetically, say that you were to go in for an annual physical or something like that, can you think of anything that the staff or the nurses or the doctor could do that would really kind of rub you the wrong way or something that you wouldn't like or perhaps a way that they have of interacting that wouldn't really be your preference?*

*S2 07:12 Yeah, if they were pushing their personal opinions on me.*

**Other**: Another aspect of respect in medical care not covered by any other codes

Excerpt - Document: 101 Respect Interview-cleaned.docx, Position: 4181-5608

*So could you give me some examples of the sort of things that might make you feel a lack of respect in your medical care?*

*S2 08:26 Pretty much just health insurance [laughter]. That’s about the main one that kind of kills me. I mean, I don’t have any problem with any of the doctors. It’s just cost. Years ago, I almost cut off my thumb on a table saw, and it was a big question of whether I wanted to go in and pay $600 to get it stitched up or if it would heal on its own.*

*S1 08:54 Yeah, okay. So that health insurance aspect is kind of the big factor when it comes to a lack of respect and what--*

*S2 09:11 Yeah. I mean, it’s not really respect for the health insurance-- I mean, not really respect for the health industry. It’s more a question if it’s worth me dealing with the pain or not, and it’s just not wanting to go in and having to figure out how to pay for it.*

*S1 09:31 Okay. So would you--?*

*S2 09:32 So, I mean, I make just enough money to where I can’t afford [inaudible] health insurance, but I make too much where I can’t be on Medicaid. So it’s kind of one of those where I’m kind of in the middle there where it makes it really tough.*

*S1 09:57 Yeah, yeah. So that seems like it’s-- it kind of sounds like it’s one of those policy issues where perhaps it’s a lack of respect for certain people who kind of fall in between what they can afford and what Medicaid is set up for. Is that right?*

*S2 10:18 Yeah, yep.*

Excerpt - Document: 150 Respect Interview-cleaned.docx, Position: 3506-4191

*And then policy-wise, is there anything that you can see as having or showing a lack of respect?*

*S2 08:09 Yeah. There are some policies or guidelines that they have to do for everyone. And it felt just-- I guess I have to say an example, but I don't know how to say it. But when you get pregnant and you go into the doctor, like, "Okay, well we need to test you for all these STDs." And like, "Well, no. I'm not going to be-- why would I be tested for STDs? I'm married." And like, "Well, half of all pregnancies is not with the person they say." Like, "Well, that's not the case here." And it just felt like a very silly policy to have for someone who doesn't at all conform to that.*

Excerpt - Document: 172 Respect Interview - SP English translation - cleaned.docx, Position: 1192-1374

*Could you give me some examples of things that make you feel respected in your medical care?*

*S2 02:54 One’s privacy and the communication they keep up with you. The trust one feels.*

Excerpt - Document: 110 Respect Interview_cleaned.docx, Position: 1727-2039

*And what about the medical institution itself, say the clinic or maybe the larger healthcare network? Is there anything that these institutions can do that show respect?*

*S2 05:06 Making sure that they are up to date on the education for treatments and the symptoms, or, I don't know, causes for things, I guess.*

Excerpt - Document: 161 Respect Interview-cleaned.docx, Position: 6883-13643

*what I'd like to do is kind of take that broader question of respect and kind of flip it around. So could you give me some examples of the sorts of things that might make you feel a lack of respect in medical care?*

*S2 11:50 Well, not so much a lack of respect but kind of more like the [inaudible]. I think, for example-- and I don't know. Well, I think it's really different because we're talking about respect. I've seen situations where it's gotten out of control, and it's been very disrespectful, and it's kind of interesting. It just depends on the patients as well. But it's been really disrespectful. A lot of times, and it does happen in hospitals where patients are really disrespectful to many staff. So that's really wow. And you try to help them out as much as you can, but it comes to those situations where they're really, really disrespectful. Either they say bad words. Either they don't follow through with their [indications?] some of their medical staff is recommending to them. I don't think they [inaudible].*

*S2 13:03 What the medical staff want is to get the patient well. They want the patient to be well, so they do a lot of recommendations, right? So if the patient doesn't follow through and things like that or someone has to call, then a lot of the patients get really angry. And do you expect them to listen to anyone? No. That occurs. So it's like, "Wow." [inaudible] but getting that good care. And it's the opposite of, yeah, the patient being good, just respectful to the staff. And I don't know if this could be the situation, I guess, that patients go through. But yeah, I've seen that opposite side. And then the last time I got disrespected [inaudible]. I think we do try our best to have our patients be content and have the care that they need and hear them out and get them waiting to be-- I mean, the patients come first. They really need to take care of them. But yeah, of course, you get what's in the [inaudible] and out there. So I've seen them too. But yeah, I am speaking more towards not the patient's part of it, but I think it's more of a [tangent?] [inaudible]. I think I'm getting out of that spot.*

*S2 14:49 Insurances, it's actually like insurances. When patients come in and their insurance is not contracted with the hospital, and they'd say, "Oh, my goodness," it's really hard. And especially when you don't have no insurance at all, and they get referred to the department of enrollment services where they do get the help. Actually, hospitals do have enrollment services departments where they help patients get coverage, whether it be a copay or a minimal copay. That's something just to cover the medical expenses. And that is really-- well, it's really interesting to see when a patient doesn't qualify for medical insurance or help because they're over budget or they're over-income. That really interests me because patients, they're in the middle. They get stuck in the middle where they don't have insurance. They can't go to their doctor's appointment because they don't have insurance. And of course, if they go, they get bills with this huge amount of money. So that's something that's really interesting, and that's really hard to see, too, because you do want to help them out. You do want to get them the care that they need. But because of that insurance that is blocking their way to care, that's really messed up. But then again policies and procedures, and it's like, "Oh, my God." I've seen a situation where-- and I have to see that because we try so much to help them out. A patient had to leave her job just for her to get coverage.*

*S1 16:40 Oh, wow.*

*S2 16:40 That is really messed up because her job is supposed to be offering her some sort of insurance or coverage if she's working 40 hours. If she's a full-time employee, then she should be getting a coverage or some sort of benefit from her job. Oh, my God, I was in shock when I heard that. And she wasn't able to get the care that she needed because of it. She wasn't able to qualify for anything because she was over-income. And it's not even that much because there are brackets from where patients qualify for the discount programs or Medicaid or things like that. There's procedures or brackets that patients need to have or the amount of money that they need to earn kind of thing. And I don't know how they calculate that, but I've heard about it. And when they're over that bracket, or they don't quality within that bracket, they don't qualify for anything. Oh, my God, so that patient was in that situation where she had to leave her job just because of it. And then after she left her job, she qualified for the discount program, more through [inaudible]. There should be more help in that sort of area of insurances for patients that are uninsured that they would have the help or the resources to get insurances like a normal person that works full time that has coverage from their own job. But we do have patients that don't get coverage. They don’t have insurance from their job. They have to rely on the discount programs or Medicaid to help them. And that's most of that Medicaid is-- but we have Medicaid, and we have discount program. But we have the separate part of this where uninsured patients that are underserved because that really helps them out.*

*S2 18:36 But then there are some situations that they could qualify, and that's been messed up. That's been pretty messed up quite a bit. When they qualify for them, it doesn't work. We tried so much to help them out. But then once that happens, there are situations where-- and I've also seen grants, like the grants the hospitals offer for patients. For example, breast cancer, there's grants in the hospital that come from [inaudible] or other foundations, nonprofit foundations that come in and help the patients out with their copays, which really awesome. Those organizations are really helpful for the hospitals to have because they help those patients get the care [inaudible] or schools and even follow-up appointments with the oncologist. That's really awesome. But then it really sucks because when the funds run out, then the patient is left with-- there's no funds anymore. You're going to have to cover your tests, the visit. And it's like, "Great." They have to go through the process again of going through enrollment services and getting coverage with the discount program," and that is really wow. So I've seen lots and lots of the insurance part of what patients go through just to get that coverage, just to get their medical care. And it's kind of something that I wish our government could do a bit more on and be more aware. But then, again, that's tangent to the whole thing, but I thought that was something important, so.*

Excerpt - Document: 129 Respect Interview-cleaned.docx, Position: 14617-15404

*And if we think about the study materials that might be used with this hypothetical study like flyers or brochures or emails, can you think of any ways that they could create these materials in a way which showed respect?*

*S2 23:59 Well, again, and of course they don’t do it like using names, which would not be fun, or using your medical record number. I would find that very disrespectful if they did without a consent. And also I don’t think it would be necessary. I mean, for statistics, it would not be necessary. I mean, I understand that maybe racial stuff like either coming from certain backgrounds will make people more likely to get certain diseases or not. So I understand that and I don’t feel offended by that but any other personal information would be very disrespectful.*

Excerpt - Document: 108 Respect Interview-cleaned.docx, Position: 3962-4686

*Are there other things—actually, I guess you said that both of those examples made you less interested in engaging with medical care going forward. I’m curious if you could say a little bit more about that.*

*S2 07:51 I mean, in general, I guess I don’t have a super high opinion of most medical care providers. I mean, it’s not necessarily the individual but I just feel like in our culture, it’s really driven by money a lot and by insurance and that people aren’t always getting the best care because either the care providers are overworked. They don’t have enough time to really sit and listen to what’s going on to really know who they’re dealing with. It’s just sort of in and out, and so-- what was the question again?*

Excerpt - Document: 126 Respect Interview-cleaned.docx, Position: 2515-3382

*what sorts of policies that the medical institution has in place that would show respect. So you kind of jumped to my question there a little bit, but [inaudible]--*

*S2 06:29 Yeah, okay. So I guess to address the administrative part, I just feel like just healthcare, in general, is really focused on cost cutting and finances, and that just seems, in a lot of ways, to be not conducive to making patients feel respected. Those two goals seem to be at odds in a lot of ways. Because, I mean, if a hospital’s focused on cost cutting, they’re going to really try to limit the amount of time that providers spend with patients, for example. So that’ll cut into how much time I have to spend with a provider, and that’ll affect how the provider handles our consult. If they’re under time pressures and-- yeah. I mean, that could affect a lot of things about the encounter.*

Excerpt - Document: 126 Respect Interview-cleaned.docx, Position: 3560-4267

*what sorts of things make you feel a lack of respect when it comes to your medical care? And I know you touched on a couple of them, so if there’s anything else that comes to mind.*

*S2 07:57 I mean, I’m sort of just thinking about how the healthcare system, as a whole, is just such a big machine, that it’s really hard to feel recognized as an individual. It often feels like I’m just kind of like a number in a lot of my interactions relating to healthcare with all kinds of medical staff. And I think that is kind of a reflection of those larger cost-cutting pressures and other pressures that are coming just along with being involved in such a large machine. So, yeah, that’s sort of another part of it.*

Excerpt - Document: 156 Respect Interview-cleaned.docx, Position: 418-915

*So could you give me some examples of the sorts of things that make you feel respected in your medical care?*

*S2 02:39 I think being listened to, in general, not being talked down to. I find that doctors who can appreciate somebody doing their own research on something, as long as it's not bad research, and accepting that information as something that could possibly actually be true and not just looking over it as a joke almost, I think those are the biggest things that make me feel respected.*

Excerpt - Document: 158 Respect Interview - SP English translation - cleaned.docx, Position: 2497-2977

*Now, what can you tell me about the policies that the medical institution has? For example, are there certain policies that show you respect? That you could think of--*

*S2 05:29 Yes. Well, being that all the clinics have their policies, such as not using the telephone, not turning it on when you arrive to register for the appointment. What else do I remember? You know, what many of them do, no smoking inside, all of that. And, for me, it seems good that it’s that way, really.*

Excerpt - Document: 148 Respect Interview - SP English Translation-cleaned.docx, Position: 1898-2705

*So can you tell me about the regulations that a clinic or medical institution may have? For example, maybe some rules that the clinic or hospital has that might have made you think, “That makes me feel respected,” or something to that tune?*

*S2 04:45 I’m not sure, exactly. All the rules that I’ve seen mention first of all that nothing will be done without-- that’s where they mention, first of all, that everything will be done with one’s consent. They can’t make any decisions if one doesn’t give his or her consent. I think that they take into account the patient’s opinion, first of all.*

*S1 05:12 So, as you’ve said, it sounds like it’s very important that they have your consent and that you have all the information - it seems - before you make decisions. And so that’s important to you?*

*S2 05:24 Yes.*

Excerpt - Document: 133 Respect Interview-cleaned.docx, Position: 6986-7713

*But can you imagine anything that might make you feel a lack of respect truly or maybe something that you can imagine happening to your friends or family?*

*S2 09:38 Yeah. Insurance, I think, plays a big part. I know a lot of my family members, the older generation, who have come to the United States, a lot of them are on Medicaid. They have barely grade-school education and barely write their names, let alone speak the language. And when you go in to see a provider and them not giving you the time or the full treatment, would be totally disrespectful, I would think. And not treating them fairly or not treating them like they would another person who spoke the language, I think would be a total disrespect in healthcare.*

Excerpt - Document: 133 Respect Interview-cleaned.docx, Position: 8323-8860

*So can you think of any policies, maybe, that a medical institution might have that would show a lack of respect?*

*S2 11:51 Yeah. If they capped certain number of patients being seen with that specific type of insurance, I think that would be really disrespectful. I know state and federal insurances don’t-- or Medicare and Medicaid don’t take quite as much as, say, [inaudible] insurance by putting a top on that so that they can restrict the access, I think would be really disrespectful to them as humans, to deal with medical care.*

Excerpt - Document: 133 Respect Interview-cleaned.docx, Position: 27455-27749

*So what about medical care in general? You gave that a five.*

*S2 09:33 Yeah. Again, I work in medicine. I work in the health care system. So I would say my personal results are doubled by professional-- I would say I trust medicine because I work in medicine. So I would say I trust in general.*

Excerpt - Document: 131 Respect Interview-cleaned.docx, Position: 5122-6729

*Great. And can you think of anything from a policy side that might show a lack of respect?*

*S2 09:57 A policy side, so I don’t know if this true. But I have heard, in the past, from others that a lot of times, there’s a policy - and I don’t know if it’s the healthcare providers or if it’s required by the insurance companies either way - of asking about specific things such as weight management, like, “Did you mention their weight this time? Did you mention this this time,” certain things that they have to kind of check off the box. So I feel like sometimes that policy is kind of annoying. It’s like, “Well, no, I twisted my ankle. And I just need you to fix my ankle. I wasn’t here to talk about BMI this week.” So I think if there are policies like that, that you have to discuss every single item at every single appointment, it gets discouraging, I guess [laughter], to the patients.*

*S1 10:58 Okay. So what I’m hearing is sort of these mandatory questions, whether it’s the institution or the providers, aren’t so relevant to the issue at hand. Is that right?*

*S2 11:18 Right.*

*S1 11:19 Okay.*

*S2 11:20 Yes. And I understand. If they take your blood pressure and it skyrocketed, maybe you should have a discussion, of course. I broke my toe recently, for example, And I had to go urgent care. And my blood pressure was crazy high because I was in a lot of pain. They’re like, “Why is your blood pressure so high?” I’m like, “I know because my toe is bent the wrong way. And I’m in a lot of pain. Can you please help me fix my toe [laughter]? I will talk about blood pressure after I have calmed down.”*

**Privacy**: Privacy or confidentiality protections

Excerpt - Document: 150 Respect Interview-cleaned.docx, Position: 1294-1643

*S1 04:35 All right. And so as far as the policies that the medical institution has in place, are there any that you think are respectful or are good for kind communicating respect?*

*S2 04:58 Yes. I mean, HIPAA policies are certainly great. I love privacy, and that’s actually a lot. Though I don't really know of any specific other ones, though.*

Excerpt - Document: 154 Respect Interview-cleaned.docx, Position: 2012-2166

*are there any policies that the medical institution has in place that you think show respect?*

*S2 04:58 I don't know. I mean, I think HIPAA's a good thing.*

Excerpt - Document: 172 Respect Interview - SP English translation - cleaned.docx, Position: 1192-1374

*Could you give me some examples of things that make you feel respected in your medical care?*

*S2 02:54 One’s privacy and the communication they keep up with you. The trust one feels.*

Excerpt - Document: 161 Respect Interview-cleaned.docx, Position: 4481-6842

*And then thinking a little bit more broadly now, so the medical institution and the policies that it has in place, are there any policies that kind of jump out at you as being respectful?*

*S2 08:34 So the privacy, HIPAA, I know there's privacy. Yeah. That does help a lot with-- protects your information. That thing is not going to be shared with anyone else without your permission. Of course, I know that sometimes providers do need to share patients' medical information with other hospitals or providers, but it's within hospitals, providers. If not, they're sharing it with other people who are not authorized to do so. So HIPAA is a thing that the hospitals have. And yeah, that's really helpful to have. Outpatient consent forms, same thing. You are consenting to being seen and treated at the hospitals. But then again, it's there for information. It's specific information for them to share it, whether it be for the financial side, the medical side also. But then again, there is those policies in place for that in order to protect our information and then just share it with those that need to have that information. So yeah, that's really helpful to have. And then, of course, there's the other ones like the power of attorney forms the hospitals have where certain family members are authorized to have specific information for their patients. So yeah, I mean, all of that helps to have in our hospitals. Yeah.*

*S1 10:13 Okay, great. All right. So now with the--*

*S2 10:20 And also with--*

*S1 10:20 Oh, go ahead.*

*S2 10:21 Sorry. And I did remember something else too. Even employees that get taken care of-- employees that work in a hospital. And when they get sick, we also get noticed and respected because there is-- we get treated with respect as a patient, too, and respect our information. Even though they know that we're actual employees from the same hospital, we also get treated with respect the same way a patient would. So it's across the board, across the board, so that's really awesome too.*

*S1 11:03 Okay. Interesting. There's this kind of a-- oh, boy, how do I explain this? This idea of equality regardless of not just--*

*S2 11:18 Yeah. So it doesn't matter if you're a patient or an employee and even if you work there. At the hospital, you're treated with the same respect and courtesy across the board, and that's really awesome.*

Excerpt - Document: 153 Respect Interview - SP English translation - cleaned.docx, Position: 2955-3359

*are there certain rules at the clinic that make you feel respected?*

*S2 05:39 Yes. They always tell me that everything will be in private, that my name will never be exposed in any case of something. Yeah, there has never been any reason for me to feel like they haven’t respected my privacy.*

*S1 05:59 Okay. It sounds like, for you, having privacy, that’s pretty much like being respected.*

*S2 06:05 Right.*

Excerpt - Document: 122 Respect Interview-cleaned.docx, Position: 4252-5177

*And was there anything kind of on the institution side or policy-wise that you think could show a lack of respect?*

*S2 03:06 No. I actually don’t because I think that the HIPAA law-- I mean, I understand why it’s in place that it actually really kind of bothers me especially when you’re talking about your children and stuff, and I feel like-- sometimes release of information, especially when I’m dealing with parents who are trying to put their children for [inaudible]. I’m like, “Well, I’m sorry. You don’t have a release of information.” It’s that part that’s frustrating to me because I feel like if you are a parent and you’re paying for your child’s care-- yeah. The child should be able to have some privacy and maybe something that they’re being treated for but when the parents financially responsible and everything else, I don’t feel like the child should have I guess as many roadblocks for a parent. So, yeah.*

Excerpt - Document: 123 Respect Interview-cleaned.docx, Position: 1662-2125

*I’m curious if you can think of anything, any sort of policies that a medical institution might have that would show respect?*

*S2 04:15 I think compliance, I suppose they’re respectful, my health care and probation should be private and so when they honor that, whether it’s calling my name back to take me back to the office where they keep my chart. And even when you’re picking up prescriptions, just being mindful of my privacy. I think that that’s respectful.*

Excerpt - Document: 142 Respect Interview- cleaned.docx, Position: 462-1181

*So with that in mind, can you give me some examples of the sorts of things that make you feel respected in your medical care?*

*S2 03:02 Definitely having that one-on-one and having the feeling that they're listening and actually making that eye-contact with me. And I know, initially, when I first have a new physician or nurse practitioner, it's hard to have that trust right away. So I would say, just having that care and sympathy towards me and the time, not feeling like I'm being rushed during my assessment with them. Yeah. So asking any pertinent questions and making me feel like my information is not shared with other people, and that I can feel comfortable with them. So maybe that reassurance as well. Yeah.*

Excerpt - Document: 142 Respect Interview- cleaned.docx, Position: 2293-3064

*can you think of any policies that a medical institution has in place that could show respect?*

*S2 05:38 [inaudible] what?*

*S1 05:39 Can you think of any policies that a medical institution maybe might have that show respect? We talked about people a bit and how they can show respect. And I'm wondering if you might have any examples of policies that make you feel respected? One thing for example, that made me kind of think about that, was you said the information was private. Anything else like that? Any other policies that might make you feel respected?*

*S2 06:08 Just if the HIPAA is definitely respected, knowing that my information won't be shared with other people. Other policies? Would you be able to provide examples? Or is that something that's not allowed?*

Excerpt - Document: 142 Respect Interview- cleaned.docx, Position: 7839-8723

*Can you think of any policies that feel disrespectful to you in medical care, whether you've experienced any or whether you can just imagine any?*

*S2 13:42 Yeah, I guess there's also been leaks of private information. And so even though we have the HIPAA, I feel like there's been instances, even where I work, where there has been in the past some scam that had an information leak. So even when people promised that their information won't be exposed, I feel like it's often overlooked. And so we don't realize, even just from verbal communication, it's easy to talk about other patients and that information gets easily revealed.*

*S1 14:24 Okay. So maybe that there aren't the strictest policies in place to keep patients' information confidential. Okay.*

*S2 14:34 Right. Yeah, I feel like they have the idea of making sure that information is private, but it's not strictly enforced.*

Excerpt - Document: 156 Respect Interview-cleaned.docx, Position: 2230-2458

*Can you think of any policies that show respect?*

*S2 05:48 I mean, like the normal one, like the doctor-patient confidentiality. I think that's respectful. I don't really know of any other policies that I'd be able to comment on.*

Excerpt - Document: 158 Respect Interview - SP English translation - cleaned.docx, Position: 1200-1515

*Could you give me some examples of the things that make you feel respected in your medical care?*

*S2 03:21 I like it a lot, and I go to my clinic because they respect my schedule, they respect my privacy. I’m at ease because they serve me well, they are respectful to me. I’m comfortable, due to many things, really.*

Excerpt - Document: 128 Respect Interview - SP English translation - cleaned.docx, Position: 2650-3611

*Can you think of things about the policies that a medical institution might have? For example, is there something that the clinic does, with certain rules, or, as I said, certain types of policies that make you feel respected? Or can you think of an example, or something along those lines?*

*S2 06:48 You mentioned policies; let me think about that. Well, this hasn’t been the case for me, but I’d like to think that our information is private, confidential, and that they can send that information only for our own safety and for the sake of our children and of our families. They should really maintain that privacy because that worries me, when I get calls from these phone numbers and I wonder, “Could that be from the clinic? Where would they be selling our information?” and we get these anonymous calls from telemarketers, of course, but sometimes I think, “Could it be that they sell the information?” and every day those telemarketers are bothering us.*

Excerpt - Document: 174 Respect Interview - SP English translation - cleaned.docx, Position: 1749-2251

*Okay. And, what can you say about the policies that a medical facility has? Do they have some rules or policies that show you respect?*

*S2 04:20 Well, I don’t know. I believe so.*

*S1 04:25 Okay. And can you think of any? Or…*

*S2 04:31 Well, usually, always, they make sure they are attending to the right person, because they always ask for my-- that's why they’re always sure of my date of birth, my full name. The receptionists always ask for my ID.*

*S1 04:50 Of course.*

*S2 04:51 I mean, all that.*

Excerpt - Document: 116 Respect Interview-cleaned.docx, Position: 433-990

*So could you give me some examples of the sorts of things that make you feel respected in your medical care?*

*S2 02:58 I think, so far - knock on wood - I haven’t had to get much medical care lately. But when I have, I like-- I’m trying to think here. I guess the big things for me would be consideration of privacy and their timeliness on how they get back to me about results on stuff if I have any tests done or whatnot. And just giving me as much information as possible about any questions I may ask about medical issues that I have, that sort of thing.*

Excerpt - Document: 116 Respect Interview-cleaned.docx, Position: 1403-2029

*So if we kind of take a broader look at the medical institution itself, whether it’s the clinic or the health care organization that you receive care from, are there any sorts of policies that, in your mind, show respect for patients?*

*S2 04:55 Yeah, just like patient privacy, like HIPAA, that sort of thing. Not being able to disclose things to family members or friends or whatnot without permission from the patient, that sort of thing. I’m not really aware of many of their policies outside of that, but I know those are the main ones, the privacy and not being able to disclose information without consent of the patient.*

Excerpt - Document: 113 Respect Interview_cleaned.docx, Position: 2044-2719

*Can you think of any policies that the healthcare system, for instance, might have that might be-- or that you can think of that would be respectful?*

*S2 05:08 Well, I work for [the healthcare system] and one of the things that I find very respectful for even my nurses, the release of information and the privacy act that we utilize for our minor patients. Being a parent of teenagers, I actually do appreciate that. That way it gives the kids the opportunity to have a conversation with the provider, knowing that their conversation will be private. And that way they get the help they need, especially if they're trying to keep it away from their parents or family members.*

Excerpt - Document: 143 Respect Interview-cleaned.docx, Position: 2548-3338

*And then if you were to think of the medical institution, whether that’s the clinic itself or maybe the whole healthcare system, excuse me, can you think of any policies that might show respect?*

*S2 [01:08] Well, the general policy of not discussing your issue until verifying it’s the right patient is nice. Let’s see.*

*S1 [01:24] So that privacy and confidentiality aspect?*

*S2 [01:27] Yeah. Just double checking you’re talking to the right person before just diving in to any personal stuff. What else? I mean, I do appreciate the small talk that happens on the way to the-- when they’re bringing you to the doctor’s office from the waiting room. Like, “How was your day?” and all that. And those conversations, they can help to relieve the stress that you’re having [inaudible]. Yeah.*

Excerpt - Document: 131 Respect Interview-cleaned.docx, Position: 401-1392

*So could you give me some examples of the sorts of things that make you feel respected in your medical care?*

*S2 02:44 When I feel like the doctor has listened to my concerns thoroughly. I’ve had experiences with providers where I’ve felt like switching providers. Because I would go in with a complaint and it might be something random. And they’re like, “It’s probably because you need to lose weight.” That’s like, “Well, yeah, that’s one thing. But can you listen to what I’m actually talking [laughter] about?” So staying focused on what I’m there for and feeling like I’m being listened to is the biggest thing.*

*S1 03:20 Okay.*

*S2 03:22 And then just feeling like what I say is held within confidence, of course. So I would say those are the main ones.*

*S1 03:35 Okay. So yeah, the first one that you brought up was this issue of-- or not necessarily an issue. But this idea of communication, would you say that that’s kind of the most important element for you?*

*S2 03:58 I think so, yes.*

Excerpt - Document: 131 Respect Interview-cleaned.docx, Position: 2163-3044

*And to kind of think about this in an even broader sense, if you think about the medication institution itself, whether that’s your healthcare system or this clinic, can you think of any policies that these institutions might have that can show respect?*

*S2 05:48 Well, I don’t know of any policies, specifically, if that’s what you’re asking. I’m not sure if I understand the question entirely.*

*S1 06:03 Or is there any that you could-- if there’s anything that you can think of that might be a good policy for maybe some clinics and healthcare systems to implement.*

*S2 06:15 Gotcha.*

*S1 06:16 Yeah.*

*S2 06:17 Yeah. Let’s see. Well, I think good policies would be returning phone calls in a timely fashion during business hours, making sure that confidentiality is a high priority. In general, having good training and support for their team so that everybody is happy in their jobs.*

Excerpt - Document: 145 Respect Interview-cleaned.docx, Position: 1858-2076

*And then to kind of broaden the question out a little bit, are there any policies that the medical institution has in place that you find respectful?*

*S2 04:51 If you're following HIPAA guidelines, thank you [laughter].*

**Provider/staff attitude**: Provider/staff attitude with patient; quality of interaction; treating patient like a human; putting in effort to know patient; listening to and acknowledging patient’s concerns and goals vs. ignoring or dismissing patient or making assumptions

Excerpt - Document: 127 Respect Interview - SP English translation - cleaned.docx, Position: 706-954

*could you give us some examples of the things that make you feel respected when you receive medical attention?*

*S2 00:16 Yes, well, since I speak very little English, I like to be-- they find me a translator and everything; that makes me feel good.*

Excerpt - Document: 127 Respect Interview - SP English translation - cleaned.docx, Position: 1037-1154

*So what can you tell me about individual medical providers or staff?*

*S2 00:44 Well, yes. They’re also very friendly.*

Excerpt - Document: 127 Respect Interview - SP English translation - cleaned.docx, Position: 1174-1582

*And so can you think of other examples? That maybe specific people have done something to make you feel respected.*

*S2 01:06 Well, yes. I mean, the same as I mentioned, right? They make one feel comfortable, trying not to make them wait longer, speaking-- even if they don’t speak English to me, by trying to speak a little Spanish or if not, by finding a translator. But yeah, one feels that. I feel pleased.*

Excerpt - Document: 127 Respect Interview - SP English translation - cleaned.docx, Position: 2387-2515

*Where there other moments you can tell me about?*

*S2 02:41 Well, well, this in particular is all that I like. Mm-hmm. I mean, no.*

Excerpt - Document: 127 Respect Interview - SP English translation - cleaned.docx, Position: 3161-3962

*So since you don’t have examples, what about if you could imagine for yourself something that would make you feel disrespected or that your friends or family members have been disrespected? If you can, think of examples. Or something that just wouldn’t make you feel respected.*

*S2 04:10 Yes. One example is that my [family member] had this pain and he went to the clinic and they told him that they were going to run some tests on him and, well, they never called him to-- they were going to call him to make an appointment in another clinic and they never called to schedule it. He called them and they told him to wait because they had said they would call him, but they never did. Those are things that don’t--*

*S1 04:32 Yes. Right, that --*

*S2 04:34 --that wouldn’t be good. But that’s not the case.*

Excerpt - Document: 127 Respect Interview - SP English translation - cleaned.docx, Position: 4213-4888

*So what can you tell me about what the individual medical providers or staff could do to demonstrate a lack of respect for someone in their clinic? Let’s suppose here.*

*S2 05:17 Well, maybe with a young girl and a doctor asking her other types of inappropriate questions; I imagine that that would be a lack of respect.*

*S1 05:25 Hmm. Mm-hmm. Okay. No, it’s fine, it’s good.*

*S2 05:30 But that’s not very common.*

*S1 05:31 Yeah, that’s a difficult one.*

*S2 05:34 As I’ve said, that’s not the case with me, so I wouldn’t know what to comment since, well, I don’t know how. More or less that would be one idea, but, I mean, no. I haven’t yet lived that experience, so I don’t know.*

Excerpt - Document: 127 Respect Interview - SP English translation - cleaned.docx, Position: 5000-5787

*Okay, so what can you tell me about the policies that a medical institution might have in place? For example, as I mentioned to you previously, what types of policies would demonstrate a lack of respect? Let’s say, something that the institution or clinic might do or not do that would demonstrate a lack of respect.*

*S2 06:23 Like, maybe, a lack of respect would be to not help other people who come from countries where neither Spanish nor English is spoken, because we’ve seen a lot of people who come here, for example, from Guatemala where there are other languages and no, I mean, it’s very difficult to obtain medical attention for them.*

*S1 06:52 Uh-huh. Okay.*

*S2 06:53 And it would be a lack of respect to look down on someone else or give them a dirty look. Yes, that [laughter].*

Excerpt - Document: 114 Respect Interview-cleaned.docx, Position: 2003-2924

*Can you think of any experiences you’ve had where you felt, “I feel really respected in this moment in medical care?”*

*S2 05:08 Yeah. Actually with my primary care physician. She is extremely good at listening to me and asking the right questions and leading me to-- basically, I several times come in and say, “I think this may be the issue and she doesn’t discount what I say. She will think and say, “Okay. We’ll check it out if that may be the issue.” If she doesn’t believe it’s an issue then she’s like she’ll explain it very clearly as to why it shouldn’t be something I worry about. I feel not trying to talk above my head basically. She has learned basically where I am as a person, how I am able to communicate, and so I feel like she’s put in that effort to know me as a patient. And so because of that, I really enjoy being with her and I feel like she respects me as a person and not just as another patient.*

Excerpt - Document: 114 Respect Interview-cleaned.docx, Position: 3153-3829

*Can you think of any ways that a medical institution could have, any sort of policies that a medical institution might have that would show respect of show lack of respect?*

*S2 07:01 Well, that’s a good question. I guess, it sort of depends on what aspects we’re talking about. Personally, I’m not entirely sure but in thinking about myself, my family, and things that I’ve seen happen with friends and family in medical care. Being sure and having policies I suppose to make sure that if someone is-- if a doctor is able to keep his or her moral standpoint out of his medical practice. It’s especially related to youth. I don’t believe they have any place in medical practice.*

Excerpt - Document: 114 Respect Interview-cleaned.docx, Position: 4928-6829

*And this sort of goes to a couple of the issues that you’ve brought up but I wanted to ask it explicitly. Have you ever felt the lack of respect in the medical setting based on who you are, what you look like, what you believe, any of those sorts of traits?*

*S2 10:23 In a study?*

*S1 10:24 In medical care or in a study, I guess, either way.*

*S2 10:28 This is actually my first study but in medical care, yeah, I have felt sort of ignored. My concerns are kind of brushed off and when I was bringing certain things to the forefront saying, “Hey, I think this may be what my issue is,” they kind of just brushed me off and went, “Well no. I know that’s not a problem. This isn’t this.” They didn’t do any tests. I mean, there was plenty of times that I left the ER after experiencing severe stomach pain and they’re just like, “Well, we don’t know what the issue is. Go home.” And of course, that was when I didn’t have insurance. I felt that when I didn’t have insurance, I was basically just, I guess, a burden to them at that point without a doubt. I ended up with $6000 of medical bills because I had to go back into the ER multiple times to get a skin infection taken care of. The first time I actually went into the ER, I had gone in with a temperature of 106 degrees and I waited in the empty waiting room for four hours in the morning. And when they brought me back, my temperature had already dropped and when I told them what it had been, he’s like, “Really, are you sure you didn’t read it wrong?” So I was a little upset. But then they also didn’t run a proper test and so they didn’t find out what it was that was giving me the infection so they didn’t give me the proper antibiotics.*

*And then the next time they gave me the wrong antibiotics that didn’t have good kidney penetration so it wasn’t getting rid of the kidney infection. So I felt there was a lot of oversight in that instance.*

Excerpt - Document: 114 Respect Interview-cleaned.docx, Position: 20493-21173

*And can you say a little bit about why you answered these questions this way?*

*S2 32:54 Well, for the medical, I’ve had my own personal bad experiences but for the most part all my experiences thus far since-- I suppose probably after sort of educating myself on the things that are going on with my own body, I’ve been able to communicate well with my doctor and things have gone very well since then. As per the study, I very much felt listened to, things have gone very quickly, very smoothly, and I don’t feel at any point like-- I mean, I wasn’t made to feel stupid if I didn’t know something. If I didn’t understand something, I could have it explained clearly and concisely.*

Excerpt - Document: 114 Respect Interview-cleaned.docx, Position: 21190-22239

*And then on a scale of one to five, and going back to that same scale, how much did you stay you trust medical care in general?*

*S2 34:11 It’s kind of a difficult question because it kind of comes back to that depending upon the doctor.*

*S1 34:17 Right, right.*

*S2 34:22 For myself what I’ve experienced in my recent years, I would say a five. But concerning family members in the last two years, I would say honestly two. Things went very, very poorly with my father-in-law with esophageal cancer.*

*S1 35:07 I’m sorry to hear that.*

*S2 35:10 Yeah. And family friends who were going to the same doctor have also either passed or have had basically nothing done for them. So I’d say an instance for that doctor, it’s a very low number. He didn’t listen, he people feel stupid, and never explained anything. And generally, I felt he wasn’t as aggressive with his treatment as he should have been.*

*S1 35:57 Okay. So it sounds like there’s a lot of-- like trust sounds like it’s a really personal dependence on the individual person and an individual doctor.*

Excerpt - Document: 101 Respect Interview-cleaned.docx, Position: 1119-2477

*What about the other medical staff? How could they show respect?*

*S2 03:50 I think nurses and them show quite a bit of respect, but just taking the time to actually talk to you and explain what they’re doing instead of just, “Sit and hold out your arm.” And actually tell you what they’re doing, if they’re taking your blood pressure or whatever, just explaining what’s going on and sharing. I’ve had nurses in the past where they don’t share what your blood pressure is or share what your heart rate is and actually take the time to tell you your whatever over whatever on your blood pressure and tell you if that’s decent, good, or something like that, so. Also, the nurses nowadays seem to be doing quite well with that, with letting you know what actually they’re doing and what they’re seeing and what they’re recording.*

*S1 04:54 Okay. So what I’m hearing is that you really appreciate kind of the ongoing dialogue and the back and forth of information and not so much them kind of just taking the information and not really filling you in.*

*S2 05:14 Right. And I mean, if they’re sharing with you, then they’re treating you like they respect you, that you’re capable of understanding what they’re talking about. When they used to keep it from you, it kind of felt like they were above you and that you wouldn’t understand what they were doing anyway, so.*

Excerpt - Document: 101 Respect Interview-cleaned.docx, Position: 2498-3444

*And then, so what about the policies that the medical institution has in place? What kind of policies do you think show respect?*

*S2 06:03 I mean, I think now-- I mean, recently, I had [surgery], and I didn’t have insurance. So with them being able to actually still go in and do what they needed to do and not treat me, yeah, like a leper, pretty much, because I didn’t have insurance and still do everything that they would’ve normally done, that was pretty great. I was pretty freaked out when I had to go in and didn’t know what was going to happen since I didn’t have insurance at the time. So that policy of being able to still take care of the patient and take care of what they needed to do. And then I had people come in and help me get Medicaid set up and everything, so it was all taken care of anyway. And I had to stay for four days, so their staff treated me amazingly, made sure everything was taken care of with me and my wife, so.*

Excerpt - Document: 101 Respect Interview-cleaned.docx, Position: 3538-4139

*So it’s not just a matter of showing you, the patient, respect, but it’s also a matter of kind of showing your family respect as well. Is that right?*

*S2 07:35 Well, yeah. I mean, she stayed overnight every night with me, and they would get her water, and they would let her order food as well and stuff like that. So just showing them respect, getting her pillows, making sure she was comfortable when she was sleeping there and kind of going above what you would normally expect a hospital to just care of the patient. They were kind of taking care of her as well and making sure she was comfortable.*

Excerpt - Document: 101 Respect Interview-cleaned.docx, Position: 5720-6814

*has there ever been an encounter in the past with a previous doctor or someone else where you experienced a lack of respect?*

*S2 10:42 Yeah. I mean, I’ve had some emergency room doctors in the past where you go in and it feels like they’re just trying to kick you out. They don’t want to really deal with your issues of what’s really going on. So I have had that where it just seems like they got so much going on that they try to just quickly diagnose you. I mean, the first time I went in for the [issue related to my surgery], for example, they tried to diagnose it as just [being related to a preexisting condition], and they wouldn’t do a second ultrasound. They ended up sending me home with some Zantac. And then two days later, I had to go back to the emergency room, and they finally decided that, yeah, [I needed to have surgery]. Even though the first time I was in, the CAT scan showed [the affected area], and the doctor said that they knew that it needed to be taken out. But the surgeon said that they couldn’t see clearly enough on the ultrasound, so they weren’t going to do it.*

Excerpt - Document: 150 Respect Interview-cleaned.docx, Position: 403-1293

*So could you give me some examples of the sorts of things that make you feel respected in your medical care?*

*S2 03:04 Being listened to, and not feeling like they already are diagnosing me before hearing everything I have to say.*

*S1 03:13 Okay. All right. And so along those lines of communication, are there any other aspects of communication, whether it's with the doctor or the reception staff, the nurse, anything else where you might-- anything else that you can call out as being respectful?*

*S2 03:51 No, I think listening and not feeling-- well, I'm not sure how to say it. Not being lumped into a certain category without hearing everything. I don't know how to say it better, though.*

*S1 04:14 Okay. So let me know if I’ve kind of got this right. Is it maybe that the staff don't have any sort of preconceived notions or--?*

*S2 04:31 Yeah. Yeah. That's a good one. Yeah.*

Excerpt - Document: 150 Respect Interview-cleaned.docx, Position: 1660-2760

*All right. And so now I'd kind of like to flip the question on its head and ask if you could give me some examples of the sorts of things that might make you feel a lack of respect in medical care?*

*S2 05:32 I guess it would be the opposite, just no eye contact, meaning that I don't feel like they’re listening to me. And if I go in and I say, "I'm coming in because my stomach hurts," and it's just like, "Oh, yeah. The flu's going around," like, "No, I want you to hear everything I have and hear I have stomach pain plus maybe I have something else." And it doesn't seem like it's [inaudible] when they just jump to conclusions. What was it? When I go in and I-- I had to go in for fertility issues and it felt like, "Okay, this is the steps that you have to take." And it's like, "Yeah. I've already done this. I need you to listen to my personal story with it. I don't want you just to go through the motions that you have to based on everybody else. I want you to hear exactly what's going on with me." So I guess it's the exact opposite of feeling respected is how I feel I'm not respected.*

Excerpt - Document: 150 Respect Interview-cleaned.docx, Position: 3506-4191

*And then policy-wise, is there anything that you can see as having or showing a lack of respect?*

*S2 08:09 Yeah. There are some policies or guidelines that they have to do for everyone. And it felt just-- I guess I have to say an example, but I don't know how to say it. But when you get pregnant and you go into the doctor, like, "Okay, well we need to test you for all these STDs." And like, "Well, no. I'm not going to be-- why would I be tested for STDs? I'm married." And like, "Well, half of all pregnancies is not with the person they say." Like, "Well, that's not the case here." And it just felt like a very silly policy to have for someone who doesn't at all conform to that.*

Excerpt - Document: 118 Respect Interview-cleaned.docx, Position: 964-1425

*So just thinking a little bit more broadly, about the larger medical staff, say the nurses or even some of the reception staff, can you think of anything that they can do that would show respect?*

*S2 03:52 I mean, other than just communication. A smile is always appreciated. I mean, really with the nursing staff, I mean, a pleasant attitude, a smile I think go a long ways. Yeah, other than that I can't really think of anything that pops into my head that...*

Excerpt - Document: 118 Respect Interview-cleaned.docx, Position: 2145-2956

*could you give me some examples of the sorts of things that might make you feel a lack of respect from medical care?*

*S2 06:18 Are you referring to doctors in general or the whole clinical experience from when I get there to when I leave?*

*S1 06:29 All of it. Whatever [laughter] comes to mind for you.*

*S2 06:31 Okay. I feel that if I feel like I'm not being listened to if I feel that-- just providers, nurses, receptionists, check with me-- check you in, just having an attitude. They're not happy to be there or they've got something going on and it shows when you go to check-in. Just overall, not listening to concerns as being someone who's there because there's an issue. Feeling rushed a lot. The providers checking their watch or-- you just feel like you're being hurried out, is I think, big as well.*

Excerpt - Document: 118 Respect Interview-cleaned.docx, Position: 13777-14235

*And then was there anything more that you'd like to add about the way that you answered those questions?*

*S2 26:36 The only thing is it can depend on the day. So I've had really great doctors who have been really respectful, really there, on the ball, and then I've had others where I felt like I wasn't being heard or they didn't care. So I mean it just kind of-- that one's kind of a hard one, but I just went with my most recent [laughter] experience, so.*

Excerpt - Document: 154 Respect Interview-cleaned.docx, Position: 377-1283

*So could you give me some examples of the sorts of things that make you feel respected in your medical care?*

*S2 02:39 Well, being listened to and, yeah, eye contact. It's hard to describe. If someone's being dismissive, it's like a whole behavioral thing and not like-- it's hard to break down and pinpoint what it is that they're doing that's dismissive, but, I mean, yeah. Oh, I like it when doctors-- and I know that in-school doctors are told to use the proper terms for things, but it's frustrating when they don't want to break it down into layman's terms for you, especially if it's very clear that they don't know what's wrong. And instead of just coming out and saying, "I don't know," they're like, "Well, it could've been do, do, do, do, do, and it could've been do, do, do, do, do." Yeah, that stuff [laughter] and using big words. It's like, "Come on, come on. Just come clean with it." Sorry.*

Excerpt - Document: 154 Respect Interview-cleaned.docx, Position: 1390-1942

*And so you spoke a lot about what doctors can do, but if we think a little bit more broadly and think about the nurses, the intake staff, is there anything else that these individuals could do that could show respect?*

*S2 04:17 I'd say that, most of the time, I-- I mean, once in a while, you'll get a nurse that can feel like they went into nursing school just to be a control freak, but for the most part, most nursing staff, CNAs, medical assistants seem pretty nice, and I cut them some slack. I know their jobs are hard. They don't get paid enough.*

Excerpt - Document: 154 Respect Interview-cleaned.docx, Position: 2573-3194

*So I was curious if you could kind of give me an idea of the sorts of things that make you feel a lack of respect.*

*S2 05:38 I feel like I kind of just laid that out. It's kind of the same thing, I mean, regardless of who it's coming from, dismissive behavior, maybe not making eye contact. Oh, kind of passing the bucks and, "It's not our responsibility. It's somebody else's responsibility," that's frustrating. That's something I've dealt with before. Yeah. I mean, even if it's not your responsibility to do something or find the answer to something, it'd be nice to say, "Hey, let me get you somebody who can." Right?*

Excerpt - Document: 154 Respect Interview-cleaned.docx, Position: 3337-4997

*And have you ever felt a lack of respect in the medical setting based on who you are or how you look or what you believe?*

*S2 07:08 If it was, it never, to me, connected that that was the reason why. I've been reading more stuff, and more things are coming out, and I've read articles on stuff. I'm like, "Huh, huh." But, yeah, I never walked away thinking, "Oh, they were dismissive." Well, no, no, wait, I take that back. Yes, there have been interactions where it was like this idiot man doctor - excuse me - done some things that-- I know my body as a woman. There have been a couple of interactions like that, yes.*

*[silence]*

*S1 08:03 All right. And if you're okay with it, would you mind kind of talking to me about that experience?*

*S2 08:14 Okay. Hold on. Let me blow my nose.*

*S1 08:16 Oh, sure. Yeah, no problem.*

*S2 08:19 I've just gone into the office here. Years ago-- I mean, this is TMI too, but I didn't have any health insurance, so my mother called the advising nurse on her health insurance and asked if they would speak to me because I was bleeding anally quite a lot. I've had digestive issues off and on for years. But I spoke to a doctor on the phone, and he kept saying, "Are you sure you're not on your period? You're probably just on your period." And I'm like, "I know the difference between my vagina and my anus." I'm like in my mid-20s at this point. I think I know the difference. So, yeah, it still makes me mad when I think about it because I'm just like, "You're an idiot. Listen to what I'm saying."*

*S1 09:11 Yeah, no. So that's really one of those unfortunately terrible examples of doctors just not listening to patients. Yeah.*

Excerpt - Document: 154 Respect Interview-cleaned.docx, Position: 5007-6312

*Yeah. Oh, I had a-- it was actually a very creepy experience. So after college, I did an internship from summer away from home, and I went to DC. And I was not-- I guess I wasn't used to the air. What it came down to was like it was kind of the pollution, but the glands in my neck swelled up like golf balls, and it hurt just to swallow. And I was freaked out because I had never had that kind of thing before. And since I had health insurance through my internship, I just picked whatever doctor was easy to get to because I didn't know the area, and I was young and scared and in a new city. Right? So I went to this doctor, and he was a older man. He was, I think, probably like Indian or Pakistani. I mean, I don't know if saying that means anything, but here's what happened. I went in for glands on my neck, right? He looked at that, said, "Oh, it's probably the pollution in the air. It'll clear up." And then he's looking at me in like this creepy way and asked me-- what do they call that cervical exam? He asked me if I had had a Pap smear recently. It's super creepy. You go in for glands in your neck, and he wants to look at your vagina? I'm like, "Ew." Yeah.*

*S1 10:53 Yeah. Completely unrelated.*

*S2 10:55 Exactly. So, yeah, that was creepy. Yeah. So that's probably enough examples from me.*

Excerpt - Document: 172 Respect Interview - SP English translation - cleaned.docx, Position: 1388-1660

*in your medical care, is there a specific moment you can think of when you felt respected?*

*S2 03:23 In their communication when they’re going to check you out, explaining to you everything they’re doing or what they are going to do with you at the moment they check you.*

Excerpt - Document: 172 Respect Interview - SP English translation - cleaned.docx, Position: 1681-2053

*what can you tell me about other individual medical providers or the staff? How do or could the providers demonstrate respect?*

*S2 03:55 I’ve always communicated with my doctor; she has always been my personal doctor who checks me, the one from the clinic, and she does inspire a lot of trust in me for everything, including related to my results, my checkups, everything.*

Excerpt - Document: 132 Respect Interview-cleaned.docx, Position: 448-2617

*So could give me some examples of the sorts of things that make you feel respected in your medical care?*

*S2 02:59 I can tell you a couple of things that certainly made me not feel respected.*

*S1 03:05 Okay. Sure. We can start off with that.*

*S2 03:09 Well, it’s because, for me, it’s easier than trying to think of a specific example of something where I feel respected. It’s a lot easier for me to feel the things that were less [laughter]. Okay. So let me just think. I can reverse my thoughts here. Feeling respected, when somebody really hears what you’re saying and tries to help you find a solution to the problem makes me feel respected as opposed to being brushed off or getting the impression that they think that you’re a hypochondriac or it’s all in your head or something. There’s that. When I’m with the doctor I’m with right now, because the first time I went in to see her, she listened to what I had to say. I told her all the things I was struggling with. And her response was, “Well, you have all these things you want to work on. But let’s just take care of this one thing first. And everything else we’ll deal with as we come to it. Don’t try to figure everything all out at once.” And I thought that was very compassionate. Because most of the time, I would just get pat answers, “Well, if you would just do these things, life would be better.” And she was like, “Well, let’s take care of this particular issue.” And at that moment, it was a mental health thing. “And once we start getting that figured out, it’s going to be easier for you to address these other things.” And I’ve stayed with her. Because she really heard me instead of a pat answer that would make her like, “Oh, I’ve done my job now, next,” kind of thing, so being heard. I know with my kids’ doctor, she’s such a compassionate person. And she’s very thoughtful. And she’s really, truly concerned when they’re sick. And that makes me feel like she respects my children and us as a family rather than just a chart number.*

*S1 05:36 Okay. So--*

*S2 05:38 Oh, I think--*

*S1 05:40 Go ahead.*

*S2 05:41 --compassion and listening are the two things, I think, that make me feel most respected.*

Excerpt - Document: 110 Respect Interview_cleaned.docx, Position: 395-990

*So could you give me some examples of the sorts of things that make you feel respected in your medical care?*

*S2 02:31 When I bring up an issue that I'm having with my doctor and they actually listen and understand and can know where it's coming from versus just "Oh, there no history of it," or, "There's no reason why you should be having these symptoms," or whatever the case may be.*

*S1 03:04 Okay. So it's kind of having someone that really just listens and understands and has an appreciation for your concerns and the things that may cause you some anxiety [crosstalk]?*

*S2 03:26 Correct.*

Excerpt - Document: 110 Respect Interview_cleaned.docx, Position: 1018-1304

*And what is it about that listening and understanding that makes you feel respected?*

*S2 03:42 To know that I'm being heard and that the issues will be addressed and things will be done to come up with a reason why something is happening, or a good treatment plan for whatever comes up.*

Excerpt - Document: 110 Respect Interview_cleaned.docx, Position: 2098-2918

*can you give me some examples of the sorts of things that might make you feel a lack of respect from your medical care?*

*S2 05:49 So [laughter], excuse me, I'm sorry. I have a genetic condition that can present in many different ways in things that happen, and if [laughter] my doctor isn't very knowledgeable about the condition that I was born with, they kind of brush off my symptoms as "Well, it doesn't exist," or, "It doesn't matter." Because it's one of those things that anything could happen at any time. And it's like, "Well, you've never had that before, so why would that be a problem now?" So it's not really being heard from a point of view of "We know that you have this. We know that this can be associated with it," but they don't understand that and that's where it [laughter] can get very frustrating.*

Excerpt - Document: 110 Respect Interview_cleaned.docx, Position: 14088-14464

*And can you tell me more about why you answered these questions about respect in this way?*

*S2 27:15 I don't really feel a lot of respect from doctors based on why I'm coming in to see them. Most of the time they're really good about it, but sometimes you just don't feel like you're being listened to and they're hearing what you have to say and why you're there to see them.*

Excerpt - Document: 161 Respect Interview-cleaned.docx, Position: 2870-4349

*So thinking about some of the other individuals in the healthcare team, so the intake staff and the nurses, is there anything that they do that jumps out to you as particularly respectful?*

*S2 06:08 Yes. Well, just in the part I hear concerning, well, just myself or pretty much my mom, too, as a family member, you have the-- I don't know what else to say it, but it's really easy. They're really approachable and easy to talk to. And if there's any concern, they're there to help you out. And that includes doctors, nurses, and every medical member at the hospital, so that's a really awesome. We need to have it as well. And that comes from starting from the beginning of getting registered for the appointment all the way down the line to the end of your visit. They respect you. They know who you are, including nurses or the doctors or even the medical assistants. Especially if they see you there frequently, you get to see that difference. They're really happy, or you start a conversation with them, just really friendly, outgoing. They know you. That's really awesome to establish that relationship with them too. So it's not so much just with the doctors because you see them so frequently, but because the nurses also see you there often, and the medical assistants also are there, so it's really cool. I mean, it's really nice to see that you're going to get to see people that respect you, that care for you, and they want you to get well and take care of you. Yeah.*

Excerpt - Document: 161 Respect Interview-cleaned.docx, Position: 4481-6842

*And then thinking a little bit more broadly now, so the medical institution and the policies that it has in place, are there any policies that kind of jump out at you as being respectful?*

*S2 08:34 So the privacy, HIPAA, I know there's privacy. Yeah. That does help a lot with-- protects your information. That thing is not going to be shared with anyone else without your permission. Of course, I know that sometimes providers do need to share patients' medical information with other hospitals or providers, but it's within hospitals, providers. If not, they're sharing it with other people who are not authorized to do so. So HIPAA is a thing that the hospitals have. And yeah, that's really helpful to have. Outpatient consent forms, same thing. You are consenting to being seen and treated at the hospitals. But then again, it's there for information. It's specific information for them to share it, whether it be for the financial side, the medical side also. But then again, there is those policies in place for that in order to protect our information and then just share it with those that need to have that information. So yeah, that's really helpful to have. And then, of course, there's the other ones like the power of attorney forms the hospitals have where certain family members are authorized to have specific information for their patients. So yeah, I mean, all of that helps to have in our hospitals. Yeah.*

*S1 10:13 Okay, great. All right. So now with the--*

*S2 10:20 And also with--*

*S1 10:20 Oh, go ahead.*

*S2 10:21 Sorry. And I did remember something else too. Even employees that get taken care of-- employees that work in a hospital. And when they get sick, we also get noticed and respected because there is-- we get treated with respect as a patient, too, and respect our information. Even though they know that we're actual employees from the same hospital, we also get treated with respect the same way a patient would. So it's across the board, across the board, so that's really awesome too.*

*S1 11:03 Okay. Interesting. There's this kind of a-- oh, boy, how do I explain this? This idea of equality regardless of not just--*

*S2 11:18 Yeah. So it doesn't matter if you're a patient or an employee and even if you work there. At the hospital, you're treated with the same respect and courtesy across the board, and that's really awesome.*

Excerpt - Document: 136 Respect Interview-cleaned.docx, Position: 3226-3632

*Could you now give me some examples of the sorts of things that would make you feel maybe a lack of respect in your medical care?*

*S2 07:54 Yeah. Maybe getting my information wrong, like my name or my chart. Not getting my questions answered. Kind of getting brushed off. Wow, there's so many examples. Having an even longer wait time [laughter]. Or having rude doctors or nurses. Is that enough [laughter]?*

Excerpt - Document: 136 Respect Interview-cleaned.docx, Position: 4899-5184

*And then, what else? I said just getting rude nurses or doctors, which I've never gotten rude staff before, but I mean, if I did that would be pretty frustrating because I mean, they're supposed to be professionals and I would want to be treated kindly because who doesn't [laughter]?*

Excerpt - Document: 136 Respect Interview-cleaned.docx, Position: 5312-5618

*Have you ever felt a lack of respect in the medical setting based on who you are or what you look like or what you believe? Do you ever feel like that's happened to you?*

*S2 02:29 No. No, on the contrary. Everybody has always been very respectful and kind and answered all of my questions [inaudible] to me.*

Excerpt - Document: 153 Respect Interview - SP English translation - cleaned.docx, Position: 1887-2734

*Do you think you can tell me something about the providers? Something that they maybe do specifically, or maybe even the receptionist? Something that they do in particular or something that one person has done with specific action to make you feel respected?*

*S2 04:25 Yes. They always ask me before touching me or telling me something if I understand, if they can do it. My [family member] always accompanies me to the consultations and they don’t realize that he’s my [family member]. They always ask me who the person is at my side. Everyone is good, good; everyone has been very respectful and friendly towards me. I don’t have any bad experiences from anything that has happened to me, no. Even the translators, when the doctors are going to examine me, they turn their backs so as not to see where the doctors are touching me or examining me.*

Excerpt - Document: 153 Respect Interview - SP English translation - cleaned.docx, Position: 4248-4684

*Could you imagine something that would make you feel like you aren’t respected?*

*S2 07:24 For example, for them not to respect me, they would be rude to me, they might treat me badly for being a Hispanic woman, or for not speaking English. What else can I say? I don’t know. I think those would be the examples, but they haven’t happened to me. I wouldn’t know how to explain it well, but I think it would be something along those lines.*

Excerpt - Document: 153 Respect Interview - SP English translation - cleaned.docx, Position: 17721-18179

*Okay, and now, could you tell me a little more about why you responded to these questions about respect in this way?*

*S2 01:30 Yes. Well, because I’ve felt respected. I’ve felt that they take their time with people, at least in my case; that they’ve always treated me well. They speak to me in my language, which is Spanish, and for that I thank them very much. And, well, they’ve always based our interactions having good behavior and by speaking well to me.*

Excerpt - Document: 107 Respect Interview-cleaned.docx, Position: 538-2060

*Yeah. So, for example, some people think about the way that medical staff interacts with them. Other people talk about maybe the sorts of policies that a medical institution has. Do any of those-- can you think of any examples of things that would make you feel respected from either of those perspectives?*

*[silence]*

*S1 03:36 Another way to maybe think about is if there is-- if you can think of a specific example when you felt respect from your medical care.*

*S2 03:45 Okay.*

*[silence]*

*S2 04:11 When I've made a choice with my care and my doctor understands it, I guess.*

*S1 04:29 So having a choice about the sort of-- do you mean about what doctor you see or do you mean a choice about whether you do something that they recommend or not?*

*S2 04:44 Yeah, that one.*

*S1 04:45 The choice about what you do with your medical care?*

*S2 04:50 Yeah.*

*S1 04:50 Can you think of any examples of when you've been able to do that? Or maybe if you have a doctor who's really good at helping you or allowing you to have that choice?*

*[silence]*

*S2 05:35 Well, [there's one that?] I think-- I may have chosen not to continue taking a certain-- I don't remember what medication it was, but it was not helpful, so I decided that it wasn't working and told my doctor about that.*

*S1 06:03 And how did your doctor react when you said that it wasn't working?*

*[silence]*

*S2 06:43 Just said that we can stop it.*

*S1 06:45 So it sounds like your doctor actually listened to you and let you make that decision for yourself.*

*S2 06:55 Yes.*

Excerpt - Document: 107 Respect Interview-cleaned.docx, Position: 2071-2379

*Can you think of any examples of something that might make you feel a lack of respect from your medical care?*

*[silence]*

*S2 07:18 I guess [if] I'm not being listened to properly.*

*S1 07:29 And can you think of any specific examples of when you've felt a lack of respect?*

*[silence]*

*S2 08:13 Trying to think.*

Excerpt - Document: 107 Respect Interview-cleaned.docx, Position: 2390-3576

*And it's okay if you don't have a specific example, but sometimes people say they-- sometimes people a lack of respect based on-- if they're treated differently based on who they are or how they look, or what they believe. Have you had any experiences like that?*

*S2 08:39 Can you say that again?*

*S1 08:41 Yeah. Have you ever felt a lack of respect based on who you are or how you look, what you believe, and anything? What language you speak, what your culture is, anything like that?*

*S2 08:55 I don't think so.*

*S1 08:56 Okay. Could you imagine anything-- it sounds like you don't have any specific examples of feeling a lack of respect in medical care, which is good. I'm glad to hear that. Can you imagine anything that might make you feel a lack of respect?*

*[silence]*

*S2 09:33 I guess [inaudible] just assume stuff about me.*

*S1 09:37 Yeah, and what kind of-- oh, go ahead.*

*S2 09:41 I guess assuming if I speak another language or something, or-- basically just assume who I am and not really ask, I guess.*

*S1 10:14 Okay. Yeah, and that sounds like it goes back to what you were saying earlier about them not really listening to what decisions you're making.*

*S2 10:24 Okay.*

Excerpt - Document: 122 Respect Interview-cleaned.docx, Position: 412-1009

*So could you give me some examples of sorts of things that make you feel respected in your medical care?*

*S2 03:11 I guess a doctor not feeling like they’re rushing me to get through to get to the next patient. I like when they listen and kind of include me in I guess the decisions that are being made. I’m not just telling that you need to do this and this and this when they-- like one of the things that my doctor says, “Well, you know, how about if we try this? What do you think?” I guess having the patient participate in the care as well and not the doctors who’re making all the decisions.*

Excerpt - Document: 122 Respect Interview-cleaned.docx, Position: 1081-1594

*What about the other medical staff, say the nurses or even some of the reception staff. Is there anything that they--?*

*S2 04:14 Usually, oh gosh. A lot of times I don’t even use the receptionist to check in. I just go in and go to the machine and do it myself and check myself and-- but for the most part, I mean, most of the nurses I’ve had have been really nice and engaging and just kind of ask simple questions and make like conversation and do what they have to do and then move on with the doctors, so yeah.*

Excerpt - Document: 122 Respect Interview-cleaned.docx, Position: 2901-3024

*So I feel really fortunate with the care team that I have that everything always is addressed and I feel respected and, so.*

Excerpt - Document: 122 Respect Interview-cleaned.docx, Position: 3471-4229

*And can you imagine anything that might make say your family or friends feel a lack of respect when it comes to medical care?*

*S2 02:06 No. I mean, I guess the big thing is just being listened to. And I’m in healthcare myself and so I hear there is a patient recently to me that said when they were going to see new dentures [inaudible] dental and they were like, “I just want somebody to listen to me. I don’t feel like you need to be listening to me.” So I’ve heard that a couple of times that, “I just want to be listened to. I want to be heard.”*

*S1 02:38 So that’s a real key part of respect for you is being listened to and having someone actually take the time to understand what it is that your concerns are maybe or what’s going on.*

*S2 02:52 Yes, yes.*

Excerpt - Document: 122 Respect Interview-cleaned.docx, Position: 18682-19051

*And was there anything else that you’d like to add about how you answered those questions?*

*S2 27:17 Not particularly. I mean, I feel like-- I guess in medical care there’s been times obviously where-- that's why I gave it four is because there have been times when I haven’t felt like I guess I’m being heard or my questions are being answered or things like that, so.*

Excerpt - Document: 123 Respect Interview-cleaned.docx, Position: 365-1584

*And I’d like to ask you to give me some examples of the sorts of things that make you feel respected in your medical care?*

*S2 02:15 I think when people make eye contact and when the providers would actually sit down. I’m sorry. Just someone’s knocking on my office door.*

*[interruption/discussion omitted]*

*S2 02:50 So going back to the question, when I feel respected is when the provider actually sits down and looks at me and talks to me. They don’t act rushed or at least have some perception and that they’re really there to listen to kind of my chief complaints or concerns or worries. And then, I like them to use hearing and reflecting and they kind of like regurgitate what I said back to make sure what I’m saying is what they’re hearing. I like people with gifted sign manners so that they have compassionate thinking and doesn’t minimize concerns but still is very pragmatic. So I like them if they’re focused but at least validate, “I hear that you’re concerned or something,” is when I feel the most respected.*

*S1 03:47 Great. So it sounds like people who are really listening but also able to, you said, pragmatically sort of solve the problem that you’re there for. Is that right?*

*S2 04:02 Correct. Yeah.*

Excerpt - Document: 123 Respect Interview-cleaned.docx, Position: 2212-4099

*And I guess I should ask can you think of any particular examples when you’ve really felt like you’re being really well respected?*

*S2 04:53 Yeah. So I was not well very much. I’ve had chronic stomach problems for over 20 years and I’m overweight but I’m not significantly overweight. I’m not like morbidly overweight. And so throughout the years, I got a new complaint with the stomach illness and I’ve had all sorts of weird experiences where people have drawn mice and some mice are skinny and some are fat. Last time, I was compared to fat donut which was really a weird thing. But the positive experience is that I was going to have maybe a stomach repair surgery and I went to see the surgeon. And he actually listened to all of my complaints and said, “Oh, okay. That makes sense. You’re a little overweight but you’re not that overweight and your symptomology doesn’t mean what you’re saying and then if I do this surgery without really checking into it, it can cause you a lot of harm.” He explained all the procedure to me, what that could look like and then he said I’m going to do some extra testing because I want to make sure. And then, knowing the cause because of the test and he referred me to-- they actually found the stomach illness that I’ve probably have had over 20 years, and it was because he actually listened to me and it wasn’t just like default, “Oh, you have to shed your pounds. This is severe.” Or this is like, “Hey, this doesn’t kind of match up.” I’m like, “Yeah. That’s it. That’s what I’ve been saying.” And so just that-- so his name was Dr. [name]. His attention to detail and really like, “What?” where he was just like curious about it too. I mean, it’s been a little bit of life-changing for me.*

*S1 06:33 Yeah. So it sounds like it wasn’t just a matter of showing respect but it actually had a clinical impact on your care.*

*S2 06:42 Yeah. It did.*

Excerpt - Document: 123 Respect Interview-cleaned.docx, Position: 4119-8710

*you sort of alluded to this but can you give some examples of times when you felt a lack of respect in medical care?*

*S2 06:52 Yeah. So on my journey with stomach stuff, when I was going in-- I mean, I was throwing up in my sleep and so I went into the examination room. So we don’t know if they’re primary care and the woman I saw, I said, “I’m throwing up almost twice a week in sleeping mode.” And she’s like, “Well, you only have two episodes. That’s good.” I’m like, “I don’t think that’s true.” You know, I get it. I’m a [occupation], I’m not a doctor but that doesn’t make sense because you can die when you’re hearing that phrase, that’s disrespect. And so she was just like, “No. Two episodes is good.” And I asked to talk to a specialist and she said I didn’t need to see a specialist. So she really didn’t validate my feelings nor did she wish my medical desire which I felt was ridiculous. And then, I went back. A week later, once again I was throwing up in my sleep. I went in and saw-- I don’t know if he was a nurse practitioner or something, and he was just like, “What? Some people are thin donuts and some people are fat donuts.” And he actually compared me to like a glazed, sprinkled Mustang donut and was saying that my digestive system is like the center of that donut and if I were just as more compact donut that was more fit, my food would just go through me. But since I was more spread out like that fat sprinkled donut, then I was going to continue to have problems, and I was just more shattered. Then, I said, “You know, as I’ve gone through this journey and I extraordinarily lost a bunch of weight and I’m still working out.” And then I got shin splint. I tore my rotator cuff. I’ve had a series of cortisone shots and all of these things, and I’m kind of afraid to jump back. And your family is like, “Well, a cortisone shot is better than not being fat.” And I think it could probably be two ways.*

*S2 08:50 So again, he was just so quick to jump on the weight bandwagon. He wasn’t listening to my symptomology at all, and he was just like, “Well, you know.” And then, this was like minimize shame and it seemed ridiculous to me also like is that really the best solution our medical providers can give us. It’s like, “Well, you’re obviously in some pain. Just go get another cortisone shot” which is weird because there’s research about cortisone shots that you’re only supposed to have so many and he was just dismissive of it. But then I had a really bad example where I had had a bad pedicure but not a chronic issue. But I had a bad pedicure and I had gotten an ingrown toenail. So I went in and I was telling the provider. They’re like, “Oh, just soak it.” So I did and it didn’t resolve. And so it ended up being where I had to have it removed. And I don’t what he was like a physician’s assistant or something. He had a student come in and said, “Do you mind the assistance?” I’m like, “You know, I really kind of prefer, no offense to you.” Whilst she’s trying to learn and now what has happened is-- so I felt pressured. He said, “She’s a very nice old student.” So I’m like, “Okay. She can assist.” They didn’t know my foot. My foot ended up being-- I don’t know. Well, at least 12 shots in my toe, so the toe ended up being bruised for a week. They removed half of my toenail. And because students used like a little bottlebrush with the root. They killed the toenail root. My toenail now can only grow about a quarter inch out and then it dies because they’ve permanently damaged my root. So I don’t know. Well, it wasn’t respectful. I didn’t want the student. They probably shouldn’t ask if a student is right there because it puts the patient in a precarious spot. When I said no, he didn’t accept no, “Oh, common. She’s just trying to learn; I’ll be here.” And then, she failed with that and now I have a permanent like-- does it kill me? No.*

*S2 10:52 But I’ve had to have that toenail removed a couple of times since then. I never had ingrown toenails before. It wasn’t like I had a chronic problem where I needed that level of treatment. And now, it literally dies off and it turns like almost a bluish color. It’s unsightly that doesn’t kill me but had he just listened to me in the first place perhaps I wouldn’t have this and I actually asked to see a specialist for it. And my providers said I didn’t need to.*

*S1 11:25 Yikes. Well, I’m sorry to hear you have so many examples of-- that sounds rough so I’m sorry you had to go through that.*

*S2 11:35 Like if I’m old, over 45 years, this wouldn’t have been bad, but I’m [in my early 30s].*

Excerpt - Document: 123 Respect Interview-cleaned.docx, Position: 9072-9894

*I’m going to ask the same question as before, which is are there any policies that you could think of that might show a lack of respect from an institutional level?*

*S2 12:17 No. Well, I don’t know if they have the policy but I-- first of all, [healthcare institution] is not necessarily a teaching hospital. But I understand they provide the interns their practicums from a university. So I honor and respect that but I think that at a point, you should probably ask and I don’t know if they have policies about it. So maybe they should implement those where they ask the patients privately without the student being present so we’re not put in a precarious spot. And then, if there’s any hesitancy, you can explain that or you can just accept no and move on. I’m sure there’s plenty of patients that would gladly consent.*

Excerpt - Document: 115 Respect Interview-cleaned.docx, Position: 873-1258

*Is there anything that the other medical staff-- like the nurses or maybe the reception staff, is there anything that they do or have done in the past that makes you feel respected?*

*S2 04:26 I can say that something that I like, all the doctors and nurses, I feel respected. They’re concerned about how I feel. They give me a water if I need to drink. There is nothing really specific.*

Excerpt - Document: 115 Respect Interview-cleaned.docx, Position: 3390-3678

*So to kind of flip the question and ask you, have you ever felt a lack of respect in the medical setting?*

*S2 08:57 No. That’s a big no.*

*S1 09:02 Okay. Can you--?*

*S2 09:05 I really like the clinic where I’m going to. The staff is always nice, always smiling. That’s what I like about them.*

Excerpt - Document: 105 Respect Interview-cleaned.docx, Position: 395-809

*So could you give me some examples of the sort of things that make you feel respected in your medical care?*

*S2 03:04 Just the doctors that [ I feel are?] concerned or not about my health. Just like some will really ask you what's going on with you. Whether if you're mentally and physically okay. And just concerned or they're just trying to get you in and out of their office. It just really shows the difference.*

Excerpt - Document: 105 Respect Interview-cleaned.docx, Position: 825-1322

*And what about the other healthcare staff, like the nurses or maybe some of the reception staff?*

*S2 03:49 Reception staff, probably maybe they can be a little more quicker about making sure everyone's checked in or just more time management, rather than just being on the desktop computer or their personal cell phone or whatever. They should care more about the patients and getting them all checked in and making sure that they're on time or they're early. And making sure that everyone's happy.*

Excerpt - Document: 105 Respect Interview-cleaned.docx, Position: 2019-2829

*And could you give me some examples of the sort of things that might make you feel a lack of respect from medical care?*

*S2 06:18 I'm not sure really… about that question.*

*S1 06:29 Okay. So I know you said-- if we kind of just flipped some of the answers that you gave before, you said that doctors feeling concerned was something that made you feel respected. And I think I heard you say something to the effect of if a doctor's just trying to get you in and out of the exam room. Would that be a show of lack of respect?*

*S2 06:58 Yes. If they seem very concerned and they want to get you better, get you on any prescriptions or medications you may need. And making sure they want to have to see you back in their office for any more issues you may have. If they're concerned about caring for you is important.*

Excerpt - Document: 105 Respect Interview-cleaned.docx, Position: 3089-4279

*Can you imagine anything that, say, the nurses could do that might show a lack of respect?*

*S2 08:20 Let me think. I'm not sure. No, I can't think of anything right now.*

*S1 08:37 Okay. I know we talked about the reception staff earlier. Is there anything that you can think of? I believe you mentioned if they're maybe not paying attention to the patient as much or kind of distracted with their phones. Would that be a sign of lack of respect on their part?*

*S2 09:00 Yeah, definitely. Even if you're just-- have to speak with the receptionist for a couple of minutes, they should be also concerned and get you checked in on time or earlier, to be able to see your house specialist or your doctor, nurse, whoever you may need to see. It's just if they're lacking in respect or actually don't want to-- seem to care to get you checked in or they're just on their personal phone or they're just worried about other paperwork and not concerned about their patient that needs to get checked in. Then that can be an issue because then that's a time-- like I said, a time management thing. Because what if the patient has another appointment following right after that or something else going on.*

Excerpt - Document: 142 Respect Interview- cleaned.docx, Position: 462-1181

*So with that in mind, can you give me some examples of the sorts of things that make you feel respected in your medical care?*

*S2 03:02 Definitely having that one-on-one and having the feeling that they're listening and actually making that eye-contact with me. And I know, initially, when I first have a new physician or nurse practitioner, it's hard to have that trust right away. So I would say, just having that care and sympathy towards me and the time, not feeling like I'm being rushed during my assessment with them. Yeah. So asking any pertinent questions and making me feel like my information is not shared with other people, and that I can feel comfortable with them. So maybe that reassurance as well. Yeah.*

Excerpt - Document: 142 Respect Interview- cleaned.docx, Position: 1230-2067

*Can you tell me what it is-- one thing I want to kind of focus on is that you mentioned the one-on-one time, the listening, the eye-contact. And that all kind of reminds me of the same thing. Can you expand on those things? What is it about that, that makes you feel respected, being listened to?*

*S2 04:25 I guess, I just shows that they care and not making me feel like I'm just one of many appointments that they have to get through. But that they actually want to put time and effort into patient care and in my health. The body language says a lot too. If they are facing towards me and, like I said, making that eye contact, makes it seem like they're trying to be more understanding and relatable in that sense, in that whatever I'm going through is. Okay, maybe relaying information the way that I would be able to understand it.*

Excerpt - Document: 149 Respect Interview - SP English Translation - cleaned.docx, Position: 2892-3355

*So, could you give me some examples of the things that make you feel respected in your medical care?*

*S2 03:35 I, at the– I’ve gone to my appointment with my doctors. They’ve treated me well. Aha.*

*S1 03:48 And, in what way do they make you feel good or what do they do that makes you feel that they treat you well?*

*S2 03:57 They’re kind.*

*S1 03:59 Mm-hmm.*

*S2 04:02 They’re kind to you-- the appointments. Well, personally, they treat me well. I have no complaint.*

Excerpt - Document: 149 Respect Interview - SP English Translation - cleaned.docx, Position: 3371-3514

*And, was there a moment or can you think of a specific moment when you felt respected during your medical care?*

*S2 04:27 Always, all the time.*

Excerpt - Document: 149 Respect Interview - SP English Translation - cleaned.docx, Position: 3544-3736

*And, what can you tell me about the individual medical providers at the clinic? Do they always show respect or do they also--*

*S2 04:42 Yes, everyone, everyone respects me. They are very kind.*

Excerpt - Document: 149 Respect Interview - SP English Translation - cleaned.docx, Position: 6025-6494

*And, at any time have you felt that they didn’t respect you as in the tone of the doctor toward you or your beliefs? For example, have you felt that you weren’t shown respect due to your race, ethnicity, language, things like that?*

*S2 08:42 Well, right now, I don’t remember. No. I haven’t seen that.*

*S1 08:47 Okay.*

*S2 08:49 Nevertheless, [inaudible] (she has been) being kind to you when you go to an appointment. Even the secretaries are kind. No. I haven’t had--*

Excerpt - Document: 149 Respect Interview - SP English Translation - cleaned.docx, Position: 14106-16610

*People who participate in the study do not have to do anything, but researchers just want to have permission to have access to these records, and this is because they want to see if the medications have different effects which affect people's health.*

*S2 20:57 No, that's very bad. Without you knowing, to give medications, and you don’t know anything. Well that's it - it's very bad, because you don't even know what they’re doing to you. No, for example, [inaudible](my example), since I have [illness], the doctor prescribed me a medication. It didn't do anything for me at the beginning. Then, my chest ached, so then she changed the medication to another one and started telling me to take four tablets in the morning. So, I saw that [inaudible] once. I remember that I took it once like that, but no, those tablets, kind of like giving me a lot of medicine in one day. I mean, in the morning four tablets, and my chest felt really bad. So, I didn't say anything to the doctor, I just dropped it down to two, instead of taking four. I took two because four, like, like my chest hurts. I mean, I mean, why do they prescribe so much medicine in one day? That is, all at once. Do you know what I mean?*

*S1 22:30 Yes. Yes. Yes.*

*S2 22:31 When she knows. I mean, I’m taking [illness] pills, and then she prescribed me-- the medicine was increased by two more tablets and I felt bad. I mean, why? When I had to drop it down to two pills. Do you know what I mean? I mean, I say why? [Inaudible] (she) doesn’t know or don’t know, in that aspect, I don’t know why. Do you get me? I mean, that’s wrong. When talking with her, either she doesn’t know or will just hit or miss. But, so much medicine is not right. I mean, without knowing what reaction you’ll have. Do you know what I mean?*

*S1 23:24 Well, yes as you said--*

*S2 23:27 And, I mean, at the moment that I-- that is, [inaudible] (its possible that I) that they-- as you’re telling me that they research and all that without the person knowing. Do you know what I mean? I mean, she didn’t know if I were to feel bad with four tablets all at once, in one day. I mean, I told her, "My chest hurts when I take this medicine." And I took one in the morning and one in the afternoon. My chest felt bad. Then she changed the medication, but taking four tablets at once, that was the worst. I mean, no, I don't know what's up. That's why I’m telling you. Sometimes you take things without [inaudible](?). Then it will happen to you. Do you know what I mean?*

Excerpt - Document: 151 Respect Interview - SP English translation - cleaned.docx, Position: 1264-1489

*could you give me some examples of the things that make you feel respected in your medical care?*

*S2 03:22 I have good treatment, and I’m completely satisfied with the way they have treated me. They’ve given me a lot of help.*

Excerpt - Document: 151 Respect Interview - SP English translation - cleaned.docx, Position: 2942-3238

*Now, could you - if you have - give examples of the things that make you feel that you aren’t respected in your medical care or if you had a moment that you didn't feel respected?*

*S2 06:07 No, I’ve never felt a lack of respect. At the clinic, all the people have the correct way of treating you.*

Excerpt - Document: 151 Respect Interview - SP English translation - cleaned.docx, Position: 3261-3558

*How about, have you ever, by chance, felt that you were not respected within the medical environment, because of who you are? For example, due to your appearance, your beliefs, your race, your ethnicity, your language or education or things like that.*

*S2 06:38 No, very good attention is provided.*

Excerpt - Document: 129 Respect Interview-cleaned.docx, Position: 413-958

*So could you tell me or-- sorry, could give me some examples of the sort of things that make you feel respected in your medical care?*

*S2 02:47 Well, like the appointments that I had even when I was pregnant or before, I felt like the doctors were really listening to my concerns, and also trying to explain how things would go and all the things that could be helpful for me to know without overwhelming my brain with too much information.*

*S1 03:14 Okay. All right. So for you, it’s a lot about the communication aspect of it.*

*S2 03:22 Yes, yes.*

Excerpt - Document: 129 Respect Interview-cleaned.docx, Position: 980-1978

*And so I know you mentioned the doctors but is there anything when we think about the other staff in medical care so whether it’s the nurses or even the reception staff?*

*S2 03:42 Oh. I have to be honest, I’ve always had a good experience with nurses and any people checking you in and everybody else that I’ve talked to. I don’t really have any complaints about it.*

*S1 04:00 That’s good to hear. All right. And so you said that you’ve had good experiences so far. Could you give me some examples of how they’ve treated you with respect?*

*S2 04:16 For example, the one that I can remember is like when I was in labor, the nurses were very respectful to what I wanted and if I either wanted or not wanted people in the room and not really making any comments even during or after labor and delivery. It was very, very nice and they were really good.*

*S1 04:46 All right. So that kind of being - what’s the word that I’m looking for? - kind of receptive to your needs and your wants.*

*S2 04:56 Yes, yes.*

Excerpt - Document: 129 Respect Interview-cleaned.docx, Position: 2302-3652

*So with this one, can you give me some examples of the sorts of things that might make you feel a lack of respect with your medical care?*

*S2 05:36 Well, I do feel and maybe I’m-- I mean, it’s just the way I feel. And I’m sorry to just kind of bring back again my pregnancy and stuff but that’s been my biggest experience with the hospital and medical care in the state. So I would have to say that I kind of felt a little pressure to have an induced delivery which I was not really-- which I did not want. So I did feel like they were kind of pressuring me too much and at the moment, I was-- well, of course, it was the first time for me having a baby so I was not really thinking and I just felt I got too much pressure into telling me, “You have to do this and you cannot leave,” and that kind of thing. So that was the only thing that I was not very happy with anybody. I just wanted like a little bit of time and maybe like some more time to talk to doctors and that they will kind of sit and tell me more like consequences and all those things.*

*S1 06:59 Okay. So feeling a little bit of pressure and not having the opportunity to kind of start that line of dialogue or the communication that we spoke about earlier.*

*S2 07:10 Yes. I mean, before that I had like-- I don’t really have any complaints but just like for my delivery, I do have some.*

Excerpt - Document: 108 Respect Interview-cleaned.docx, Position: 353-998

*So could you give some examples of the sorts of things that make you feel respected in your medical care?*

*S2 02:22 I think when a doctor or a care provider knows-- so I’m actually a midwife and so I do have a level of medical knowledge as well and just sort of. Some care providers are really good at acknowledging that there are things that I do or don’t know about and others aren’t. So I think when you’re seen as an individual and asked like what you know about or what you don’t know about, what you need more information about or what you don’t need more information about and sort of just treating everybody the same. It feels respectful.*

Excerpt - Document: 108 Respect Interview-cleaned.docx, Position: 1883-2720

*Can you think of anything that may be a policy that a medical institution could have that might show respect?*

*S2 04:34 Yeah. I mean, I think when every person really introduces who they are and what they’re doing when I come into a room; that feels nice. And I think that in general, most of the care providers and most practices that I’ve interacted with which is quite a few are-- I mean, they’re not-- most of my interaction with practices are not my care providers but I’m there with another client who-- like a client of mine and we’re sharing like, you know, what I’m trying to say? I’m taking a client of mine or a patient of mine in and we’re seeing someone else together. But and so that’s when I had the most interaction with other care providers and hospitals and things. And I think that in general, they’re very respectful.*

Excerpt - Document: 108 Respect Interview-cleaned.docx, Position: 2741-3661

*I promised I would ask the opposite question which is if you have any examples of things that make you feel a lack of respect in medical care?*

*S2 06:01 Mm-hmm. And so the two things that came to my mind. One of them was when I was like 15 years ago or something but I had decided not to vaccinate my kids and the doctors were like really-- I mean, made me feel really bad and uncomfortable, and I never wanted to go back to a doctor again because they made me feel like I was making a bad decision for my children. And another time was actually more recently. I’ve had a history of [condition] for 20 years and the question that the care provider asks was like just super basics like as if I had never done any research or knew anything about my own body or why I possibly have [condition] when I’ve been like struggling with it for 20 years made me not want to talk to care providers about what’s going on with my body.*

Excerpt - Document: 108 Respect Interview-cleaned.docx, Position: 4700-5485

*I just asked you to expand a little bit on, I guess, how those examples of not feeling respected made you feel about the medical setting [crosstalk].*

*S2 08:52 Not the ones I interact with, yeah. So, yeah. When you go in and they like rather than knowing who you are and why you’ve made a decision, or what education you’ve gotten before you made a decision, or like really listening to where you’re coming from. It’s just like blanket information that they give to every person which really is-- because we don’t have the time to get to know you or remember you because they’re seeing so many people and just the whole system is like overworked. And it does often feel like, “How many people can we get in and out of here?” instead of like, “Are we truly caring for these individuals?”*

Excerpt - Document: 108 Respect Interview-cleaned.docx, Position: 5346-5485

*And it does often feel like, “How many people can we get in and out of here?” instead of like, “Are we truly caring for these individuals?”*

Excerpt - Document: 126 Respect Interview-cleaned.docx, Position: 396-1543

*So could you give me some examples of the sorts of things that make you feel respected in your medical care?*

*S2 03:08 I guess thinking back to just sort of like primary care visits that I’ve had, I think really specific things were like when the provider makes eye contact with me and really takes into account what I’m concerned about and addresses those things, then sort of checks in to make sure that my concerns have been resolved. Those are things that come to mind.*

*S1 03:36 Okay. All right. So it’s interesting. That eye contact part that you mentioned at the very beginning, what is it about that, specifically, that makes you feel respected?*

*S2 04:01 Oh, I guess it’s more that I’m thinking that I’ve had experiences with providers that were maybe just looking at a computer screen the entire time, and that sort of made me feel-- I don’t know if I’d say disrespected, but just sort of like they weren’t paying attention to me, yeah, which maybe is kind of disrespect. I don’t know. But yeah, yeah. I guess I’m just sort of mentioning that because I’m thinking of times when that was the case, and that didn’t feel so great as a patient.*

Excerpt - Document: 126 Respect Interview-cleaned.docx, Position: 1583-2287

*And so kind of beyond the doctors or providers themselves and thinking more largely to maybe the nursing staff and even some of the reception staff, can you think of any examples of how they show respect?*

*S2 05:06 I guess maybe a big thing is being respectful of my time. If I’m waiting a while-- I guess that’s not really something that they’re usually in control of though. That’s maybe a bigger administrative or a system-wide thing. But thinking back to, I guess, my interactions with medical assistants, I feel like a big part of what helps me feel respected is just how they interact with me. If they are smiling or just being really friendly and approachable, that’s a big part of it for me. Yeah.*

Excerpt - Document: 156 Respect Interview-cleaned.docx, Position: 418-915

*So could you give me some examples of the sorts of things that make you feel respected in your medical care?*

*S2 02:39 I think being listened to, in general, not being talked down to. I find that doctors who can appreciate somebody doing their own research on something, as long as it's not bad research, and accepting that information as something that could possibly actually be true and not just looking over it as a joke almost, I think those are the biggest things that make me feel respected.*

Excerpt - Document: 156 Respect Interview-cleaned.docx, Position: 999-1315

*So what is it about those things that makes you feel respected?*

*S2 03:35 I think just that-- I don't know. Maybe that I know myself and that they're aware-- that they're there to help, not just be the be-all and end-all of the answer, I guess. It's more of like a corroboration than just one person making decisions.*

Excerpt - Document: 156 Respect Interview-cleaned.docx, Position: 1331-1520

*Would it be fair to say that maybe the relationship should be a bit more kind of a back and forth or a two-way relationship rather than sort of just one way?*

*S2 04:27 Yeah, at least for me.*

Excerpt - Document: 156 Respect Interview-cleaned.docx, Position: 1623-2092

*but are there anyone else-- say, whether it's the intake nurses or the reception staff or just other people that are there in the clinic or the hospital, is there anything that those folks could do that would show respect?*

*S2 04:55 I don't know. No. I've had pretty good experiences with that kind of stuff. I think the same thing, just listening, maybe slowing down a little bit, not being in such a rush for intake nurses and everything, but I think that would be it.*

Excerpt - Document: 156 Respect Interview-cleaned.docx, Position: 2856-3619

*And so I want to ask you if you could give me some examples of the sorts of things that might make you feel a lack of respect in your medical care.*

*S2 07:04 I feel like with doctors, it's almost like they think they're better, the ones that I've hand anyways. There's been a few that just act like they know for sure when there's not really necessarily a certain answer for something, so I don't like that. And then not fully being aware of somebody's medical history--*

*S1 07:49 On the doctor's end?*

*S2 07:52 Yeah. That they don't double-check a chart to see if everything's the same before they actually talk to you or something. But I think the biggest one is just not talking to the patient like they're an actual person. It's almost like being talked down to.*

Excerpt - Document: 158 Respect Interview - SP English translation - cleaned.docx, Position: 1200-1515

*Could you give me some examples of the things that make you feel respected in your medical care?*

*S2 03:21 I like it a lot, and I go to my clinic because they respect my schedule, they respect my privacy. I’m at ease because they serve me well, they are respectful to me. I’m comfortable, due to many things, really.*

Excerpt - Document: 158 Respect Interview - SP English translation - cleaned.docx, Position: 2162-2487

*What can you tell me about the medical providers or a specific person, and how those specific people show you respect?*

*S2 04:48 They’re kind. If I ask them something, they answer me kindly. They are respectful. Up until now, they’ve never disrespected me, nobody, neither the doctor nor anyone. And, for me, that is kindness.*

Excerpt - Document: 158 Respect Interview - SP English translation - cleaned.docx, Position: 19673-20127

*Can you tell me a little more, if you can, about why you answered five to all these questions?*

*S2 30:01 Because where I go, to my clinics, they attend to me well, they treat me well, they’re friendly. Everything about the Charm Study was very good. I understood everything I had to do. As I say, I did it here at home. Then, when they called me to give me the results, they behaved very well toward me. There was a lot of respect and I understood things.*

Excerpt - Document: 128 Respect Interview - SP English translation - cleaned.docx, Position: 1073-1831

*So could you give me some examples of the things that make you feel respected by your medical care?*

*S2 03:56 The people, you told me? Yes, correct?*

*S1 03:59 Yes. It could be about the people, or maybe about the clinic you go to. Basically, what are the things that make you feel respected?*

*S2 04:15 By making time for me, by listening to my needs; although it almost never happens, I like to be heard when I describe what I have come in for or what I need them to tend to all in my own language. The behavior of the people when they are attending to someone is at times absent; they aren’t there, they aren’t present, though maybe in their bodies, yes, but they aren’t present enough to be able to listen and be able to understand what someone really wants.*

Excerpt - Document: 128 Respect Interview - SP English translation - cleaned.docx, Position: 4195-5425

*are there things that people or the clinic have done, or can you think of examples that make you feel disrespected?*

*S2 08:57 Yes. There have been many occasions, at least with my primary doctor, when I’ve felt really disappointed when-- sometimes I don’t go in for three years at a time because it’s so frustrating to meet with a doctor to whom I’ve come to with some discomfort or some concern and he tells me, “So what do you think is wrong?” If I’ve come for a consultation, it’s because I want him to investigate it, to figure out what’s wrong with me. And the fact that my illness might progress and they refuse to do studies or they don’t want to hear my needs, then that’s when I see that they aren’t valuing me as a person, nor my illness, my needs, and that’s when I feel really badly.*

*S1 10:00 Okay. It seems like you tell him that you have a problem but the doctor just says, “Don’t worry, I think it’s something minor,” or, “You don’t have to worry about it.” Sometimes it sounds like maybe in reality it could be something more severe but they don’t treat it with, I don’t know, the attention that you want to have.*

*S2 10:24 It’s not the attention that I expected or needed, and they are not providing me what I need.*

Excerpt - Document: 128 Respect Interview - SP English translation - cleaned.docx, Position: 5847-6707

*Could you imagine something that would make you feel not - apart from what you’ve already told me, of course - that makes you feel that they have no respect for you? Maybe, for example, stories or narratives of what has happened to your friends or family members who might have gone through something similar in which they didn’t feel respected.*

*S2 11:46 Well, normally, some doctors do this, and maybe not all, and sometimes in my community people comment, “Yes, they do it to me, too,” but we also have encountered really nice commentary, like, “My doctor really is a good person.” But just as soon as we hear of a positive comment, we also hear that other type of commentary, including some people with terminal illnesses where they’re telling them, “No, you aren’t sick with this, and you don’t have such and such type of benefits, so we can’t attend you.”*

Excerpt - Document: 174 Respect Interview - SP English translation - cleaned.docx, Position: 12832-13286

*Okay. It seems that you said five for everything, but, can you tell me a bit more about why you said five for everything?*

*S2 22:17 Because, so far, with everything that has happened, everything that they have asked me-- questions when they have spoken to me, from the beginning, from the exam, the providers, wherever I go, so far, they have always treated me well. They have always treated me with respect. They have always been very good people to me*

Excerpt - Document: 116 Respect Interview-cleaned.docx, Position: 1013-1320

*And if we were thinking specifically about the medical providers and the staff, whether that’s the doctors or nurses or maybe the intake staff or reception staff, can you think of any examples of how they can show respect?*

*S2 04:09 Just being courteous when I check in and-- that I’m not sure of [laughter].*

Excerpt - Document: 116 Respect Interview-cleaned.docx, Position: 2063-3190

*So now I want to kind of flip that larger question and ask you if you could give me some examples of the sorts of things that might make you feel a lack of respect from your medical care?*

*S2 05:56 I think that would be brushing off patient concerns, not really taking much time or thought into something they’re concerned about. I’m trying to think. I know one of my coworkers that I work with has had issues with her iron deficiency and such. And she’s tried to bring them up with her doctor and get some testing for it, when she found out one of the blood panels they did didn’t even search for the type of deficiency that she was curious about, but they just did that one and told her, no, she was fine, that sort of thing.*

*S1 06:41 I see. Okay. Huh.*

*S2 06:44 Yeah. Yeah, that sort of thing with the doctors and such not taking into consideration patient concerns. I’ve had other friends of mine that have had-- like they've been kind of dismissed of health concerns just because they’re overweight, like, ″You just need to lose weight and you’ll get better.″ Those comments were basically made. Yeah, stuff like that.*

Excerpt - Document: 116 Respect Interview-cleaned.docx, Position: 3307-4054

*Can you think of anything that might show a lack of respect, say from the staff side, like with the intake or reception staff?*

*S2 07:46 That is kind of hard to think of. I should know more of this since I’m a receptionist myself. I guess maybe just not-- sorry.*

*S1 08:11 No, no. Please take your time.*

*S2 08:16 Maybe not being very personable with them, just being kind of abrupt. Not really putting the extra effort in to help make sure they get everything settled and get what they want to get done and need to get scheduled and such.*

*S1 08:40 Okay. Great.*

*S2 08:47 Or making it seem like their job is more of a burden-- I guess, coming off like the patient's more of a burden than part of their job and someone they’re trying to help out.*

Excerpt - Document: 113 Respect Interview_cleaned.docx, Position: 398-1146

*So could you give me some examples of the sorts of things that make you feel respected in your medical care?*

*S2 02:46 Well, I recently had a stroke, so I've been going to see a doctor often. I like it when they're truthful and honest and when they don't beat around the bush. I feel more respected when people are just honest and look you straight in the eye and tell you what the conditions or what their concerns or what you need to do get better. Instead of going around the bush and saying, "Well, maybe you could try this," or, "Maybe if you try that." I'd rather just be frank and honest and I find that more respectful than beating around the bush. But I haven't had a bad experience. I do find [the healthcare system to be] very respectful.*

Excerpt - Document: 113 Respect Interview_cleaned.docx, Position: 1505-1849

*Can you think of any examples of things that they do that make you feel respected?*

*S2 04:13 Well, having someone paying attention to just you only and being friendly and honest. I find that respectful. I had a bad experience in the past where they're taking your vitals and having a fun conversation with their peers. I find that disrespectful.*

Excerpt - Document: 113 Respect Interview_cleaned.docx, Position: 3880-4822

*And then what about the doctors themselves or the medical staff? Sorry, medical providers.*

*S2 I'm going to be honest, I had an appointment recently and I don't want to name the provider. But I ended up in the hospital because I having some heart palpitations. And it was my ER follow-up and this was kind of recent, so the provider walked in and said, "Well, you're not dead." And I was like, "Really [laughter]?" Not lying. I was like, "Oh my God, that is so unprofessional." And then he's like, "Well, it looks at the hospital, they follow all the bullshit that they [inaudible]." I was like, "What?"*

*S1 07:56 Wow.*

*S2 07:58 Yeah. So I was just like, "Well, maybe he felt comfortable enough to speak that way," but I still thought it was-- I didn't feel offended but if it was someone else, I'd feel like that person would may feel disrespected or uncalled for.*

*S1 08:16 Okay. So it was sort of a lack of professionalism maybe?*

*S2 08:21 Yes.*

Excerpt - Document: 146 Respect Interview- cleaned.docx, Position: 426-4844

*So with that in mind, can you give me some examples of the sorts of things that make you feel respected in your medical care?*

*S2 03:05 Well, my personal experience is the ones that actually listen to what my concerns are, not what they think my concerns are, the ones that actually take in why I'm there. And then the ones that actually take that and - how do I say this? - try to help solve the issue instead of just plopping a Band-Aid on it and saying, "Oh, it's just this. You're fine," when then I have to go back and get a second or third opinion. And the second and third opinion don't match the first opinion, and they're calling the doctor idiot or whatever.*

*S1 03:52 Okay. So could you expand on that a little bit, and I want to just ask what it is about that that makes you feel respected.*

*S2 04:02 Well, for me, personally, I've battled some ovary cyst issues the last few years. We’re not sure if it's my IUD or not, but it's caused other symptoms. So my biggest concern was I wanted my IUD out. My first and second doctor, including my OB, were like, "No. That's not it. You're just gaining weight just because you're not active," and this and that. I went and saw a new doctor, same [healthcare institution] clinic, and a new OB, and they go, "Yeah. You're not as active as you were, but your IUD is clearly playing a role in your problem." Then they asked me-- the new two doctors asked me, "Okay. Well, what are your other symptoms? What else do you have going on?" But the second set of doctors actually had the time to go through my notes, my hospital notes, everything, and not notice that, "Hey, you've had a miscarriage in the last year. Hey, you showed up at urgent care bleeding profusely with a cyst ruptured. We need to get you in now and figure out what's going on." It was those second two doctors. They're the ones that said, "We need the ultrasound immediately. We need to do the Pap smear immediately. We need to do all these tests immediately."*

*S2 05:25 Well, when I called in to-- on the ultrasound schedule, because it was put in as an emergency ultrasound, they told me it was going to be three to six weeks for me to get seen. Okay, fine. Let's get it in. We got in, I think, four weeks after the initial rupture of my cyst that we thought was a miscarriage, but luckily, that one wasn't a miscarriage. That one was an actual cyst that ruptured. It just felt like I was going into labor. The doctor called me back the next day, the new doctor. My new OB called me back the next day and said, "This is unacceptable. I cannot believe they're making you wait four weeks to get seen. We need to have you go in now." Later that afternoon, I got a phone call from the ultrasound department because there's only two on the east side that I can go to. Because of [healthcare institution], we're limited to where we can go. She was able to get me in the next day. Because of her due diligence, because of her actually listening to me and taking the time to read over my notes, she understood that this wasn't just me having cramps, and the weight gain wasn't just over a period of time. This was a rapid weight gain.*

*S2 06:38 Everything else about me was healthy, but the rapid weight gain, the fatigue, all of my symptoms were like, "No. Something else is going on. I honestly think it is your IUD. We need to have that ultrasound to figure out what's going on." If it wasn't for her, I would've never had the ultrasound done. And then still to this day, two and a half years later, still battling the cysts. So, for me, it's one of those things like-- and, of course, that doctor ended leaving on maternity leave and never came back. So now I have a new doctor that's just like my first doctor, and it's like, "Oh, well, it's not a big deal. You are in your mid-30s. You're healthy. You're overweight, but, yeah, you're healthy." I'm like, "No. There's something going on. I know my body. There's clearly something going on. It shows on the ultrasound." But the new doctor is just as bad as the first one. The second doctor was fantastic, absolutely fantastic. I'm sad she's gone. She is the first one to actually truly listen to what my concerns were. She was talking like a friend, not like a professional.*

*S1 07:53 Okay. So that's really helpful. It sounds like listening to the concerns, but also, there being a follow-up with the urgency and action and it being timely as well. Does that sound like what--?*

*S2 08:05 Yes.*

Excerpt - Document: 146 Respect Interview- cleaned.docx, Position: 5111-6698

*Can you think of any policies that a medical institution has in place that makes you feel like they're respectful? What kind of policies show respect?*

*S2 08:41 I mean, I guess it shows respect with a policy that drives me nuts. Every time I go in for an appointment, whether it's my annual whatever or if I've seen three different doctors that day at the same clinic, every single one asks, "What's your last name? What's your date of birth? Why are you here?" blah, blah, blah. I guess they have to ask that, but when I'm in the same office for three hours seeing three different doctors, it drives me up the wall. I mean, it's great practice because you're verifying who you're talking to but literally have not left the room at all, and you send three different people in back to back. Yeah. I mean, it's good practice, but it's annoying as heck. I'll tell you that. It drives me up the wall. Like, "I know she handed you over my file immediately," but two seconds in between when the doctor left and the new one came in. I'm like, "Hmm, but you got to ask and verify everything again?" But I get it. You have to do it just in case. It takes that one person to mess things up and then lawsuit central. I get it, but it's definitely a bit annoying, especially when you have to go in every six months and see the same doctor, and then they ask, "Why are you here?" "Really? Look at my notes." They always ask you, "Oh, well, why do you need to be seen?" I tell them why. "Why are you asking why do I need to be seen?" But I get it. They have to. They're consistent. I'll give them that.*

Excerpt - Document: 146 Respect Interview- cleaned.docx, Position: 6873-8590

*So now I'd kind of just like to shift it to the other side and ask, can you give me any examples of the sorts of things that make you feel a lack of respect in your medical care? Anything in particular you can think of, whether it was a time you felt a lack of respect--*

*S2 10:53 Just not listening. When someone tells you-- and you've been their patient for like five, six years, and you tell them, "Hey, something's wrong. I've never had this happen before. This is new." And they're like, "Oh, it's just this." I pulled some muscles in my shoulder last weekend, and the pain was unbearable, so I finally took myself to urgent care. She didn't even so much as touch my shoulder. She had me move my arms up and down and said, "Okay. You have a pulled muscle and a partial dislocation. Here's some pain medicine. Have a good day." And I'm like, "You didn't even touch my shoulder. You didn't hear a word I said. You didn't ask me what my pain threshold level was. You didn't try to manipulate my shoulder to see which direction it could go. You didn't ask about the car accident," that it turns out this is a sideline of the car accident. She didn't do any of that. So I was like, "Why did I go to urgent care?" And all she did was prescribe me pain pills. I'm like, "I don't want pain pills." She didn't do X-rays. She didn't do ultrasound. She didn't do anything on the shoulder. So how can you tell me it's a partial dislocation when you didn't even touch it?*

*S1 12:06 Okay. So the feeling--*

*S2 12:06 So really, it comes down to--*

*S1 12:08 Not listening and then not taking your concerns seriously, it sounds like. Okay. And, yeah, are there examples that you can think of?*

*S2 12:18 No, not off the top of my head.*

Excerpt - Document: 146 Respect Interview- cleaned.docx, Position: 9306-9752

*I want to go back to what you said about at least your chiropractor. Did you feel like that was a lack of respect at all towards you?*

*S2 13:27 No, absolutely not. No. Because how they worded it, it wasn't just, "Oh, you're fat. You need to lose weight." It's, "Hey, what can we do to get you back to where you were pre-accident, including mind, body, everything?" How they went about it, how they worded it did not make me feel-- no, not at all.*

Excerpt - Document: 146 Respect Interview- cleaned.docx, Position: 9885-12330

*So in that case, I want to ask you, can you imagine anything that might make you feel a lack of respect? And if you can't think of anything of yourself, maybe for friends and family just to try to kind of get a sense of what things you might find disrespectful.*

*S2 14:16 Well, for me, it wasn't a lack of respect. I found it quite comical, but I'm a person you can't offend easy. When I gave birth to my son, when she was stitching me up-- because I tore on one side, but I didn't tear where you normally do. Well, I asked her how many more stitches needed to be done. She had already done three, and she said we might need to do about 10 or 12. But because the numbing was wearing off-- that's why I asked her. I could feel each stitch, so I was like, "How many more do we have to do? Because if we don't have to do too many more, let's just stop where we're at, and my body will heal." And she goes, "Well, if I do too much more, it will affect your sex life." My husband and the male nurse that were there-- my husband made a comment along the lines of, "Yeah. Let's do the virgin stitch. Let's keep it there. Keep going." And the nurse and my husband high fived over my stomach, and the male nurse immediately recoiled back, and my husband's just laughing. I'm laughing too. I'm doped up. But that's just humor. It was a joke.*

*S2 15:21 The doctor and the nurse, however, that were inside stitching me up were not happy about the comments, and I have never seen a woman glare at a man so hard as that doctor did to my husband. But I didn't find it disrespectful. Someone might, but I didn't. I thought it was hilarious. It was the timing. It was funny. It was super funny. The doctor did not think so and neither did the female nurse, but the male nurse-- I find it funny, but we have a warped sense of humor in our family. The male nurse that was there, I have to say, was fantastic. He was by far the most amazing nurse. Out of the five, six days we were in the hospital after having my son, he was the only one that was like, "Hey, do you need anything? Are you okay? What can I get you?" He was super, super helpful. So, for me, I'm like, "Whatever. You're dudes. You don't get it." But [inaudible], it might be offensive. For me, it was hilarious. Even the [inaudible] laughed about it. I mean, it's funny. So for some, that would be offensive, but in our [inaudible], it was perfect. It was good comedy to relieve a scary situation, so.*

Excerpt - Document: 146 Respect Interview- cleaned.docx, Position: 12474-14380

*What about individual medical providers or staff? Can you think of any ways that they might be disrespectful or show disrespect in their actions or the way that they would treat you? Again, I know you've said that you haven't experienced anything directly, but can you imagine anything they might do?*

*S2 17:07 Well, I have a friend. She always feels like-- when she goes in for a Pap smear-- she has a male doctor. They always ask you, "Do you want a male or female doctor?" Every time you go in for that exam, they always ask you. And she has the same coverage we do. She's seeing the same doctors we do. And she always feels like they look too hard, like they pay too much attention at certain areas of her body, but for me, I don't. I mean, they're supposed to be looking there. They're supposed to be touching. They're supposed to be manipulating certain things. Depending on the exam they're doing, they need to do that. They need to touch your breasts. They need to touch your vagina. They need to put fingers in holes. That's part of the [inaudible] process. But I can see some women finding some of those processes to be inappropriate. They don't feel like that's okay. "Yeah. You don't need to be down there that long. You don't need to be touching that exact spot." I mean, I've heard stories. I've heard stories, but--*

*S1 18:12 So you can imagine yourself being in that situation and feeling a lack of respect?*

*S2 18:18 No, because I know that that's what they're there to do. That's their job. They're checking for certain things.*

*S1 18:24 Okay. Is there any examples that you could give me of something that you feel like it might show a lack of respect?*

*S2 18:41 Maybe if they made a comment about how nice somebody's breasts were or something, I could see that being offensive. But I've never heard of that actually happening to anybody, but I could see that being offensive for some people.*

Excerpt - Document: 146 Respect Interview- cleaned.docx, Position: 14545-17957

*Can you think of any policies in a medical institution which show a lack of respect?*

*S2 19:16 This may sound like a [inaudible] on my part, but nowadays, if you ask somebody if they're male or female, that seems to get everybody in an uproar now because they don't identify with either, or their birth certificate says one, but they identify as the other, and then that causes problems. But I don't--*

*S1 19:40 So what about that makes you feel like it's a lack of respect, if you don’t mind expanding on that?*

*S2 19:47 For me, I don't care. Just whatever your birth certificate says, that's what you are. But for some that they're born male, and they feel that they're female, they view that as, "Oh, you called me by the wrong gender. That's not what I am. How dare you?" And then they get all uppity and pissy about it. It's like, "That's what's on your birth certificate. That's what's on your driver's license. If you haven't changed your driver's license over, that's nobody's fault but your own for not fixing it or changing it." But your birth certificate determines whether you're male or female when you're born, so it's one or the other. So there is no other gender because you're male or female, period. So, for me, I could see how the people in that community would be offended, but they get offended over everything else too. So everything's offensive nowadays to most people.*

*S1 20:41 Okay. Well, any other policies that you can think of in a medical institution that show a lack of respect?*

*S2 20:50 Well, I do think it's funny how-- I don't think it's offensive, but I just think it's funny how on the race thing when you're filling out paperwork, it's got all the different ones except it goes to white. It doesn't say Caucasian. It says white. So okay, like Island Pacificer, Hispanic, Latin, African American, all the different ones, but then it says white. So I think that's kind of comical. I could see that offending somebody too. But policy-wise--*

*S1 21:30 Sorry. I want to go back to that a little bit. What about that do you think you or other people could find offensive about it just to--?*

*S2 21:40 So we're in the state now where everything has to be politically correct. You can't say black. You have to say African American. You can't say Mexican. You have to say Hispanic. There's certain words you can't use. But when it comes to white people, then you can say white. White's okay, but you can't say black. You actually have to pronounce African American. Well, if they're not American, it can't be African American, so that doesn't work. But for white, they're slowly switching over to Caucasian because that's offending white people, which it doesn't offend me. I'm like, "Whatever. My skin is white. I'm white." But for my son, who is half Native American and half white, that's weird. White isn't a color when everything else is the definition, but white is white. But a few people get uppity about that. Like I said, I don't. I don't really care what color you are. I don't care about any of that, so.*

*S1 22:49 Okay. Thank you for sharing that. Any other examples that you can think of or things that you can imagine that would be offensive-- sorry, offensive. I just got that word stuck in my head. That would show a lack of respect in medical settings for you?*

*S2 23:10 No, I cannot. That would be it. Just the little things that people get upset about because of verbiage.*

Excerpt - Document: 146 Respect Interview- cleaned.docx, Position: 41751-43143

*And just any quick notes about why you answered those questions that way?*

*S2 52:20 Like I said, I had a great experience with your people. They were fantastic. I didn't feel like I was being-- I didn't feel like I was a test subject. I felt like I was-- like I said, a conversation with a girlfriend or a friend at the coffee shop. I didn't feel like I was being viewed under a microscope so to speak. Whereas the medical field, I trust my chiropractor and my massage therapist and my physical therapist more than I do my actual doctors. But my chiropractor and my physical therapist, I see more partially because of the auto accident. But I feel like I'm not a patient. But they don't say, "Hey, you're a patient here." They're like, "Oh, hey, Cindy's here today," or they come in and they know what's going on. It doesn't matter whether I've been in twice this week or I haven't been in in a year. They still know what's going on. They'll ask questions like, "Oh, hey, how did this show go?" It's like, "That was like six months ago. What are you talking about?" and I'll forget about it. It's like, "Oh, well, you went to this car show, and how did you guys place?" and blah, blah, blah. I mean, I've been going to the same clinic for almost [inaudible] years now, but I don't feel like I'm a patient there. Whereas at the doctors, I feel like a patient. I just want to get in and out, so.*

Excerpt - Document: 148 Respect Interview - SP English Translation-cleaned.docx, Position: 939-1477

*Could you give me some examples of the things that make you feel respected by your medical care?*

*S2 02:45 By medical care?*

*S1 02:46 Yes.*

*S2 02:48 The way that the assistants as well as the doctors treat me is always respectful.*

*S1 02:57 Okay. And do you have examples of things that the nurses or doctors at the clinic have done?*

*S2 03:14 For example, at any checkup, before anything else, they first ask me if it’s okay for them to perform whatever procedures may be necessary. They don’t do anything without asking for my opinion first.*

Excerpt - Document: 148 Respect Interview - SP English Translation-cleaned.docx, Position: 3352-4543

*have you ever felt that the people in the medical care environment haven’t respected you due to who you are? For example, because of your race, your ethnicity, your language, things like that. You’ve never felt like you’ve had--?*

*S2 06:34 Well, yes, on one occasion, because I don’t speak perfect English.*

*S1 06:40 Could you tell me a little more about that experience, or--?*

*S2 06:47 When I was sent to a research study and the person who assisted me spoke English, and I answered her as best I could, that’s when she could tell that I didn’t speak English perfectly, and then afterwards she didn’t even face towards me to ask me things. She just packed up her things, in this rude way.*

*S1 07:11 That’s a very interesting example because sometimes one hears examples like what you said, when one can’t speak Spanish or English, and sometimes people get frustrated and don’t feel respected. Now, could you imagine something that, apart from that, would make you feel that someone doesn’t respect you? Apart from the language or communication problems?*

*S2 07:44 No, just that situation, the one I told you about, when she started to carry on with things as though she were upset, this person.*

Excerpt - Document: 157 Respect Interview-cleaned.docx, Position: 382-993

*So could you give me some examples of the sorts of things that make you feel respected when it comes to your medical care?*

*S2 02:33 Let's see. Maybe just basic stuff to start with like when they ask you what you want to be called by and then they ask you your opinions or just maybe options regarding your healthcare or just different thoughts you have. Maybe they pay attention to you, and they give you enough time to ask all your questions, and they don't rush you, and they don't forget everything you say. They addressed every part of what you said. Yeah. That would be some things to start with, I guess.*

Excerpt - Document: 157 Respect Interview-cleaned.docx, Position: 1694-2072

*Is there anything else that you can think of maybe when it's in terms of interacting with the clinic staff or maybe with an intake nurse or anything like that?*

*S2 05:05 I don't know. My nurse at my primary care physician, she's just really nice and really helpful. So I guess if you had a good nurse who wasn't as nice, like maybe in a bad mood or something, that might affect--*

Excerpt - Document: 157 Respect Interview-cleaned.docx, Position: 2095-2489

*And then as far as the policies of the medical institution, can you think of any policies that show respect?*

*S2 05:48 A good question. Let me think about that for a second. It could be that-- I've had really good experience lately. I think, in the past, I've had some doctors who barely even looked at me or didn't think that what I was saying medically was true, so that was very discouraging.*

Excerpt - Document: 157 Respect Interview-cleaned.docx, Position: 2517-3514

*o you kind of touched on some aspects of the next question that I wanted to ask, but could you give me some examples of the sorts of things that would show a lack of respect? And I know that you just mentioned a lack of eye contact or people being in a bad mood. Was there anything else that you can think of?*

*S2 07:15 Let's see. Yeah. I mean, lately, I've just had good experiences, I think, but whenever someone's in a bad mood, you're definitely not sure if they're just having a bad day, or you just kind of feel like they're taking it out on you. You want to be in a good mood when you're discussing your health stuff even if they're having a bad day. And I think just following up to make sure you have any questions afterwards and giving you lots of chances to ask questions. Because sometimes things can be really busy, and you might forget to ask questions or something like that, and that kind of can be frustrating. Well, yeah, just feeling like you're not rushed is probably a big one.*

Excerpt - Document: 168 Respect Interview - SP English translation - cleaned.docx, Position: 1981-2264

*And what can you tell me about the providers? Do they do the same thing or do all the people in the clinic behave like this?*

*S2 03:51 Yes, with regards to the hospital, everyone is very, very, very kind, all the doctors, nurses, receptionists, they all do their best to be attentive.*

Excerpt - Document: 168 Respect Interview - SP English translation - cleaned.docx, Position: 2280-2747

*Now, can you tell me about the policies that a medical facility has? For example, what types of policies would show respect?*

*S2 04:23 Well, honestly, so far, in that sense, I’ve felt very respected, by everyone who’s attended to us. I really haven’t had any problems. I’ve felt good. I don’t see anything to improve at the moment.*

*S1 04:38 Okay. So, as you said, you can’t think of anything that you could change to feel more respected.*

*S2 04:49 No, I’m fine, yes.*

Excerpt - Document: 143 Respect Interview-cleaned.docx, Position: 1445-2516

*So maybe beyond thinking of just the doctors themselves, when you, say, interact with maybe the intake staff or the nurses, is there anything there that kind of jumps out at you?*

*S2 [04:23] If they hear one of my concerns and then ask a follow-up question, it feels like they’re really listening and trying to figure it out rather than just checking me in.*

*S1 [04:34] All right. Questions and concerns.*

*S2 [04:41] When they explain not just what it is that’s causing the problem but what could be done to [inaudible].*

*[call disconnected]*

*S1 [00:01] All right. I’ve restarted the recording. And sorry about that. So let’s--*

*S2 [00:04] All right. No worries.*

*S1 [00:07] All right. So the last thing I was able to hear was that when you had questions, the staff had some follow up-- or there were follow-up questions, excuse me, to your concerns that you raised.*

*S2 [00:21] Yes. Right. And then the next thing I was saying was that when I explained whatever my symptoms are, beyond just telling me what the problem is, when they explain what may have caused that.*

Excerpt - Document: 143 Respect Interview-cleaned.docx, Position: 2548-3338

*And then if you were to think of the medical institution, whether that’s the clinic itself or maybe the whole healthcare system, excuse me, can you think of any policies that might show respect?*

*S2 [01:08] Well, the general policy of not discussing your issue until verifying it’s the right patient is nice. Let’s see.*

*S1 [01:24] So that privacy and confidentiality aspect?*

*S2 [01:27] Yeah. Just double checking you’re talking to the right person before just diving in to any personal stuff. What else? I mean, I do appreciate the small talk that happens on the way to the-- when they’re bringing you to the doctor’s office from the waiting room. Like, “How was your day?” and all that. And those conversations, they can help to relieve the stress that you’re having [inaudible]. Yeah.*

Excerpt - Document: 143 Respect Interview-cleaned.docx, Position: 3522-4187

*But could you give me some examples of the sorts of things that might make you feel a lack of respect in medical care?*

*S2 [02:33] Like I said previously, when there’s a delay and there’s no communication about the delay, that’s frustrating because I know that they’re time is important, but mine might be, too. And let’s see. [inaudible]. I guess if they just come in, they call your name in the waiting room and then they just bring you to the doctor’s office and there’s no communication at all between that, it feels like they don’t want to be there. And I mean, I know it’s their job and they might not want to, but that makes me feel a little more stressed.*

Excerpt - Document: 143 Respect Interview-cleaned.docx, Position: 5790-6774

*So along those lines, have you ever felt a lack of respect in the medical setting based on who you are or how you look or what you believe?*

*S2 [05:44] One time, yes. It was not like-- it wasn’t religious beliefs but just beliefs in general. I had to see a specialist. And she decided she was a therapist also or something and asked me a whole bunch of questions about my mom and then kept telling me that my mom had messed me up. And it was weird because that’s not at all what I was there to see her about.*

*S1 [06:21] Interesting. All right. Okay.*

*S2 [06:30] Yeah. Some of the other nurses-- after she left the room, I said something to one of the nurses, and they were like, “Yeah. She does that to us, too.”*

*S1 [06:40] Oh, Jeez. Okay. So maybe this was an instance where it wasn’t just you as a patient, but it was this provider or physician themselves that perhaps didn’t know where the--?*

*S2 [06:57] Decided to talk about things that were not related to that [crosstalk].*

Excerpt - Document: 143 Respect Interview-cleaned.docx, Position: 7274-7673

*And can you imagine anything that might make your friends or your family feel a lack of respect in the medical setting?*

*S2 [08:00] Well, the one lady badmouthing my mother [laughter]. I let her know that that happened and that I didn’t like it and she didn’t like it much either. But I don’t generally have someone go with me when I see a doctor so they don’t interact with them at the same time.*

Excerpt - Document: 106 Respect Interview-cleaned.docx, Position: 418-1436

*So could you give me some examples of the sorts of things that make you feel respected in your medical care?*

*S2 02:35 Yeah, I think going through what I've been through in the last year, or two years now, is that I felt-- in the beginning that a lot of people who were dismissing my symptoms of trying to just brush them off like, "Oh, I might be depressed," or it might be this or that, and I had to make lots of appointments to keep coming back until I got a diagnosis that I had cancer. So that experience made me really realize how important it is for me to feel heard and acknowledged that what I'm experiencing is real to me even if there's no proof for the doctor by looking at blood work or films or something like that. That makes me feel respected when people spend time with you and treat you like a human being instead of just like a number or a case file. So that really was hard for me when making my appointments and coming to appointments. Just feeling like what I was complaining about had no value.*

Excerpt - Document: 106 Respect Interview-cleaned.docx, Position: 3528-4266

*So are there any other sort of policies that you can think of that might show a sign of respect other than the ones that you mentioned?*

*S2 07:12 Well, just admitting when you don't know the answer or you're wrong. That, I did mention to one of my doctors after seeing her because that was huge for me. It's just admit that you don't really know what's going on because to say that what you're experiencing isn't that and sorry, goodbye, wasn't enough for me. I just felt-- yeah, acknowledging when you don't have the answer but you're going to do your best to try and figure it out, I think is huge. Or just giving someone-- yeah, not this image that you know everything and that you can solve everything when really that's not the case.*

Excerpt - Document: 106 Respect Interview-cleaned.docx, Position: 6067-6543

*I think in healthcare too there's this idea - at least in this institution that I was at - that it's kind of "You're crazy" if you don't take the doctor's advice or agree with the doctor or provider that you're talking with. And that if you question things you're put into the "Oh, this one's a difficult patient," or "You have this high-maintenance patient now." So I think there's that stigma that also exists between people who do advocate for themselves and the providers.*

Excerpt - Document: 106 Respect Interview-cleaned.docx, Position: 13055-14024

*Is there any other ways that you think might be?*

*S2 22:09 If it's looking at medical records?*

*S1 22:12 Yes.*

*S2 22:12 Like you're asking specifically [inaudible]. Yeah, I guess to-- I feel like it's so important to also have a team-- when they're talking about people to not be judging people [laughter]. Because I used to [share?] an office space with these doctor's and I was just shocked by how much they would just talk negatively about the patients while they were reviewing stuff. And I was just like, "Oh, my God, why can't people just be more neutral and not impose their judgments these people who are people?" But when you're just reading through stuff you're like-- so, yeah, I think just having a team that's not making fun of people's medical records. I know it's something you can't control for necessarily, but just being really professional about it would be a plus. I know the patient never hears it, but [laughter] there's other people that hear it.*

Excerpt - Document: 131 Respect Interview-cleaned.docx, Position: 401-1392

*So could you give me some examples of the sorts of things that make you feel respected in your medical care?*

*S2 02:44 When I feel like the doctor has listened to my concerns thoroughly. I’ve had experiences with providers where I’ve felt like switching providers. Because I would go in with a complaint and it might be something random. And they’re like, “It’s probably because you need to lose weight.” That’s like, “Well, yeah, that’s one thing. But can you listen to what I’m actually talking [laughter] about?” So staying focused on what I’m there for and feeling like I’m being listened to is the biggest thing.*

*S1 03:20 Okay.*

*S2 03:22 And then just feeling like what I say is held within confidence, of course. So I would say those are the main ones.*

*S1 03:35 Okay. So yeah, the first one that you brought up was this issue of-- or not necessarily an issue. But this idea of communication, would you say that that’s kind of the most important element for you?*

*S2 03:58 I think so, yes.*

Excerpt - Document: 131 Respect Interview-cleaned.docx, Position: 1474-2136

*can you think about how the other individuals that you might interact with, say, the nurses or the reception staff, can you think of any ways that they might show respect?*

*S2 04:25 Well, I think good customer service skills, obviously, which I know is really hard after long days and things. But [laughter] good customer service is one. Again, communicating well, keeping me up-to-date if there’s a long wait for whatever reason, just letting me know, not just leaving me sitting in the waiting room forever [laughter]. Maybe just, I guess, feeling acknowledged, listened to, and that I’m not just another number in the line of the list of people to get through.*

Excerpt - Document: 131 Respect Interview-cleaned.docx, Position: 2163-3044

*And to kind of think about this in an even broader sense, if you think about the medication institution itself, whether that’s your healthcare system or this clinic, can you think of any policies that these institutions might have that can show respect?*

*S2 05:48 Well, I don’t know of any policies, specifically, if that’s what you’re asking. I’m not sure if I understand the question entirely.*

*S1 06:03 Or is there any that you could-- if there’s anything that you can think of that might be a good policy for maybe some clinics and healthcare systems to implement.*

*S2 06:15 Gotcha.*

*S1 06:16 Yeah.*

*S2 06:17 Yeah. Let’s see. Well, I think good policies would be returning phone calls in a timely fashion during business hours, making sure that confidentiality is a high priority. In general, having good training and support for their team so that everybody is happy in their jobs.*

Excerpt - Document: 131 Respect Interview-cleaned.docx, Position: 3072-5095

*So for this next question, I’d like to kind of flip the previous question on its head and ask you to give me some examples of the sorts of things that might make you feel a lack of respect in medical care.*

*S2 07:16 Okay.*

*S1 07:17 So I imagine that a lot of it could be just kind of the reverse of what we had talked about before with communication, and confidentiality, and privacy. But if there’s anything else that stands out to you or maybe any experiences that you’ve had in the past--*

*S2 07:36 Sure. Well, yeah, like the thing I mentioned before, where random self issues come up and the doctors blames weight management for the problem, rather than listening to the problem. I would say that that is something that not only has applied to me in the past, but people I know, where they don’t even want to go to the doctor. Because they don’t want to get lectured about their weight, or their diabetes, or whatever disability they have that they feel like the doctor blames every health problem on the one thing rather than looking at the person as a whole. But not everything is all about their diagnoses. And yeah, just the opposites of what we talked about a moment ago, like just feeling lot listened to, or dismissed, or lectured, rather than sharing information and feeling like you’re supported.*

*S1 08:45 Okay.*

*S2 08:49 And I’m also a person who likes to ask a lot of questions. So I appreciate it when a doctor is honest with me, like, “Well, we don’t know why it’s this way. But what we’re going to do to try to find out is this, this, and this.” Or explaining what the diagnosis means, rather than just saying, “Oh, it’s this thing. Here’s your prescription. Call me if it doesn’t get better.”*

*S1 09:13 Okay. And so I think those are interesting. Because there are also aspects of that communication element that we talked about with honesty being kind of an open line of communication. Again, you just mentioned being given information instead of handed a prescription and sent out the door.*

*S2 09:44 Right.*

Excerpt - Document: 131 Respect Interview-cleaned.docx, Position: 5122-6729

*Great. And can you think of anything from a policy side that might show a lack of respect?*

*S2 09:57 A policy side, so I don’t know if this true. But I have heard, in the past, from others that a lot of times, there’s a policy - and I don’t know if it’s the healthcare providers or if it’s required by the insurance companies either way - of asking about specific things such as weight management, like, “Did you mention their weight this time? Did you mention this this time,” certain things that they have to kind of check off the box. So I feel like sometimes that policy is kind of annoying. It’s like, “Well, no, I twisted my ankle. And I just need you to fix my ankle. I wasn’t here to talk about BMI this week.” So I think if there are policies like that, that you have to discuss every single item at every single appointment, it gets discouraging, I guess [laughter], to the patients.*

*S1 10:58 Okay. So what I’m hearing is sort of these mandatory questions, whether it’s the institution or the providers, aren’t so relevant to the issue at hand. Is that right?*

*S2 11:18 Right.*

*S1 11:19 Okay.*

*S2 11:20 Yes. And I understand. If they take your blood pressure and it skyrocketed, maybe you should have a discussion, of course. I broke my toe recently, for example, And I had to go urgent care. And my blood pressure was crazy high because I was in a lot of pain. They’re like, “Why is your blood pressure so high?” I’m like, “I know because my toe is bent the wrong way. And I’m in a lot of pain. Can you please help me fix my toe [laughter]? I will talk about blood pressure after I have calmed down.”*

Excerpt - Document: 117 Respect Interview-cleaned.docx, Position: 364-727

*Could you give me some examples of the sorts of things that might make you feel respected in your medical care?*

*S2 02:41 I’m really not sure because I’m only a private person but I think as long as they build sympathy or try to understand what the problem is and just being out fine with their patient. Yeah. I think I’ll gain more trust or easier to trust them.*

Excerpt - Document: 117 Respect Interview-cleaned.docx, Position: 900-1307

*Do you have any specific examples that you’d like to share of a time when you felt respected?*

*S2 03:48 When Dr. [name] told me about my brain tumor, what was some of the side effects, what might happen, just him being straight with me of all the complication that might occur during the surgery, I think, he really showed me a lot of respect there. Just being open, putting everything out in the open. Yeah.*

Excerpt - Document: 117 Respect Interview-cleaned.docx, Position: 1677-2653

*Can you think of any examples of the sorts of things that might make you feel a lack of respect from your medical care?*

*S2 05:20 Oh, yeah. There was one time that they put a lumbar joint in my back and I was sort of telling the nurse that I’m having some really sharp back pain. And they told me not to lay down because of my lumbar joint but I keep asking for some pain medicine. And [inaudible] for them to get it to me and I think they’re not understanding my pain. So I think that that was a lack of respect. Other than that, that’s it. That’s all I have.*

*S1 06:24 Okay. Yeah. Can you say more about specifically what it was that made you feel a lack of respect from that? Sounds like they didn’t understand that you were actually in pain.*

*S2 06:36 Yeah. They didn’t understand that I’m in pain or they just don’t want to listen. I’m not sure what it is but I was to the point that I had to lay flat which caused more problem. But I could understand the pain. That was it.*

Excerpt - Document: 117 Respect Interview-cleaned.docx, Position: 3104-4001

*So you gave a good example of somewhat of a way that-- it sounds like a specific person or a specific group of people didn’t really listen and didn’t really understand your pain. Can you think of any policies that the medical institution could have that might show that they didn’t really respect you?*

*S2 08:04 No, no. Because I totally understand what was going on. Because I know they’ve been giving pain medicine but it didn’t went down and I asked for more pain medicine, but they had to check in with-- I know they have to check-in with a doctor to prove it and because they can’t give me the same pain medicine. If that pain medicine is not working, they have to put me too on a different one or a more stronger one. My answer would be no because I know what’s going on. I mean, I know the process and I know it will take a little bit longer during the process. Yeah. My answer would be no.*

Excerpt - Document: 117 Respect Interview-cleaned.docx, Position: 16140-16939

*And I’m curious with medical care, for example, you said it was a five for how much you feel respected and a three for how much you trust it. I’m just curious what you see as a difference.*

*S2 29:31 Yeah. There was one incident, not incident which my surgical doctor could not do the surgery that day so they have a substitute or a different person perform or do my surgery. And my doctor told him how to go in and replace my shunt. But the guy wanted to go from a different spot which he went in, then it was kind of hard to replace my shunt the way he went in. Then so he went back to what my original doctor told him to do to replace my shunt. And the result of that him going in, the first time he went in have my left leg numb for three years. I mean, I trust medical care but after that-- yeah.*

Excerpt - Document: 145 Respect Interview-cleaned.docx, Position: 431-1115

*So could you give me some examples of the sorts of things that make you feel respected in your medical care?*

*S2 02:48 I think the number 1 thing that's important to me especially if you're going to see the doctors since I'm someone who doesn't seek out medical care very often is in just knowing that my provider is there and going to listen to all of my concerns. And then not feeling rushed during that time because a lot of the times I go, I know you have another appointment right after. But sometimes it can come off as dismissive. So I like having that at least demeanor that I'm being heard, and that all my questions are being taken seriously and answered thoroughly, I guess.*

Excerpt - Document: 145 Respect Interview-cleaned.docx, Position: 1217-1835

*is there anything in terms of your interactions with say the intake staff or the nurses or any of the other staff that you find respectful?*

*S2 03:50 I mean, just really good customer service. I mean, it is kind of customer service in a way. So just being greeted warmly, again, that just kind of active listening from all parties is really important. Patience in case I'm not understanding something correctly or thoroughly. I mean, depending on what's going on, if it's a nurse or maybe a phlebotomist or lab tech or whatever just being able to have clear communication about what's going on and why that's happening.*

Excerpt - Document: 145 Respect Interview-cleaned.docx, Position: 2110-3095

*So now, I want to kind of flip all those questions on their head and ask you if you could give me some examples of sorts of things that might make you feel a lack of respect from your medical care?*

*S2 05:15 Not following up in a timely manner if you're waiting for any type of results. I don't know if it's disrespectful, but I think it goes back to what I was saying before. Just making sure that someone gives you time and then again, that feeling where actually makes me feel like my issue is not necessarily important. And not having a professional demeanor. I mean, I know we all have bad days, but sometimes people get snappy and healthcare is a stressful job. Let's be honest. So I get it to some degree but not taking that out on your patients.*

*S1 06:09 Okay.*

*S2 06:19 Let's see. And I think if you don't get a full picture of what medical costs are going to be. So I guess not being thorough and transparent and sort of fees-- or again what's happening and why it's happening.*

Excerpt - Document: 145 Respect Interview-cleaned.docx, Position: 3169-3859

*And have you ever felt a lack of respect in the medical setting based on who you are or how you look or what you believe in?*

*S2 06:59 I think one time I saw an OB/GYN, and she asked me if I was planning on having kids. It was one of those kind of conversations. In terms of birth control. And I told her no. And her automatic response was why not? She was like, "I keep hearing this more and more these days." And it sounded more judgmental than like a curiosity of, "Well, why do you think that is?" It was just like, "Why are millennials not wanting children anymore?" Or something like that.*

*S1 07:31 Okay. So sort of a, as you said, judgmental response from a specialist.*

*S2 07:38 Yeah.*

Excerpt - Document: 160 Respect Interview-cleaned.docx, Position: 391-685

*So could you give me some examples of the sorts of things that make you feel respected in your medical care?*

*S2 02:52 I don't know. I don't really receive much medical care. I guess just listening to my questions, and - I don't know - I guess just paying attention to my questions and opinion.*

**Provider/staff support**: Provider/staff provide support by explaining procedures, answering questions, providing information, etc.

Excerpt - Document: 114 Respect Interview-cleaned.docx, Position: 326-1715

*So could you give me some examples of the kinds of things that make you feel respected in your clinical care?*

*S2 02:23 Often time, I find one of the biggest things is to be listened to. I’ve had situations in the past where my questions weren’t answered or they didn’t focus on the one thing that I was trying to focus on and focused on something else entirely that I didn’t really want to talk about at that time. So for instance such as I’m talking about I guess this is more of a [inaudible] because this wasn’t specifically happening to me. But talking about, I would like to-- my brain stopped functioning. I’m sorry.*

*S1 03:11 No take all your time you need.*

*S2 03:13 Basically, I sprained my ankle but then you go in there and someone will say, “Well, you need to lose weight.” That kind of thing. And though I understand it’s like-- I understand, “Yes, I’m overweight. Yes. I know I need to lose weight. I have talked about this before,” but people tell when it gets brought up and shoved on my face again when that’s not the reason that I’m even there. It comes off as extremely disrespectful and demoralizing, I guess. I think sticking to the topic, maybe perhaps trying to find what the actual cause of specific injury or bruising, where there is-- well, it should be done and not focusing on things that aren’t necessary at that point. And I’m trying to think what else might--*

Excerpt - Document: 114 Respect Interview-cleaned.docx, Position: 2003-2924

*Can you think of any experiences you’ve had where you felt, “I feel really respected in this moment in medical care?”*

*S2 05:08 Yeah. Actually with my primary care physician. She is extremely good at listening to me and asking the right questions and leading me to-- basically, I several times come in and say, “I think this may be the issue and she doesn’t discount what I say. She will think and say, “Okay. We’ll check it out if that may be the issue.” If she doesn’t believe it’s an issue then she’s like she’ll explain it very clearly as to why it shouldn’t be something I worry about. I feel not trying to talk above my head basically. She has learned basically where I am as a person, how I am able to communicate, and so I feel like she’s put in that effort to know me as a patient. And so because of that, I really enjoy being with her and I feel like she respects me as a person and not just as another patient.*

Excerpt - Document: 101 Respect Interview-cleaned.docx, Position: 394-1004

*So could you give me some examples of the sort of things that make you feel respected in your medical care?*

*S2 02:53 I guess just when they actually diagnose the problem right makes me feel respected. But it’s kind of one of those things where sometimes it feels like they’re guessing more than they’re actually diagnosing. Sometimes I may just feel-- when you feel like they’re guessing, you don’t really feel that much respect than them trying to take the time. But there are a lot of doctors out there-- my doctor now takes a lot of time and a lot of care in trying to figure out what’s really going on, so.*

Excerpt - Document: 101 Respect Interview-cleaned.docx, Position: 1119-2477

*What about the other medical staff? How could they show respect?*

*S2 03:50 I think nurses and them show quite a bit of respect, but just taking the time to actually talk to you and explain what they’re doing instead of just, “Sit and hold out your arm.” And actually tell you what they’re doing, if they’re taking your blood pressure or whatever, just explaining what’s going on and sharing. I’ve had nurses in the past where they don’t share what your blood pressure is or share what your heart rate is and actually take the time to tell you your whatever over whatever on your blood pressure and tell you if that’s decent, good, or something like that, so. Also, the nurses nowadays seem to be doing quite well with that, with letting you know what actually they’re doing and what they’re seeing and what they’re recording.*

*S1 04:54 Okay. So what I’m hearing is that you really appreciate kind of the ongoing dialogue and the back and forth of information and not so much them kind of just taking the information and not really filling you in.*

*S2 05:14 Right. And I mean, if they’re sharing with you, then they’re treating you like they respect you, that you’re capable of understanding what they’re talking about. When they used to keep it from you, it kind of felt like they were above you and that you wouldn’t understand what they were doing anyway, so.*

Excerpt - Document: 101 Respect Interview-cleaned.docx, Position: 2498-3444

*And then, so what about the policies that the medical institution has in place? What kind of policies do you think show respect?*

*S2 06:03 I mean, I think now-- I mean, recently, I had [surgery], and I didn’t have insurance. So with them being able to actually still go in and do what they needed to do and not treat me, yeah, like a leper, pretty much, because I didn’t have insurance and still do everything that they would’ve normally done, that was pretty great. I was pretty freaked out when I had to go in and didn’t know what was going to happen since I didn’t have insurance at the time. So that policy of being able to still take care of the patient and take care of what they needed to do. And then I had people come in and help me get Medicaid set up and everything, so it was all taken care of anyway. And I had to stay for four days, so their staff treated me amazingly, made sure everything was taken care of with me and my wife, so.*

Excerpt - Document: 101 Respect Interview-cleaned.docx, Position: 3538-4139

*So it’s not just a matter of showing you, the patient, respect, but it’s also a matter of kind of showing your family respect as well. Is that right?*

*S2 07:35 Well, yeah. I mean, she stayed overnight every night with me, and they would get her water, and they would let her order food as well and stuff like that. So just showing them respect, getting her pillows, making sure she was comfortable when she was sleeping there and kind of going above what you would normally expect a hospital to just care of the patient. They were kind of taking care of her as well and making sure she was comfortable.*

Excerpt - Document: 150 Respect Interview-cleaned.docx, Position: 1660-2760

*All right. And so now I'd kind of like to flip the question on its head and ask if you could give me some examples of the sorts of things that might make you feel a lack of respect in medical care?*

*S2 05:32 I guess it would be the opposite, just no eye contact, meaning that I don't feel like they’re listening to me. And if I go in and I say, "I'm coming in because my stomach hurts," and it's just like, "Oh, yeah. The flu's going around," like, "No, I want you to hear everything I have and hear I have stomach pain plus maybe I have something else." And it doesn't seem like it's [inaudible] when they just jump to conclusions. What was it? When I go in and I-- I had to go in for fertility issues and it felt like, "Okay, this is the steps that you have to take." And it's like, "Yeah. I've already done this. I need you to listen to my personal story with it. I don't want you just to go through the motions that you have to based on everybody else. I want you to hear exactly what's going on with me." So I guess it's the exact opposite of feeling respected is how I feel I'm not respected.*

Excerpt - Document: 118 Respect Interview-cleaned.docx, Position: 387-936

*So could you give me some examples of the sorts of things that make you feel respected in your medical care?*

*S2 02:40 I think number one, being the provider being on time or at least communicating if there's a delay with the appointment. I think that that's huge. I think that providers taking the time to acknowledge that-- especially, if you've been through something kind of scary or traumatic, taking the time to acknowledge that and answer your questions maybe more in-depth then what you might even be aware that you have deeper questions on.*

Excerpt - Document: 118 Respect Interview-cleaned.docx, Position: 2145-2956

*could you give me some examples of the sorts of things that might make you feel a lack of respect from medical care?*

*S2 06:18 Are you referring to doctors in general or the whole clinical experience from when I get there to when I leave?*

*S1 06:29 All of it. Whatever [laughter] comes to mind for you.*

*S2 06:31 Okay. I feel that if I feel like I'm not being listened to if I feel that-- just providers, nurses, receptionists, check with me-- check you in, just having an attitude. They're not happy to be there or they've got something going on and it shows when you go to check-in. Just overall, not listening to concerns as being someone who's there because there's an issue. Feeling rushed a lot. The providers checking their watch or-- you just feel like you're being hurried out, is I think, big as well.*

Excerpt - Document: 132 Respect Interview-cleaned.docx, Position: 448-2617

*So could give me some examples of the sorts of things that make you feel respected in your medical care?*

*S2 02:59 I can tell you a couple of things that certainly made me not feel respected.*

*S1 03:05 Okay. Sure. We can start off with that.*

*S2 03:09 Well, it’s because, for me, it’s easier than trying to think of a specific example of something where I feel respected. It’s a lot easier for me to feel the things that were less [laughter]. Okay. So let me just think. I can reverse my thoughts here. Feeling respected, when somebody really hears what you’re saying and tries to help you find a solution to the problem makes me feel respected as opposed to being brushed off or getting the impression that they think that you’re a hypochondriac or it’s all in your head or something. There’s that. When I’m with the doctor I’m with right now, because the first time I went in to see her, she listened to what I had to say. I told her all the things I was struggling with. And her response was, “Well, you have all these things you want to work on. But let’s just take care of this one thing first. And everything else we’ll deal with as we come to it. Don’t try to figure everything all out at once.” And I thought that was very compassionate. Because most of the time, I would just get pat answers, “Well, if you would just do these things, life would be better.” And she was like, “Well, let’s take care of this particular issue.” And at that moment, it was a mental health thing. “And once we start getting that figured out, it’s going to be easier for you to address these other things.” And I’ve stayed with her. Because she really heard me instead of a pat answer that would make her like, “Oh, I’ve done my job now, next,” kind of thing, so being heard. I know with my kids’ doctor, she’s such a compassionate person. And she’s very thoughtful. And she’s really, truly concerned when they’re sick. And that makes me feel like she respects my children and us as a family rather than just a chart number.*

*S1 05:36 Okay. So--*

*S2 05:38 Oh, I think--*

*S1 05:40 Go ahead.*

*S2 05:41 --compassion and listening are the two things, I think, that make me feel most respected.*

Excerpt - Document: 161 Respect Interview-cleaned.docx, Position: 383-1576

*could you give me some examples of the sorts of things that make you feel respected in your medical care?*

*S2 02:52 Oh, yes, exactly. Yeah. Especially during my doctors' visits, having our providers or pretty much my doctor know exactly what I have instead of having myself repeat for the second time or third time what I have and my condition. Instead of repeating or telling the doctor, "This is what I have," having them know exactly what I have and not repeating the information myself. That's awesome. I really like that with my providers, and my providers do know me. They know exactly what I have, and I don't have to repeat the information. So it's really helpful that way. You save time, and we go straight to what needs to be done. I really like that from my providers knowing that they know what I have. And even though we met for so long, they still know what I'm going through, what I need, and so that's really awesome. And especially, it does help when you see the same provider. That really helps a lot too. When you get to see the same provider, you get to establish a patient-provider relationship, and that's really awesome. It's also really helpful, and it helps your care.*

Excerpt - Document: 161 Respect Interview-cleaned.docx, Position: 1610-2723

*So interesting, one of the things that kind of jumped out to me about the things that you just mentioned was this idea of providers coming into an appointment or a meeting prepared and ready to kind of just dive right in.*

*S2 04:46 Exactly.*

*S1 04:47 So that's awesome. All right.*

*S2 04:49 Yeah, exactly. Yeah. And it really does help when you get to see the same doctor that's taking care of you because that does help with your care move smoothly and forward. And I do understand that sometimes it's not possible. I totally get it when-- and it has not been when I get to see other providers because they might not have appointments available, or they're out, or you get to see someone else because it's emergent or urgent, and you need to see someone else because the actual provider that you normally see is not available. And that's totally fine. I mean, I totally get it, and that has happened. But, yeah, it's really awesome when you get to see the same provider that you're assigned to, and they're the ones that know everything pretty much, and they're seeing it through. And it just makes it a lot easier.*

Excerpt - Document: 136 Respect Interview-cleaned.docx, Position: 419-869

*can you give me some examples of the sorts of things that make you feel respected in your medical care?*

*S2 03:21 Let's see. Being waited on time. That's a pretty big one. If I'm not feeling well I'd like to be seen as soon as possible. And being explained everything, especially me. I like to look up a lot of things to get more information and it's really helpful when the doctor explains more in-depth. And I think those two are the primary, yeah.*

Excerpt - Document: 136 Respect Interview-cleaned.docx, Position: 1130-1542

*So maybe thinking about individual medical providers or staff, maybe specifically your nurses or specifically your doctor's, can you think of anything that they might do that can make you feel respected? So you mentioned your doctor explaining things to you, can you think of any other examples maybe?*

*S2 04:51 Let's see. Maybe printing out the details-- well, they do do that. Something that they don't do or--?*

Excerpt - Document: 136 Respect Interview-cleaned.docx, Position: 2098-3167

*So what about any policies that a medical institution might have in place? Can you think of any policies that would show respect by chance?*

*S2 06:06 Oh, yes. Recently my daughter had gotten chicken pox and I wanted to make sure it was chicken pox, and I wanted to be seen by her pediatrician. And at first, one of the nurses had told me that it was not impossible to go to, to get seen, but since it's contagious that she recommended me not going in. And I had asked her if there was any way that we could still be seen? Maybe if there was a-- if there was a contaminating room that she can go into and she had to go and ask a doctor to see if that's something that could be done and eventually it was, but I had to ask her. Because at first she kind of said no. So that was, I guess, a little disrespectful.*

*S1 07:18 Okay.*

*S2 07:18 I mean, there should be a policy for that.*

*S1 07:22 Okay. So it sounds like there was a policy may be for it, but it wasn't communicated to you, is that right?*

*S2 07:32 It wasn't really communicated to the nurse because she had to ask.*

Excerpt - Document: 136 Respect Interview-cleaned.docx, Position: 3226-3632

*Could you now give me some examples of the sorts of things that would make you feel maybe a lack of respect in your medical care?*

*S2 07:54 Yeah. Maybe getting my information wrong, like my name or my chart. Not getting my questions answered. Kind of getting brushed off. Wow, there's so many examples. Having an even longer wait time [laughter]. Or having rude doctors or nurses. Is that enough [laughter]?*

Excerpt - Document: 136 Respect Interview-cleaned.docx, Position: 4455-4873

*Or what if they give me the wrong medication or something that I'm allergic to, or my child is allergic to, that would be a really big issue. And I would be furious [laughter]. Or just being brushed off or not getting my questions answered. The would be pretty frustrating because I mean that's the main reason that I go in to get answers and to get better, and if nobody tells me anything well, then how can I, right?*

Excerpt - Document: 136 Respect Interview-cleaned.docx, Position: 5312-5618

*Have you ever felt a lack of respect in the medical setting based on who you are or what you look like or what you believe? Do you ever feel like that's happened to you?*

*S2 02:29 No. No, on the contrary. Everybody has always been very respectful and kind and answered all of my questions [inaudible] to me.*

Excerpt - Document: 136 Respect Interview-cleaned.docx, Position: 6636-7747

*What about policies? We talk a little bit about policies in a positive sense. Are there any policies that a medical institution might have in place that you think shows a lack of respect? Sorry.*

*S2 04:32 I mean, we had talked about it a little bit already when I had taken my daughter for the chicken pox. I think maybe that policy needs to be stated-- or to be more clear with the staff because I was getting upset when the nurse had told me not to come in so as to not contaminate other babies and the elderly who are more prone to getting the same infections. But yeah, if I hadn't asked about maybe being secluded then she would've-- I mean, it wouldn't have happened. My daughter wouldn't have been seen. So yeah, maybe just make that policy more clear. I mean, if it is a policy because you weren't sure either, right?*

*S1 05:33 Yeah. So just communicating those policies may be, so everyone is aware?*

*S2 05:41 Yeah. Or maybe having a specific contamination room because-- I mean, everybody was just wearing extra gowns and face masks, but there wasn't any specific room that she was able to be taken into.*

Excerpt - Document: 153 Respect Interview - SP English translation - cleaned.docx, Position: 1370-1820

*Could you give me some examples of the things that make you feel respected in your medical care?*

*S2 03:27 For them to respect the individuality of the patient. I like that. For them to, since I don’t speak English, bring me a translator. Almost always in [clinic], it’s almost always in person, never by phone call, and that kind of makes me feel more sure about what I’m understanding and of what I’m trying to say. That has been really good for me.*

Excerpt - Document: 153 Respect Interview - SP English translation - cleaned.docx, Position: 1887-2734

*Do you think you can tell me something about the providers? Something that they maybe do specifically, or maybe even the receptionist? Something that they do in particular or something that one person has done with specific action to make you feel respected?*

*S2 04:25 Yes. They always ask me before touching me or telling me something if I understand, if they can do it. My [family member] always accompanies me to the consultations and they don’t realize that he’s my [family member]. They always ask me who the person is at my side. Everyone is good, good; everyone has been very respectful and friendly towards me. I don’t have any bad experiences from anything that has happened to me, no. Even the translators, when the doctors are going to examine me, they turn their backs so as not to see where the doctors are touching me or examining me.*

Excerpt - Document: 153 Respect Interview - SP English translation - cleaned.docx, Position: 18587-19306

*Once again, could you tell me a little more about why you answered these questions about trust in this way? I noticed that we’ve had a bit more trust in general compared to respect.*

*S2 03:03 Oh, my answer is because when I, I detected something wrong, I went to the doctor and they told me that there was nothing wrong with me; I went back to the doctor and they ran some tests on me, they did a biopsy, and they told me that it wasn’t [illness]. So then I was doing really well, until later when they operated on me and it turns out that I do have [illness], or in other words, then they said I did. So they don’t know, either; they’re really not sure when they’re running a test on you what’s really happening to you.*

Excerpt - Document: 122 Respect Interview-cleaned.docx, Position: 2901-3024

*So I feel really fortunate with the care team that I have that everything always is addressed and I feel respected and, so.*

Excerpt - Document: 122 Respect Interview-cleaned.docx, Position: 18682-19051

*And was there anything else that you’d like to add about how you answered those questions?*

*S2 27:17 Not particularly. I mean, I feel like-- I guess in medical care there’s been times obviously where-- that's why I gave it four is because there have been times when I haven’t felt like I guess I’m being heard or my questions are being answered or things like that, so.*

Excerpt - Document: 115 Respect Interview-cleaned.docx, Position: 430-824

*So could you give me some examples of the sorts of things that make you feel respected in your medical care?*

*S2 03:20 I’m going to look to my last appointment, which was yesterday. And I like some of the doctors. They try to help me with my problems. But the only thing that I don’t like is that all the options they give me, it doesn’t really help. Besides that, I’m not sure what else to say.*

Excerpt - Document: 115 Respect Interview-cleaned.docx, Position: 2764-3287

*So when you talk to the doctors about what it is that you need or any symptoms or maybe an illness that you’ve come down with, have they generally been pretty helpful?*

*S2 07:51 Well, yes and no. The only thing that I’m not really liking it-- because when I need some painkillers for my pain, because I have really, really serious back pain every month, they can’t really give me anything besides ibuprofen. Besides that, everything’s good. But I understand why, but at the same time, there’s nothing I can do about my pain.*

Excerpt - Document: 142 Respect Interview- cleaned.docx, Position: 462-1181

*So with that in mind, can you give me some examples of the sorts of things that make you feel respected in your medical care?*

*S2 03:02 Definitely having that one-on-one and having the feeling that they're listening and actually making that eye-contact with me. And I know, initially, when I first have a new physician or nurse practitioner, it's hard to have that trust right away. So I would say, just having that care and sympathy towards me and the time, not feeling like I'm being rushed during my assessment with them. Yeah. So asking any pertinent questions and making me feel like my information is not shared with other people, and that I can feel comfortable with them. So maybe that reassurance as well. Yeah.*

Excerpt - Document: 142 Respect Interview- cleaned.docx, Position: 3098-4092

*People have answered that question differently. Some people bring up examples like reminders or policies that their hospital or clinic might have in place to remind them to come in or things like that. Privacy is a big one. So people answer differently. If you don't have anything to answer, no problem. We can move on.*

*S2 07:00 Okay. Yeah, I'm trying to think. So yes, I definitely-- I do appreciate because it's like we are so caught in our busy day-to-day lives, that it's nice to have a reminder of any follow-ups that may be needed. For example, when I went to my annual exam a few months ago and that was my first time meeting with [inaudible] physicians besides the doctor I'm with, I had questions regarding some cancer history within my family. So the nurse practitioner [inaudible] had provided some resources. And I appreciated that, knowing that I could rely on some medical professionals to provide information in regards to what my results were and how I can get more information.*

Excerpt - Document: 151 Respect Interview - SP English translation - cleaned.docx, Position: 1264-1489

*could you give me some examples of the things that make you feel respected in your medical care?*

*S2 03:22 I have good treatment, and I’m completely satisfied with the way they have treated me. They’ve given me a lot of help.*

Excerpt - Document: 151 Respect Interview - SP English translation - cleaned.docx, Position: 1499-2164

*And, is there a more specific moment that you can think of, when you felt respected with your medical care? Maybe something that one of the providers did or a specific person in the clinic?*

*S2 03:54 No, not at all. The times that I’ve gone, I’ve been, very good.*

*S1 04:04 So you don't have any examples or things you can think about right now, that you’d said, "Yes, I feel very respected,” besides helping you and receiving good treatment?*

*S2 04:17 I think that’s an example. I don’t know what else I could say.*

*S1 04:24 No, that’s fine.*

*S2 04:24 What I’ve needed, they have given, and it’s been a very, very special treatment. I don’t know what else I can say.*

Excerpt - Document: 151 Respect Interview - SP English translation - cleaned.docx, Position: 3568-4094

*So, could you imagine something that would make you feel that you are disrespected? Or maybe you’ve been told that by one of your friends or your family. Can you think or imagine an example? If you can.*

*S2 07:06 I think that they lack respect if they don't pay attention to us, if they can't speak to us in our language, if they don't give us immediate help. I think that may be disrespectful, but it didn't happen to me, and I didn't / (hadn’t) realize(d) that (this had happened to me) they had [inaudible] me. I don’t know.*

Excerpt - Document: 151 Respect Interview - SP English translation - cleaned.docx, Position: 4105-4729

*That’s a good point that sometimes they aren’t showing you all the respect, because sometimes you don’t notice it. But you say that you haven’t noticed it, and you haven’t felt a lack of respect.*

*S2 07:47 No, no sir, they have taken great care of me. The whole process has been in my language. There’s been no obstacle for me to communicate with them or they with me, because they have the possibilities to have the interpreter. They speak in Spanish or immediately ask me, “Do you want English? Do you want Spanish? Do you want this to come out?” They are always with the patient, who is satisfied with the given treatment.*

Excerpt - Document: 156 Respect Interview-cleaned.docx, Position: 2856-3619

*And so I want to ask you if you could give me some examples of the sorts of things that might make you feel a lack of respect in your medical care.*

*S2 07:04 I feel like with doctors, it's almost like they think they're better, the ones that I've hand anyways. There's been a few that just act like they know for sure when there's not really necessarily a certain answer for something, so I don't like that. And then not fully being aware of somebody's medical history--*

*S1 07:49 On the doctor's end?*

*S2 07:52 Yeah. That they don't double-check a chart to see if everything's the same before they actually talk to you or something. But I think the biggest one is just not talking to the patient like they're an actual person. It's almost like being talked down to.*

Excerpt - Document: 158 Respect Interview - SP English translation - cleaned.docx, Position: 1200-1515

*Could you give me some examples of the things that make you feel respected in your medical care?*

*S2 03:21 I like it a lot, and I go to my clinic because they respect my schedule, they respect my privacy. I’m at ease because they serve me well, they are respectful to me. I’m comfortable, due to many things, really.*

Excerpt - Document: 158 Respect Interview - SP English translation - cleaned.docx, Position: 5048-5537

*So, if there were, what can you tell me about the policies that a medical institution has? For example, what kind of policies would show disrespect?*

*S2 08:59 Do you mean, about how they could be disrespectful to me?*

*S1 09:04 Yes.*

*S2 09:08 I think that maybe if they don't take good care of me, and that if I ask them for something, and they don't give it to me. Or some medicine or that I go, and they don't have the medicine that I need at the time. That, for me, would be disrespectful.*

Excerpt - Document: 158 Respect Interview - SP English translation - cleaned.docx, Position: 19673-20127

*Can you tell me a little more, if you can, about why you answered five to all these questions?*

*S2 30:01 Because where I go, to my clinics, they attend to me well, they treat me well, they’re friendly. Everything about the Charm Study was very good. I understood everything I had to do. As I say, I did it here at home. Then, when they called me to give me the results, they behaved very well toward me. There was a lot of respect and I understood things.*

Excerpt - Document: 128 Respect Interview - SP English translation - cleaned.docx, Position: 1964-2471

*Can you think of a specific moment when you felt respected? Because what you told me was more in general, but I want to know if you have an example that you can give me of when you felt respected.*

*S2 05:31 Once in a while I visit my rheumatologist. He could listen to my needs, but moreover, he doesn’t speak my language. But he is attentive to my needs and that’s a moment when I love going to the clinic, but not receiving the attention he provides as a doctor makes me feel very dissatisfied as a client.*

Excerpt - Document: 174 Respect Interview - SP English translation - cleaned.docx, Position: 907-1738

*I’d like to step back and ask you to think about your experiences with medical care, including the doctors and clinics that you visit. So, could you give me some examples of the things that make you feel respected in your medical care?*

*S2 03:00 Well, they attend to me, they explain the problem well, about what I'm going through. They always treat me with respect, I mean, they always resolve my doubts about what I have. I mean, if I have any questions, they always provide me with a good answer.*

*S1 03:30 Okay. And, was there ever a moment when you felt respect-- sorry you already told me that with your answer. But, what can you tell me about the providers or the doctors? Have they done something different than-- even at the clinic or would you say that everyone who works at the clinic acts the same?*

*S2 03:57 Everyone.*

Excerpt - Document: 113 Respect Interview_cleaned.docx, Position: 398-1146

*So could you give me some examples of the sorts of things that make you feel respected in your medical care?*

*S2 02:46 Well, I recently had a stroke, so I've been going to see a doctor often. I like it when they're truthful and honest and when they don't beat around the bush. I feel more respected when people are just honest and look you straight in the eye and tell you what the conditions or what their concerns or what you need to do get better. Instead of going around the bush and saying, "Well, maybe you could try this," or, "Maybe if you try that." I'd rather just be frank and honest and I find that more respectful than beating around the bush. But I haven't had a bad experience. I do find [the healthcare system to be] very respectful.*

Excerpt - Document: 146 Respect Interview- cleaned.docx, Position: 426-4844

*So with that in mind, can you give me some examples of the sorts of things that make you feel respected in your medical care?*

*S2 03:05 Well, my personal experience is the ones that actually listen to what my concerns are, not what they think my concerns are, the ones that actually take in why I'm there. And then the ones that actually take that and - how do I say this? - try to help solve the issue instead of just plopping a Band-Aid on it and saying, "Oh, it's just this. You're fine," when then I have to go back and get a second or third opinion. And the second and third opinion don't match the first opinion, and they're calling the doctor idiot or whatever.*

*S1 03:52 Okay. So could you expand on that a little bit, and I want to just ask what it is about that that makes you feel respected.*

*S2 04:02 Well, for me, personally, I've battled some ovary cyst issues the last few years. We’re not sure if it's my IUD or not, but it's caused other symptoms. So my biggest concern was I wanted my IUD out. My first and second doctor, including my OB, were like, "No. That's not it. You're just gaining weight just because you're not active," and this and that. I went and saw a new doctor, same [healthcare institution] clinic, and a new OB, and they go, "Yeah. You're not as active as you were, but your IUD is clearly playing a role in your problem." Then they asked me-- the new two doctors asked me, "Okay. Well, what are your other symptoms? What else do you have going on?" But the second set of doctors actually had the time to go through my notes, my hospital notes, everything, and not notice that, "Hey, you've had a miscarriage in the last year. Hey, you showed up at urgent care bleeding profusely with a cyst ruptured. We need to get you in now and figure out what's going on." It was those second two doctors. They're the ones that said, "We need the ultrasound immediately. We need to do the Pap smear immediately. We need to do all these tests immediately."*

*S2 05:25 Well, when I called in to-- on the ultrasound schedule, because it was put in as an emergency ultrasound, they told me it was going to be three to six weeks for me to get seen. Okay, fine. Let's get it in. We got in, I think, four weeks after the initial rupture of my cyst that we thought was a miscarriage, but luckily, that one wasn't a miscarriage. That one was an actual cyst that ruptured. It just felt like I was going into labor. The doctor called me back the next day, the new doctor. My new OB called me back the next day and said, "This is unacceptable. I cannot believe they're making you wait four weeks to get seen. We need to have you go in now." Later that afternoon, I got a phone call from the ultrasound department because there's only two on the east side that I can go to. Because of [healthcare institution], we're limited to where we can go. She was able to get me in the next day. Because of her due diligence, because of her actually listening to me and taking the time to read over my notes, she understood that this wasn't just me having cramps, and the weight gain wasn't just over a period of time. This was a rapid weight gain.*

*S2 06:38 Everything else about me was healthy, but the rapid weight gain, the fatigue, all of my symptoms were like, "No. Something else is going on. I honestly think it is your IUD. We need to have that ultrasound to figure out what's going on." If it wasn't for her, I would've never had the ultrasound done. And then still to this day, two and a half years later, still battling the cysts. So, for me, it's one of those things like-- and, of course, that doctor ended leaving on maternity leave and never came back. So now I have a new doctor that's just like my first doctor, and it's like, "Oh, well, it's not a big deal. You are in your mid-30s. You're healthy. You're overweight, but, yeah, you're healthy." I'm like, "No. There's something going on. I know my body. There's clearly something going on. It shows on the ultrasound." But the new doctor is just as bad as the first one. The second doctor was fantastic, absolutely fantastic. I'm sad she's gone. She is the first one to actually truly listen to what my concerns were. She was talking like a friend, not like a professional.*

*S1 07:53 Okay. So that's really helpful. It sounds like listening to the concerns, but also, there being a follow-up with the urgency and action and it being timely as well. Does that sound like what--?*

*S2 08:05 Yes.*

Excerpt - Document: 146 Respect Interview- cleaned.docx, Position: 6873-8590

*So now I'd kind of just like to shift it to the other side and ask, can you give me any examples of the sorts of things that make you feel a lack of respect in your medical care? Anything in particular you can think of, whether it was a time you felt a lack of respect--*

*S2 10:53 Just not listening. When someone tells you-- and you've been their patient for like five, six years, and you tell them, "Hey, something's wrong. I've never had this happen before. This is new." And they're like, "Oh, it's just this." I pulled some muscles in my shoulder last weekend, and the pain was unbearable, so I finally took myself to urgent care. She didn't even so much as touch my shoulder. She had me move my arms up and down and said, "Okay. You have a pulled muscle and a partial dislocation. Here's some pain medicine. Have a good day." And I'm like, "You didn't even touch my shoulder. You didn't hear a word I said. You didn't ask me what my pain threshold level was. You didn't try to manipulate my shoulder to see which direction it could go. You didn't ask about the car accident," that it turns out this is a sideline of the car accident. She didn't do any of that. So I was like, "Why did I go to urgent care?" And all she did was prescribe me pain pills. I'm like, "I don't want pain pills." She didn't do X-rays. She didn't do ultrasound. She didn't do anything on the shoulder. So how can you tell me it's a partial dislocation when you didn't even touch it?*

*S1 12:06 Okay. So the feeling--*

*S2 12:06 So really, it comes down to--*

*S1 12:08 Not listening and then not taking your concerns seriously, it sounds like. Okay. And, yeah, are there examples that you can think of?*

*S2 12:18 No, not off the top of my head.*

Excerpt - Document: 146 Respect Interview- cleaned.docx, Position: 41751-43143

*And just any quick notes about why you answered those questions that way?*

*S2 52:20 Like I said, I had a great experience with your people. They were fantastic. I didn't feel like I was being-- I didn't feel like I was a test subject. I felt like I was-- like I said, a conversation with a girlfriend or a friend at the coffee shop. I didn't feel like I was being viewed under a microscope so to speak. Whereas the medical field, I trust my chiropractor and my massage therapist and my physical therapist more than I do my actual doctors. But my chiropractor and my physical therapist, I see more partially because of the auto accident. But I feel like I'm not a patient. But they don't say, "Hey, you're a patient here." They're like, "Oh, hey, Cindy's here today," or they come in and they know what's going on. It doesn't matter whether I've been in twice this week or I haven't been in in a year. They still know what's going on. They'll ask questions like, "Oh, hey, how did this show go?" It's like, "That was like six months ago. What are you talking about?" and I'll forget about it. It's like, "Oh, well, you went to this car show, and how did you guys place?" and blah, blah, blah. I mean, I've been going to the same clinic for almost [inaudible] years now, but I don't feel like I'm a patient there. Whereas at the doctors, I feel like a patient. I just want to get in and out, so.*

Excerpt - Document: 133 Respect Interview-cleaned.docx, Position: 3503-3951

*And so could you maybe go into those a little bit more and tell me what it is about that exactly that makes you feel respected?*

*S2 04:28 Yeah. It makes me feel like my time is valued as well. And I know in doctors you have the higher [inaudible] and whatnot, and their time is valuable as well. But just going that extra mile and staying on time or giving me that prompt information, makes me feel like my time or my health issues are top priority.*

Excerpt - Document: 133 Respect Interview-cleaned.docx, Position: 4624-5731

*Now I’d like to kind of go on the opposite side of that and see if there are any examples that you could give me of things that make you feel a lack of respect in your medical care?*

*S2 06:03 Yeah. Again, I don’t have experiences, like I said, I’m generally very healthy. From what I had to deal with [inaudible], for example, I had surgery a while back on my hand. I don’t think I felt disrespected but my doctor didn’t-- I feel like he [inaudible] some portion of it, telling me about the downtime after the procedure, of the surgery, how long I would be down for. I feel like I didn’t get a true understanding of how long I was going to be truly down and not be able to use my hand for the activities that I do daily. So in that sense, I wouldn’t say I feel disrespected or I felt a lack of respect there, but I did feel that they didn’t provide me with enough information to say, “Okay, this is what I want to do.” Or try and get an idea of what I’ll be facing later on. So in that aspect, it’s a little bit of it-- I feel, there was a little bit lack of respect there for my job and my daily activities.*

Excerpt - Document: 157 Respect Interview-cleaned.docx, Position: 1137-1435

*So would you say that communication is a big kind of factor when it comes to feeling respect?*

*S2 04:05 I think so. You maybe might have some weirder questions or something maybe that are not easier to answer-- or ask. And whenever you feel more comfortable, it might be easier to ask those things.*

Excerpt - Document: 157 Respect Interview-cleaned.docx, Position: 2517-3514

*o you kind of touched on some aspects of the next question that I wanted to ask, but could you give me some examples of the sorts of things that would show a lack of respect? And I know that you just mentioned a lack of eye contact or people being in a bad mood. Was there anything else that you can think of?*

*S2 07:15 Let's see. Yeah. I mean, lately, I've just had good experiences, I think, but whenever someone's in a bad mood, you're definitely not sure if they're just having a bad day, or you just kind of feel like they're taking it out on you. You want to be in a good mood when you're discussing your health stuff even if they're having a bad day. And I think just following up to make sure you have any questions afterwards and giving you lots of chances to ask questions. Because sometimes things can be really busy, and you might forget to ask questions or something like that, and that kind of can be frustrating. Well, yeah, just feeling like you're not rushed is probably a big one.*

Excerpt - Document: 157 Respect Interview-cleaned.docx, Position: 4718-5771

*And so thinking a little bit about the policies again, in your opinion, do you think there are any policies that show a lack of respect?*

*S2 10:13 I think it just comes down to pain management, probably, yeah, related to that. It's probably hard for them too. But I was in a car wreck last year, and I couldn't move for two months. I literally couldn't move, but they didn't give me any pain medication. So I don't know. To me, that's kind of a problem with healthcare right now. And then also, I would say, too, maybe the lack of natural remedies. Of course, they always want to prescribe you something that costs a lot, but sometimes there are natural remedies. I don't know. One time I went to the urgent care because my shoulder was hurting really bad, and she was like, "Oh, well, you just need to do this exercise," and that was kind of nice to be able to have a solution that wasn't a pill. Yeah.*

*S1 11:17 So maybe providing you with some alternatives to the normal, like you mentioned, pills and those types of medication.*

*S2 11:28 Yeah, yeah.*

Excerpt - Document: 106 Respect Interview-cleaned.docx, Position: 1624-3117

*are there any maybe specific actions that a medical provider or the nurses or doctors kind of performed, for lack of a better word, that really exemplified respect?*

*S2 04:46 Yeah. For one knowing my case. It was really hard for me when I came in and someone was scribbling on a piece of scrap paper [laughter] and didn't seem to know what was really going on with me. And I'm like, "Oh, my God." It made this appointment-- you should have read up a little bit before you came into the room. So that kind of stuff would be frustrating. And then in the healthcare system I was in, they kind of just scheduled you with whoever was available that day, so I felt like that made it hard too. I didn't have a consistent person that I saw other than the doctor who did my ultrasounds. But he deliberately made the choice to be like, "I'm going to do her ultrasounds. I don't want anyone else doing them." I didn't know at the time that you can do things like that. I just thought you had to go with whoever they gave you. So it was really nice having a consistent person who saw you. And then if someone didn't know you, that they followed up and-- follow-up was huge too. Feeling like, "Okay, this person is going to call me about the results," or they want me to come back in two months, or whatever, to make sure I'm not having these symptoms again. It was more like I had to make that call, so that was just difficult for me to navigate and feel that people really cared about what was happening.*

Excerpt - Document: 106 Respect Interview-cleaned.docx, Position: 4546-5158

*Oh, actually there was one appointment I went to where I was telling my doctor I had pain in my pelvic area and she basically - before I could even answer her - made the decision for me that I did not want to get a pelvic exam done at this time, and that if in the future I want to let her know. Before I could even be like, "Yes," "No," or "Wait, why do we need to do that?" she was just like, "But you just seem like you don't want to do that right now, so if you do in the future just let me know and I can schedule something for you." So I felt like she just made decisions for me without letting me answer.*

Excerpt - Document: 145 Respect Interview-cleaned.docx, Position: 431-1115

*So could you give me some examples of the sorts of things that make you feel respected in your medical care?*

*S2 02:48 I think the number 1 thing that's important to me especially if you're going to see the doctors since I'm someone who doesn't seek out medical care very often is in just knowing that my provider is there and going to listen to all of my concerns. And then not feeling rushed during that time because a lot of the times I go, I know you have another appointment right after. But sometimes it can come off as dismissive. So I like having that at least demeanor that I'm being heard, and that all my questions are being taken seriously and answered thoroughly, I guess.*

Excerpt - Document: 145 Respect Interview-cleaned.docx, Position: 1217-1835

*is there anything in terms of your interactions with say the intake staff or the nurses or any of the other staff that you find respectful?*

*S2 03:50 I mean, just really good customer service. I mean, it is kind of customer service in a way. So just being greeted warmly, again, that just kind of active listening from all parties is really important. Patience in case I'm not understanding something correctly or thoroughly. I mean, depending on what's going on, if it's a nurse or maybe a phlebotomist or lab tech or whatever just being able to have clear communication about what's going on and why that's happening.*

**Quality of care**: Providing good medical care (i.e., the technical proficiency of one's care); not making procedural errors

Excerpt - Document: 114 Respect Interview-cleaned.docx, Position: 4928-6829

*And this sort of goes to a couple of the issues that you’ve brought up but I wanted to ask it explicitly. Have you ever felt the lack of respect in the medical setting based on who you are, what you look like, what you believe, any of those sorts of traits?*

*S2 10:23 In a study?*

*S1 10:24 In medical care or in a study, I guess, either way.*

*S2 10:28 This is actually my first study but in medical care, yeah, I have felt sort of ignored. My concerns are kind of brushed off and when I was bringing certain things to the forefront saying, “Hey, I think this may be what my issue is,” they kind of just brushed me off and went, “Well no. I know that’s not a problem. This isn’t this.” They didn’t do any tests. I mean, there was plenty of times that I left the ER after experiencing severe stomach pain and they’re just like, “Well, we don’t know what the issue is. Go home.” And of course, that was when I didn’t have insurance. I felt that when I didn’t have insurance, I was basically just, I guess, a burden to them at that point without a doubt. I ended up with $6000 of medical bills because I had to go back into the ER multiple times to get a skin infection taken care of. The first time I actually went into the ER, I had gone in with a temperature of 106 degrees and I waited in the empty waiting room for four hours in the morning. And when they brought me back, my temperature had already dropped and when I told them what it had been, he’s like, “Really, are you sure you didn’t read it wrong?” So I was a little upset. But then they also didn’t run a proper test and so they didn’t find out what it was that was giving me the infection so they didn’t give me the proper antibiotics.*

*And then the next time they gave me the wrong antibiotics that didn’t have good kidney penetration so it wasn’t getting rid of the kidney infection. So I felt there was a lot of oversight in that instance.*

Excerpt - Document: 114 Respect Interview-cleaned.docx, Position: 21190-22239

*And then on a scale of one to five, and going back to that same scale, how much did you stay you trust medical care in general?*

*S2 34:11 It’s kind of a difficult question because it kind of comes back to that depending upon the doctor.*

*S1 34:17 Right, right.*

*S2 34:22 For myself what I’ve experienced in my recent years, I would say a five. But concerning family members in the last two years, I would say honestly two. Things went very, very poorly with my father-in-law with esophageal cancer.*

*S1 35:07 I’m sorry to hear that.*

*S2 35:10 Yeah. And family friends who were going to the same doctor have also either passed or have had basically nothing done for them. So I’d say an instance for that doctor, it’s a very low number. He didn’t listen, he people feel stupid, and never explained anything. And generally, I felt he wasn’t as aggressive with his treatment as he should have been.*

*S1 35:57 Okay. So it sounds like there’s a lot of-- like trust sounds like it’s a really personal dependence on the individual person and an individual doctor.*

Excerpt - Document: 101 Respect Interview-cleaned.docx, Position: 394-1004

*So could you give me some examples of the sort of things that make you feel respected in your medical care?*

*S2 02:53 I guess just when they actually diagnose the problem right makes me feel respected. But it’s kind of one of those things where sometimes it feels like they’re guessing more than they’re actually diagnosing. Sometimes I may just feel-- when you feel like they’re guessing, you don’t really feel that much respect than them trying to take the time. But there are a lot of doctors out there-- my doctor now takes a lot of time and a lot of care in trying to figure out what’s really going on, so.*

Excerpt - Document: 101 Respect Interview-cleaned.docx, Position: 2498-3444

*And then, so what about the policies that the medical institution has in place? What kind of policies do you think show respect?*

*S2 06:03 I mean, I think now-- I mean, recently, I had [surgery], and I didn’t have insurance. So with them being able to actually still go in and do what they needed to do and not treat me, yeah, like a leper, pretty much, because I didn’t have insurance and still do everything that they would’ve normally done, that was pretty great. I was pretty freaked out when I had to go in and didn’t know what was going to happen since I didn’t have insurance at the time. So that policy of being able to still take care of the patient and take care of what they needed to do. And then I had people come in and help me get Medicaid set up and everything, so it was all taken care of anyway. And I had to stay for four days, so their staff treated me amazingly, made sure everything was taken care of with me and my wife, so.*

Excerpt - Document: 101 Respect Interview-cleaned.docx, Position: 5720-6814

*has there ever been an encounter in the past with a previous doctor or someone else where you experienced a lack of respect?*

*S2 10:42 Yeah. I mean, I’ve had some emergency room doctors in the past where you go in and it feels like they’re just trying to kick you out. They don’t want to really deal with your issues of what’s really going on. So I have had that where it just seems like they got so much going on that they try to just quickly diagnose you. I mean, the first time I went in for the [issue related to my surgery], for example, they tried to diagnose it as just [being related to a preexisting condition], and they wouldn’t do a second ultrasound. They ended up sending me home with some Zantac. And then two days later, I had to go back to the emergency room, and they finally decided that, yeah, [I needed to have surgery]. Even though the first time I was in, the CAT scan showed [the affected area], and the doctor said that they knew that it needed to be taken out. But the surgeon said that they couldn’t see clearly enough on the ultrasound, so they weren’t going to do it.*

Excerpt - Document: 154 Respect Interview-cleaned.docx, Position: 3337-4997

*And have you ever felt a lack of respect in the medical setting based on who you are or how you look or what you believe?*

*S2 07:08 If it was, it never, to me, connected that that was the reason why. I've been reading more stuff, and more things are coming out, and I've read articles on stuff. I'm like, "Huh, huh." But, yeah, I never walked away thinking, "Oh, they were dismissive." Well, no, no, wait, I take that back. Yes, there have been interactions where it was like this idiot man doctor - excuse me - done some things that-- I know my body as a woman. There have been a couple of interactions like that, yes.*

*[silence]*

*S1 08:03 All right. And if you're okay with it, would you mind kind of talking to me about that experience?*

*S2 08:14 Okay. Hold on. Let me blow my nose.*

*S1 08:16 Oh, sure. Yeah, no problem.*

*S2 08:19 I've just gone into the office here. Years ago-- I mean, this is TMI too, but I didn't have any health insurance, so my mother called the advising nurse on her health insurance and asked if they would speak to me because I was bleeding anally quite a lot. I've had digestive issues off and on for years. But I spoke to a doctor on the phone, and he kept saying, "Are you sure you're not on your period? You're probably just on your period." And I'm like, "I know the difference between my vagina and my anus." I'm like in my mid-20s at this point. I think I know the difference. So, yeah, it still makes me mad when I think about it because I'm just like, "You're an idiot. Listen to what I'm saying."*

*S1 09:11 Yeah, no. So that's really one of those unfortunately terrible examples of doctors just not listening to patients. Yeah.*

Excerpt - Document: 154 Respect Interview-cleaned.docx, Position: 10888-11271

*That's why I was like-- when I earlier said that I have a lot of experience with the medical community. It's not always me. It's that I was a caregiver for years. And some of my worst experience was dealing as an advocate and a caregiver for her and not necessarily myself. One time, the hospital lost her.*

*S1 17:42 I'm sorry?*

*S2 17:42 Another time, they lost her clothes [laughter].*

Excerpt - Document: 132 Respect Interview-cleaned.docx, Position: 2791-4830

*What about policies that a medical institution has in place? Can you think of any policies that would show respect or show disrespect, by chance?*

*S2 06:14 Well, I know that a lot of offices and people in general are trying to be more inclusive and make sure like, “Okay. We’re for everybody, not just a certain group of people.” I feel like that’s important. Because there have been times in the past where I didn’t have health insurance. And I had an injury. And they just said, “Well, stay off of it.” Stay off of it, that’s all they told me. And quite a few years later, I had insurance at some point. I went in and got my knee checked for that injury for that [inaudible]. And they just said, “Oh, you have an ACL tear here that was quite old. And it’s so old that there’s nothing we can do about it. Because it’s just completely gone now.” And I knew at that moment that that injury I had was an ACL tear that could have been repaired. But because I didn’t have health insurance, they didn’t take the time to look at me. They just brushed me off. So I feel like-- I think I’m tying up two different things here. It’s getting confusing in my own head. To not be excluded because of what you look like or what race you are, stuff like that, those things, I find those respectful. And I appreciate those. I am a Caucasian female. So basically, I haven’t experienced some of that. But there are other people who have. But then, also, I think policies that say you deserve to be treated regardless of whether you have the money to pay for it or not-- and I feel like that that needs be more-- it’s stated, yet not always followed. Does that make sense?*

*S1 08:13 Yeah, yeah. Okay.*

*S2 08:15 So I guess it’s an unspoken thing that says, “We’re going to treat people who we know can pay rather than those who don’t.” That’s a disrespectful policy because every life has value. And if somebody can’t afford to pay for it does not mean that they shouldn’t have a chance to live or have a knee that’s not totally whacked out the rest of her life.*

Excerpt - Document: 110 Respect Interview_cleaned.docx, Position: 1018-1304

*And what is it about that listening and understanding that makes you feel respected?*

*S2 03:42 To know that I'm being heard and that the issues will be addressed and things will be done to come up with a reason why something is happening, or a good treatment plan for whatever comes up.*

Excerpt - Document: 136 Respect Interview-cleaned.docx, Position: 4455-4873

*Or what if they give me the wrong medication or something that I'm allergic to, or my child is allergic to, that would be a really big issue. And I would be furious [laughter]. Or just being brushed off or not getting my questions answered. The would be pretty frustrating because I mean that's the main reason that I go in to get answers and to get better, and if nobody tells me anything well, then how can I, right?*

Excerpt - Document: 153 Respect Interview - SP English translation - cleaned.docx, Position: 18587-19306

*Once again, could you tell me a little more about why you answered these questions about trust in this way? I noticed that we’ve had a bit more trust in general compared to respect.*

*S2 03:03 Oh, my answer is because when I, I detected something wrong, I went to the doctor and they told me that there was nothing wrong with me; I went back to the doctor and they ran some tests on me, they did a biopsy, and they told me that it wasn’t [illness]. So then I was doing really well, until later when they operated on me and it turns out that I do have [illness], or in other words, then they said I did. So they don’t know, either; they’re really not sure when they’re running a test on you what’s really happening to you.*

Excerpt - Document: 122 Respect Interview-cleaned.docx, Position: 1853-3455

*o now I kind of want to flip those questions on their head and ask you to give me some examples of the sorts of things that make you feel a lack of respect when it comes to your medical care?*

*S2 00:00 Recording back up.*

*S1 00:01 I do. All right. And we should be getting on. I’m so sorry about that.*

*S2 00:06 That’s okay. It happens. Well, I mean, I guess I can-- one thing that happened recently was actually with my son was I had taken him in for an urgency care appointment. And I kind of felt like the doctor just came in and was kind of like, “Okay. What’s the problem?” I mean, I swear he probably spent-- and actually I think he was a PA but I think he probably spent less than five minutes in the room and threw some antibiotics at us and out the door, we went. So, yeah. So that would be something as of recent that happened with my son. But as far as my care, I don’t go to the doctor, I mean, a whole lot. I’m just not a-- I don’t go unless there’s a really good reason I guess and so I go to the doctor to maybe three times a year max. So I feel really fortunate with the care team that I have that everything always is addressed and I feel respected and, so.*

*S1 01:26 Okay. So it sounds like from that example that you gave, it’s kind of the opposite of the things that you had mentioned with the first question. In that instance, it did feel like you’re being rushed, maybe didn’t feel like the doctor was listening and that the decision was kind of made for you without really consulting you. Does that sound, right?*

*S2 01:47 Right. Yeah, yeah. Exactly. There was no empathy or anything.*

Excerpt - Document: 123 Respect Interview-cleaned.docx, Position: 4119-8710

*you sort of alluded to this but can you give some examples of times when you felt a lack of respect in medical care?*

*S2 06:52 Yeah. So on my journey with stomach stuff, when I was going in-- I mean, I was throwing up in my sleep and so I went into the examination room. So we don’t know if they’re primary care and the woman I saw, I said, “I’m throwing up almost twice a week in sleeping mode.” And she’s like, “Well, you only have two episodes. That’s good.” I’m like, “I don’t think that’s true.” You know, I get it. I’m a [occupation], I’m not a doctor but that doesn’t make sense because you can die when you’re hearing that phrase, that’s disrespect. And so she was just like, “No. Two episodes is good.” And I asked to talk to a specialist and she said I didn’t need to see a specialist. So she really didn’t validate my feelings nor did she wish my medical desire which I felt was ridiculous. And then, I went back. A week later, once again I was throwing up in my sleep. I went in and saw-- I don’t know if he was a nurse practitioner or something, and he was just like, “What? Some people are thin donuts and some people are fat donuts.” And he actually compared me to like a glazed, sprinkled Mustang donut and was saying that my digestive system is like the center of that donut and if I were just as more compact donut that was more fit, my food would just go through me. But since I was more spread out like that fat sprinkled donut, then I was going to continue to have problems, and I was just more shattered. Then, I said, “You know, as I’ve gone through this journey and I extraordinarily lost a bunch of weight and I’m still working out.” And then I got shin splint. I tore my rotator cuff. I’ve had a series of cortisone shots and all of these things, and I’m kind of afraid to jump back. And your family is like, “Well, a cortisone shot is better than not being fat.” And I think it could probably be two ways.*

*S2 08:50 So again, he was just so quick to jump on the weight bandwagon. He wasn’t listening to my symptomology at all, and he was just like, “Well, you know.” And then, this was like minimize shame and it seemed ridiculous to me also like is that really the best solution our medical providers can give us. It’s like, “Well, you’re obviously in some pain. Just go get another cortisone shot” which is weird because there’s research about cortisone shots that you’re only supposed to have so many and he was just dismissive of it. But then I had a really bad example where I had had a bad pedicure but not a chronic issue. But I had a bad pedicure and I had gotten an ingrown toenail. So I went in and I was telling the provider. They’re like, “Oh, just soak it.” So I did and it didn’t resolve. And so it ended up being where I had to have it removed. And I don’t what he was like a physician’s assistant or something. He had a student come in and said, “Do you mind the assistance?” I’m like, “You know, I really kind of prefer, no offense to you.” Whilst she’s trying to learn and now what has happened is-- so I felt pressured. He said, “She’s a very nice old student.” So I’m like, “Okay. She can assist.” They didn’t know my foot. My foot ended up being-- I don’t know. Well, at least 12 shots in my toe, so the toe ended up being bruised for a week. They removed half of my toenail. And because students used like a little bottlebrush with the root. They killed the toenail root. My toenail now can only grow about a quarter inch out and then it dies because they’ve permanently damaged my root. So I don’t know. Well, it wasn’t respectful. I didn’t want the student. They probably shouldn’t ask if a student is right there because it puts the patient in a precarious spot. When I said no, he didn’t accept no, “Oh, common. She’s just trying to learn; I’ll be here.” And then, she failed with that and now I have a permanent like-- does it kill me? No.*

*S2 10:52 But I’ve had to have that toenail removed a couple of times since then. I never had ingrown toenails before. It wasn’t like I had a chronic problem where I needed that level of treatment. And now, it literally dies off and it turns like almost a bluish color. It’s unsightly that doesn’t kill me but had he just listened to me in the first place perhaps I wouldn’t have this and I actually asked to see a specialist for it. And my providers said I didn’t need to.*

*S1 11:25 Yikes. Well, I’m sorry to hear you have so many examples of-- that sounds rough so I’m sorry you had to go through that.*

*S2 11:35 Like if I’m old, over 45 years, this wouldn’t have been bad, but I’m [in my early 30s].*

Excerpt - Document: 142 Respect Interview- cleaned.docx, Position: 4251-5481

*can you think of any examples of things that have made you feel a lack of respect in your medical care? Any examples?*

*S2 08:26 I can't say have felt the need to receive a lot of medical attention. I feel like my health is usually just basic needs. I haven't felt like being in the hospital or anything, I've had disrespect. Yeah. I would say that most--*

*S1 08:50 Even if you haven't-- even if you haven't experienced it directly, can you think of anything that might make you feel disrespected or that maybe that you know have happened to others that you feel like might be examples of disrespectful things to you?*

*S2 09:11 Yeah. So I actually work in the health field myself. So I would say, I've witnessed in hospital settings more so about-- to me, I feel like with seeing the hospital setting is more acute care versus the care for long-term care. If that makes sense? So it's usually short-term fixes rather than preventing further hospitalizations for certain patients. So in that regard, I guess, I've seen some disrespect when some of these people are getting readmitted to the hospital. I just feel like some of these patients are being discharged too quickly, probably because they're not getting the most optimal care.*

Excerpt - Document: 149 Respect Interview - SP English Translation - cleaned.docx, Position: 14106-16610

*People who participate in the study do not have to do anything, but researchers just want to have permission to have access to these records, and this is because they want to see if the medications have different effects which affect people's health.*

*S2 20:57 No, that's very bad. Without you knowing, to give medications, and you don’t know anything. Well that's it - it's very bad, because you don't even know what they’re doing to you. No, for example, [inaudible](my example), since I have [illness], the doctor prescribed me a medication. It didn't do anything for me at the beginning. Then, my chest ached, so then she changed the medication to another one and started telling me to take four tablets in the morning. So, I saw that [inaudible] once. I remember that I took it once like that, but no, those tablets, kind of like giving me a lot of medicine in one day. I mean, in the morning four tablets, and my chest felt really bad. So, I didn't say anything to the doctor, I just dropped it down to two, instead of taking four. I took two because four, like, like my chest hurts. I mean, I mean, why do they prescribe so much medicine in one day? That is, all at once. Do you know what I mean?*

*S1 22:30 Yes. Yes. Yes.*

*S2 22:31 When she knows. I mean, I’m taking [illness] pills, and then she prescribed me-- the medicine was increased by two more tablets and I felt bad. I mean, why? When I had to drop it down to two pills. Do you know what I mean? I mean, I say why? [Inaudible] (she) doesn’t know or don’t know, in that aspect, I don’t know why. Do you get me? I mean, that’s wrong. When talking with her, either she doesn’t know or will just hit or miss. But, so much medicine is not right. I mean, without knowing what reaction you’ll have. Do you know what I mean?*

*S1 23:24 Well, yes as you said--*

*S2 23:27 And, I mean, at the moment that I-- that is, [inaudible] (its possible that I) that they-- as you’re telling me that they research and all that without the person knowing. Do you know what I mean? I mean, she didn’t know if I were to feel bad with four tablets all at once, in one day. I mean, I told her, "My chest hurts when I take this medicine." And I took one in the morning and one in the afternoon. My chest felt bad. Then she changed the medication, but taking four tablets at once, that was the worst. I mean, no, I don't know what's up. That's why I’m telling you. Sometimes you take things without [inaudible](?). Then it will happen to you. Do you know what I mean?*

Excerpt - Document: 149 Respect Interview - SP English Translation - cleaned.docx, Position: 22368-23511

*Now, could you tell me a little more about why you answered those questions about respect in this way? For example, you said four. Four and then five. So it seems that you feel more respected in the Charm Study compared to medical care.*

*S2 34:40 No, how can I tell you? I mean, I gave it a four, four because then, well, not just because - how can I say it? Because I had an experience when they gave me [type of therapy]. Which one would that have been? The [number], [inaudible] (that is how it was) since they did the [number] [type of therapy] in my right arm. Well [inaudible](how) that experience, because when they gave me [type of therapy], I realized that it was done to me like - they didn't put it into my vein very well. Either it moved or I don't know, and it made, like a bump in my arm. And so, there, I had, like an experience. Do you know what I mean? I mean, I felt, how can I say it? I felt uncomfortable because it had never happened to me. That time it happened to me, and it affected my arm. Do you know what I mean? I mean, that's why, that's why I gave those numbers.*

*S1 36:03 Okay.*

*S2 36:03 Due to that bad experience.*

Excerpt - Document: 151 Respect Interview - SP English translation - cleaned.docx, Position: 1264-1489

*could you give me some examples of the things that make you feel respected in your medical care?*

*S2 03:22 I have good treatment, and I’m completely satisfied with the way they have treated me. They’ve given me a lot of help.*

Excerpt - Document: 151 Respect Interview - SP English translation - cleaned.docx, Position: 1499-2164

*And, is there a more specific moment that you can think of, when you felt respected with your medical care? Maybe something that one of the providers did or a specific person in the clinic?*

*S2 03:54 No, not at all. The times that I’ve gone, I’ve been, very good.*

*S1 04:04 So you don't have any examples or things you can think about right now, that you’d said, "Yes, I feel very respected,” besides helping you and receiving good treatment?*

*S2 04:17 I think that’s an example. I don’t know what else I could say.*

*S1 04:24 No, that’s fine.*

*S2 04:24 What I’ve needed, they have given, and it’s been a very, very special treatment. I don’t know what else I can say.*

Excerpt - Document: 108 Respect Interview-cleaned.docx, Position: 3962-4686

*Are there other things—actually, I guess you said that both of those examples made you less interested in engaging with medical care going forward. I’m curious if you could say a little bit more about that.*

*S2 07:51 I mean, in general, I guess I don’t have a super high opinion of most medical care providers. I mean, it’s not necessarily the individual but I just feel like in our culture, it’s really driven by money a lot and by insurance and that people aren’t always getting the best care because either the care providers are overworked. They don’t have enough time to really sit and listen to what’s going on to really know who they’re dealing with. It’s just sort of in and out, and so-- what was the question again?*

Excerpt - Document: 108 Respect Interview-cleaned.docx, Position: 4905-5345

*When you go in and they like rather than knowing who you are and why you’ve made a decision, or what education you’ve gotten before you made a decision, or like really listening to where you’re coming from. It’s just like blanket information that they give to every person which really is-- because we don’t have the time to get to know you or remember you because they’re seeing so many people and just the whole system is like overworked.*

Excerpt - Document: 156 Respect Interview-cleaned.docx, Position: 4212-4802

*And again, we've been quite a bit about the doctors themselves, but is there anything else maybe on the medical staff with the nurses and the intake staff? You mentioned earlier the problem of rushing. So is there anything else that might come off as a lack of respect that you can think of?*

*S2 09:52 Yeah. I don't know. It wasn't intake. It was, I think, a nurse, and they were slow to give me my pain medication when I was in the hospital. And so they were like 30 minutes late multiple times, and I would have to call them to remind them, and that made me lose a little respect for them.*

Excerpt - Document: 158 Respect Interview - SP English translation - cleaned.docx, Position: 5048-5537

*So, if there were, what can you tell me about the policies that a medical institution has? For example, what kind of policies would show disrespect?*

*S2 08:59 Do you mean, about how they could be disrespectful to me?*

*S1 09:04 Yes.*

*S2 09:08 I think that maybe if they don't take good care of me, and that if I ask them for something, and they don't give it to me. Or some medicine or that I go, and they don't have the medicine that I need at the time. That, for me, would be disrespectful.*

Excerpt - Document: 128 Respect Interview - SP English translation - cleaned.docx, Position: 4195-5425

*are there things that people or the clinic have done, or can you think of examples that make you feel disrespected?*

*S2 08:57 Yes. There have been many occasions, at least with my primary doctor, when I’ve felt really disappointed when-- sometimes I don’t go in for three years at a time because it’s so frustrating to meet with a doctor to whom I’ve come to with some discomfort or some concern and he tells me, “So what do you think is wrong?” If I’ve come for a consultation, it’s because I want him to investigate it, to figure out what’s wrong with me. And the fact that my illness might progress and they refuse to do studies or they don’t want to hear my needs, then that’s when I see that they aren’t valuing me as a person, nor my illness, my needs, and that’s when I feel really badly.*

*S1 10:00 Okay. It seems like you tell him that you have a problem but the doctor just says, “Don’t worry, I think it’s something minor,” or, “You don’t have to worry about it.” Sometimes it sounds like maybe in reality it could be something more severe but they don’t treat it with, I don’t know, the attention that you want to have.*

*S2 10:24 It’s not the attention that I expected or needed, and they are not providing me what I need.*

Excerpt - Document: 116 Respect Interview-cleaned.docx, Position: 2063-3190

*So now I want to kind of flip that larger question and ask you if you could give me some examples of the sorts of things that might make you feel a lack of respect from your medical care?*

*S2 05:56 I think that would be brushing off patient concerns, not really taking much time or thought into something they’re concerned about. I’m trying to think. I know one of my coworkers that I work with has had issues with her iron deficiency and such. And she’s tried to bring them up with her doctor and get some testing for it, when she found out one of the blood panels they did didn’t even search for the type of deficiency that she was curious about, but they just did that one and told her, no, she was fine, that sort of thing.*

*S1 06:41 I see. Okay. Huh.*

*S2 06:44 Yeah. Yeah, that sort of thing with the doctors and such not taking into consideration patient concerns. I’ve had other friends of mine that have had-- like they've been kind of dismissed of health concerns just because they’re overweight, like, ″You just need to lose weight and you’ll get better.″ Those comments were basically made. Yeah, stuff like that.*

Excerpt - Document: 146 Respect Interview- cleaned.docx, Position: 426-4844

*So with that in mind, can you give me some examples of the sorts of things that make you feel respected in your medical care?*

*S2 03:05 Well, my personal experience is the ones that actually listen to what my concerns are, not what they think my concerns are, the ones that actually take in why I'm there. And then the ones that actually take that and - how do I say this? - try to help solve the issue instead of just plopping a Band-Aid on it and saying, "Oh, it's just this. You're fine," when then I have to go back and get a second or third opinion. And the second and third opinion don't match the first opinion, and they're calling the doctor idiot or whatever.*

*S1 03:52 Okay. So could you expand on that a little bit, and I want to just ask what it is about that that makes you feel respected.*

*S2 04:02	Well, for me, personally, I've battled some ovary cyst issues the last few years. We’re not sure if it's my IUD or not, but it's caused other symptoms. So my biggest concern was I wanted my IUD out. My first and second doctor, including my OB, were like, "No. That's not it. You're just gaining weight just because you're not active," and this and that. I went and saw a new doctor, same [healthcare institution] clinic, and a new OB, and they go, "Yeah. You're not as active as you were, but your IUD is clearly playing a role in your problem." Then they asked me-- the new two doctors asked me, "Okay. Well, what are your other symptoms? What else do you have going on?" But the second set of doctors actually had the time to go through my notes, my hospital notes, everything, and not notice that, "Hey, you've had a miscarriage in the last year. Hey, you showed up at urgent care bleeding profusely with a cyst ruptured. We need to get you in now and figure out what's going on." It was those second two doctors. They're the ones that said, "We need the ultrasound immediately. We need to do the Pap smear immediately. We need to do all these tests immediately."*

*S2 05:25	Well, when I called in to-- on the ultrasound schedule, because it was put in as an emergency ultrasound, they told me it was going to be three to six weeks for me to get seen. Okay, fine. Let's get it in. We got in, I think, four weeks after the initial rupture of my cyst that we thought was a miscarriage, but luckily, that one wasn't a miscarriage. That one was an actual cyst that ruptured. It just felt like I was going into labor. The doctor called me back the next day, the new doctor. My new OB called me back the next day and said, "This is unacceptable. I cannot believe they're making you wait four weeks to get seen. We need to have you go in now." Later that afternoon, I got a phone call from the ultrasound department because there's only two on the east side that I can go to. Because of [healthcare institution], we're limited to where we can go. She was able to get me in the next day. Because of her due diligence, because of her actually listening to me and taking the time to read over my notes, she understood that this wasn't just me having cramps, and the weight gain wasn't just over a period of time. This was a rapid weight gain.*

*S2 06:38	Everything else about me was healthy, but the rapid weight gain, the fatigue, all of my symptoms were like, "No. Something else is going on. I honestly think it is your IUD. We need to have that ultrasound to figure out what's going on." If it wasn't for her, I would've never had the ultrasound done. And then still to this day, two and a half years later, still battling the cysts. So, for me, it's one of those things like-- and, of course, that doctor ended leaving on maternity leave and never came back. So now I have a new doctor that's just like my first doctor, and it's like, "Oh, well, it's not a big deal. You are in your mid-30s. You're healthy. You're overweight, but, yeah, you're healthy." I'm like, "No. There's something going on. I know my body. There's clearly something going on. It shows on the ultrasound." But the new doctor is just as bad as the first one. The second doctor was fantastic, absolutely fantastic. I'm sad she's gone. She is the first one to actually truly listen to what my concerns were. She was talking like a friend, not like a professional.*

*S1 07:53	Okay. So that's really helpful. It sounds like listening to the concerns, but also, there being a follow-up with the urgency and action and it being timely as well. Does that sound like what--?*

*S2 08:05	Yes.*

Excerpt - Document: 146 Respect Interview- cleaned.docx, Position: 6873-8590

*So now I'd kind of just like to shift it to the other side and ask, can you give me any examples of the sorts of things that make you feel a lack of respect in your medical care? Anything in particular you can think of, whether it was a time you felt a lack of respect--*

*S2 10:53	Just not listening. When someone tells you-- and you've been their patient for like five, six years, and you tell them, "Hey, something's wrong. I've never had this happen before. This is new." And they're like, "Oh, it's just this." I pulled some muscles in my shoulder last weekend, and the pain was unbearable, so I finally took myself to urgent care. She didn't even so much as touch my shoulder. She had me move my arms up and down and said, "Okay. You have a pulled muscle and a partial dislocation. Here's some pain medicine. Have a good day." And I'm like, "You didn't even touch my shoulder. You didn't hear a word I said. You didn't ask me what my pain threshold level was. You didn't try to manipulate my shoulder to see which direction it could go. You didn't ask about the car accident," that it turns out this is a sideline of the car accident. She didn't do any of that. So I was like, "Why did I go to urgent care?" And all she did was prescribe me pain pills. I'm like, "I don't want pain pills." She didn't do X-rays. She didn't do ultrasound. She didn't do anything on the shoulder. So how can you tell me it's a partial dislocation when you didn't even touch it?*

*S1 12:06	Okay. So the feeling--*

*S2 12:06	So really, it comes down to--*

*S1 12:08	Not listening and then not taking your concerns seriously, it sounds like. Okay. And, yeah, are there examples that you can think of?*

*S2 12:18	No, not off the top of my head.*

Excerpt - Document: 148 Respect Interview - SP English Translation-cleaned.docx, Position: 1487-1888

*Can you perhaps think of specific situations where maybe your friends or family members might have felt respected in their medical care? Not just your experiences, but also maybe other experiences that you’ve heard of--*

*S2 03:57	Well, yes. All the interviews or treatments that I’ve attended, whether they’ve been mine or a family member’s, all of them have been done in a very proper and correct way.*

Excerpt - Document: 133 Respect Interview-cleaned.docx, Position: 25255-25841

*What about the way that you answered for medical care? You gave that a four. Can you talk about why?*

*S2 06:12	Yeah. So I mean, of course, there’s always going to be the one or two occasions where medicine fails you or you’re unhappy with the treatment that you received from any medicine, a dentist or whatnot, no one’s perfect. So I answered a four because I haven’t been above and beyond-- I don’t know. I usually never answer a five. Five is always going to be perfect or whatnot. So I think if ever I have a one [inaudible] or whatnot [laughter], I always answer right below it, so.*

Excerpt - Document: 157 Respect Interview-cleaned.docx, Position: 4718-5771

*And so thinking a little bit about the policies again, in your opinion, do you think there are any policies that show a lack of respect?*

*S2 10:13	I think it just comes down to pain management, probably, yeah, related to that. It's probably hard for them too. But I was in a car wreck last year, and I couldn't move for two months. I literally couldn't move, but they didn't give me any pain medication. So I don't know. To me, that's kind of a problem with healthcare right now. And then also, I would say, too, maybe the lack of natural remedies. Of course, they always want to prescribe you something that costs a lot, but sometimes there are natural remedies. I don't know. One time I went to the urgent care because my shoulder was hurting really bad, and she was like, "Oh, well, you just need to do this exercise," and that was kind of nice to be able to have a solution that wasn't a pill. Yeah.*

*S1 11:17	So maybe providing you with some alternatives to the normal, like you mentioned, pills and those types of medication.*

*S2 11:28	Yeah, yeah.*

Excerpt - Document: 117 Respect Interview-cleaned.docx, Position: 900-1307

*Do you have any specific examples that you’d like to share of a time when you felt respected?*

*S2 03:48	When Dr. [name] told me about my brain tumor, what was some of the side effects, what might happen, just him being straight with me of all the complication that might occur during the surgery, I think, he really showed me a lot of respect there. Just being open, putting everything out in the open. Yeah.*

Excerpt - Document: 117 Respect Interview-cleaned.docx, Position: 1677-2653

*Can you think of any examples of the sorts of things that might make you feel a lack of respect from your medical care?*

*S2 05:20	Oh, yeah. There was one time that they put a lumbar joint in my back and I was sort of telling the nurse that I’m having some really sharp back pain. And they told me not to lay down because of my lumbar joint but I keep asking for some pain medicine. And [inaudible] for them to get it to me and I think they’re not understanding my pain. So I think that that was a lack of respect. Other than that, that’s it. That’s all I have.*

*S1 06:24	Okay. Yeah. Can you say more about specifically what it was that made you feel a lack of respect from that? Sounds like they didn’t understand that you were actually in pain.*

*S2 06:36	Yeah. They didn’t understand that I’m in pain or they just don’t want to listen. I’m not sure what it is but I was to the point that I had to lay flat which caused more problem. But I could understand the pain. That was it.*

Excerpt - Document: 117 Respect Interview-cleaned.docx, Position: 3104-4001

*So you gave a good example of somewhat of a way that-- it sounds like a specific person or a specific group of people didn’t really listen and didn’t really understand your pain. Can you think of any policies that the medical institution could have that might show that they didn’t really respect you?*

*S2 08:04	No, no. Because I totally understand what was going on. Because I know they’ve been giving pain medicine but it didn’t went down and I asked for more pain medicine, but they had to check in with-- I know they have to check-in with a doctor to prove it and because they can’t give me the same pain medicine. If that pain medicine is not working, they have to put me too on a different one or a more stronger one. My answer would be no because I know what’s going on. I mean, I know the process and I know it will take a little bit longer during the process. Yeah. My answer would be no.*

Excerpt - Document: 117 Respect Interview-cleaned.docx, Position: 16140-16939

*And I’m curious with medical care, for example, you said it was a five for how much you feel respected and a three for how much you trust it. I’m just curious what you see as a difference.*

*S2 29:31	Yeah. There was one incident, not incident which my surgical doctor could not do the surgery that day so they have a substitute or a different person perform or do my surgery. And my doctor told him how to go in and replace my shunt. But the guy wanted to go from a different spot which he went in, then it was kind of hard to replace my shunt the way he went in. Then so he went back to what my original doctor told him to do to replace my shunt. And the result of that him going in, the first time he went in have my left leg numb for three years. I mean, I trust medical care but after that-- yeah.*

**Safety**: Creating a safe environment, including institutional policies that help patients feel safe or otherwise respected

Excerpt - Document: 114 Respect Interview-cleaned.docx, Position: 4512-4911

*o that’s a great example. I guess I’m trying to bring it back to that what an institution can do like maybe what kind of policies do you think would address that?*

*S2 09:42	Well, that was my idea in that there should be policies that make sure that LGBT community feels safe and that they’re able to be safe within an institution. Because if you can’t trust your medical providers, who can you trust.*

Excerpt - Document: 114 Respect Interview-cleaned.docx, Position: 11458-12639

*And sometimes people say that their experiences in the medical setting effect whether they want to join research studies, was that true for you at all?*

*S2 19:25	Actually, I’d have to yes but in a positive way. Since I’ve started going to [healthcare institution], most of my experiences have been positive. I’ve had a few times where I didn’t feel terribly listened to and few of my questions were answered as much as I would have liked them to have been. But I went into [healthcare institution]. I had to have gallbladder surgery and I had to have it removed in emergency surgery. And I went in there terrified and because how everything went with the surgery, with recovery, I felt actually extremely comfortable and I’m no longer afraid of surgery. So I think almost everyone I’ve met has had a very good to me there. And I think I like [healthcare institution] most because of the fact they have so many inclusive programs to what not?*

*S1 20:36	So between those good experiences you had and the fact that they are an inclusive institution that made you-- it sounds like those made you more interested in joining the research study. Am I getting that right?*

*S2 20:51	Yes. Yes.*

Excerpt - Document: 154 Respect Interview-cleaned.docx, Position: 5007-6312

*Yeah. Oh, I had a-- it was actually a very creepy experience. So after college, I did an internship from summer away from home, and I went to DC. And I was not-- I guess I wasn't used to the air. What it came down to was like it was kind of the pollution, but the glands in my neck swelled up like golf balls, and it hurt just to swallow. And I was freaked out because I had never had that kind of thing before. And since I had health insurance through my internship, I just picked whatever doctor was easy to get to because I didn't know the area, and I was young and scared and in a new city. Right? So I went to this doctor, and he was a older man. He was, I think, probably like Indian or Pakistani. I mean, I don't know if saying that means anything, but here's what happened. I went in for glands on my neck, right? He looked at that, said, "Oh, it's probably the pollution in the air. It'll clear up." And then he's looking at me in like this creepy way and asked me-- what do they call that cervical exam? He asked me if I had had a Pap smear recently. It's super creepy. You go in for glands in your neck, and he wants to look at your vagina? I'm like, "Ew." Yeah.*

*S1 10:53	Yeah. Completely unrelated.*

*S2 10:55	Exactly. So, yeah, that was creepy. Yeah. So that's probably enough examples from me.*

Excerpt - Document: 136 Respect Interview-cleaned.docx, Position: 2098-3167

*So what about any policies that a medical institution might have in place? Can you think of any policies that would show respect by chance?*

*S2 06:06 Oh, yes. Recently my daughter had gotten chicken pox and I wanted to make sure it was chicken pox, and I wanted to be seen by her pediatrician. And at first, one of the nurses had told me that it was not impossible to go to, to get seen, but since it's contagious that she recommended me not going in. And I had asked her if there was any way that we could still be seen? Maybe if there was a-- if there was a contaminating room that she can go into and she had to go and ask a doctor to see if that's something that could be done and eventually it was, but I had to ask her. Because at first she kind of said no. So that was, I guess, a little disrespectful.*

*S1 07:18 Okay.*

*S2 07:18 I mean, there should be a policy for that.*

*S1 07:22 Okay. So it sounds like there was a policy may be for it, but it wasn't communicated to you, is that right?*

*S2 07:32 It wasn't really communicated to the nurse because she had to ask.*

Excerpt - Document: 136 Respect Interview-cleaned.docx, Position: 4455-4873

*Or what if they give me the wrong medication or something that I'm allergic to, or my child is allergic to, that would be a really big issue. And I would be furious [laughter]. Or just being brushed off or not getting my questions answered. The would be pretty frustrating because I mean that's the main reason that I go in to get answers and to get better, and if nobody tells me anything well, then how can I, right?*

Excerpt - Document: 136 Respect Interview-cleaned.docx, Position: 6636-7747

*What about policies? We talk a little bit about policies in a positive sense. Are there any policies that a medical institution might have in place that you think shows a lack of respect? Sorry.*

*S2 04:32 I mean, we had talked about it a little bit already when I had taken my daughter for the chicken pox. I think maybe that policy needs to be stated-- or to be more clear with the staff because I was getting upset when the nurse had told me not to come in so as to not contaminate other babies and the elderly who are more prone to getting the same infections. But yeah, if I hadn't asked about maybe being secluded then she would've-- I mean, it wouldn't have happened. My daughter wouldn't have been seen. So yeah, maybe just make that policy more clear. I mean, if it is a policy because you weren't sure either, right?*

*S1 05:33 Yeah. So just communicating those policies may be, so everyone is aware?*

*S2 05:41 Yeah. Or maybe having a specific contamination room because-- I mean, everybody was just wearing extra gowns and face masks, but there wasn't any specific room that she was able to be taken into.*

Excerpt - Document: 153 Respect Interview - SP English translation - cleaned.docx, Position: 1887-2734

*Do you think you can tell me something about the providers? Something that they maybe do specifically, or maybe even the receptionist? Something that they do in particular or something that one person has done with specific action to make you feel respected?*

*S2 04:25	Yes. They always ask me before touching me or telling me something if I understand, if they can do it. My [family member] always accompanies me to the consultations and they don’t realize that he’s my [family member]. They always ask me who the person is at my side. Everyone is good, good; everyone has been very respectful and friendly towards me. I don’t have any bad experiences from anything that has happened to me, no. Even the translators, when the doctors are going to examine me, they turn their backs so as not to see where the doctors are touching me or examining me.*

Excerpt - Document: 105 Respect Interview-cleaned.docx, Position: 1412-1996

*Are there any policies that you could think of that really show respect?*

*S2 04:52	Just as far as policies, I think just sanitation and things like that nature is most important because when you're going into a hospital or a facility where thousands of people are going in and out of every day, on a daily basis, anyone can have any type of sickness. And just standard policies like they have-- like clean, sanitized, hand sanitizer all around, and hand sinks are important. If they have janitors cleaning at night and day, just like that type of stuff is really important as a policy.*

Excerpt - Document: 158 Respect Interview - SP English translation - cleaned.docx, Position: 2497-2977

*Now, what can you tell me about the policies that the medical institution has? For example, are there certain policies that show you respect? That you could think of--*

*S2 05:29	Yes. Well, being that all the clinics have their policies, such as not using the telephone, not turning it on when you arrive to register for the appointment. What else do I remember? You know, what many of them do, no smoking inside, all of that. And, for me, it seems good that it’s that way, really.*

Excerpt - Document: 146 Respect Interview- cleaned.docx, Position: 426-4844

*So with that in mind, can you give me some examples of the sorts of things that make you feel respected in your medical care?*

*S2 03:05	Well, my personal experience is the ones that actually listen to what my concerns are, not what they think my concerns are, the ones that actually take in why I'm there. And then the ones that actually take that and - how do I say this? - try to help solve the issue instead of just plopping a Band-Aid on it and saying, "Oh, it's just this. You're fine," when then I have to go back and get a second or third opinion. And the second and third opinion don't match the first opinion, and they're calling the doctor idiot or whatever.*

*S1 03:52	Okay. So could you expand on that a little bit, and I want to just ask what it is about that that makes you feel respected.*

*S2 04:02	Well, for me, personally, I've battled some ovary cyst issues the last few years. We’re not sure if it's my IUD or not, but it's caused other symptoms. So my biggest concern was I wanted my IUD out. My first and second doctor, including my OB, were like, "No. That's not it. You're just gaining weight just because you're not active," and this and that. I went and saw a new doctor, same [healthcare institution] clinic, and a new OB, and they go, "Yeah. You're not as active as you were, but your IUD is clearly playing a role in your problem." Then they asked me-- the new two doctors asked me, "Okay. Well, what are your other symptoms? What else do you have going on?" But the second set of doctors actually had the time to go through my notes, my hospital notes, everything, and not notice that, "Hey, you've had a miscarriage in the last year. Hey, you showed up at urgent care bleeding profusely with a cyst ruptured. We need to get you in now and figure out what's going on." It was those second two doctors. They're the ones that said, "We need the ultrasound immediately. We need to do the Pap smear immediately. We need to do all these tests immediately."*

*S2 05:25	Well, when I called in to-- on the ultrasound schedule, because it was put in as an emergency ultrasound, they told me it was going to be three to six weeks for me to get seen. Okay, fine. Let's get it in. We got in, I think, four weeks after the initial rupture of my cyst that we thought was a miscarriage, but luckily, that one wasn't a miscarriage. That one was an actual cyst that ruptured. It just felt like I was going into labor. The doctor called me back the next day, the new doctor. My new OB called me back the next day and said, "This is unacceptable. I cannot believe they're making you wait four weeks to get seen. We need to have you go in now." Later that afternoon, I got a phone call from the ultrasound department because there's only two on the east side that I can go to. Because of [healthcare institution], we're limited to where we can go. She was able to get me in the next day. Because of her due diligence, because of her actually listening to me and taking the time to read over my notes, she understood that this wasn't just me having cramps, and the weight gain wasn't just over a period of time. This was a rapid weight gain.*

*S2 06:38	Everything else about me was healthy, but the rapid weight gain, the fatigue, all of my symptoms were like, "No. Something else is going on. I honestly think it is your IUD. We need to have that ultrasound to figure out what's going on." If it wasn't for her, I would've never had the ultrasound done. And then still to this day, two and a half years later, still battling the cysts. So, for me, it's one of those things like-- and, of course, that doctor ended leaving on maternity leave and never came back. So now I have a new doctor that's just like my first doctor, and it's like, "Oh, well, it's not a big deal. You are in your mid-30s. You're healthy. You're overweight, but, yeah, you're healthy." I'm like, "No. There's something going on. I know my body. There's clearly something going on. It shows on the ultrasound." But the new doctor is just as bad as the first one. The second doctor was fantastic, absolutely fantastic. I'm sad she's gone. She is the first one to actually truly listen to what my concerns were. She was talking like a friend, not like a professional.*

*S1 07:53	Okay. So that's really helpful. It sounds like listening to the concerns, but also, there being a follow-up with the urgency and action and it being timely as well. Does that sound like what--?*

*S2 08:05	Yes.*

Excerpt - Document: 157 Respect Interview-cleaned.docx, Position: 1137-1435

*So would you say that communication is a big kind of factor when it comes to feeling respect?*

*S2 04:05	I think so. You maybe might have some weirder questions or something maybe that are not easier to answer-- or ask. And whenever you feel more comfortable, it might be easier to ask those things.*

Excerpt - Document: 143 Respect Interview-cleaned.docx, Position: 4230-5753

*That 45-minute wait that I talked about earlier, it wasn’t, I guess, so much the staff doing something as not doing something. Because during that time, there was another patient who decided she wanted to talk to me, and she was just saying all sorts of racist and sexist and just all sorts of things that I didn’t want to talk about, trying to talk about politics and all sorts of things when we’re waiting in the doctor’s office. And you could see that the other patients were uncomfortable including myself, and none of the staff came out and asked her to stop.*

*S1 [04:14]	Okay. So inaction on the part of the staff and maybe not maintaining kind of a-- oh, what’s the word I’m-- well, not maintaining a respectful atmosphere for everyone.*

*S2 [04:29]	Right. A trans woman came in, and she was talking so loudly about that he/she over there and like, “Urgh.” Once that person sat down, I went and moved over there. But it [inaudible].*

*[momentary loss of connection]*

*S1 [04:57]	So you had mentioned that there was this other patient that was not being very respectful of the other people that were coming into the clinic.*

*S2 [05:06]	Oh, yeah. Every person that came in, she had something negative to say about like, “Well, maybe you just--” and she kept complaining that she thought that the staff were moving her appointment farther back and back. And I wouldn’t be surprised if they had because of how rude she was [laughter], but they also didn’t do anything to stop her or help anyone else feel comfortable.*

Excerpt - Document: 117 Respect Interview-cleaned.docx, Position: 3104-4001

*So you gave a good example of somewhat of a way that-- it sounds like a specific person or a specific group of people didn’t really listen and didn’t really understand your pain. Can you think of any policies that the medical institution could have that might show that they didn’t really respect you?*

*S2 08:04	No, no. Because I totally understand what was going on. Because I know they’ve been giving pain medicine but it didn’t went down and I asked for more pain medicine, but they had to check in with-- I know they have to check-in with a doctor to prove it and because they can’t give me the same pain medicine. If that pain medicine is not working, they have to put me too on a different one or a more stronger one. My answer would be no because I know what’s going on. I mean, I know the process and I know it will take a little bit longer during the process. Yeah. My answer would be no.*

**Transparency**: Appropriately setting expectations, including admitting when medical knowledge is lacking and being upfront with patient

Excerpt - Document: 101 Respect Interview-cleaned.docx, Position: 394-1004

*So could you give me some examples of the sort of things that make you feel respected in your medical care?*

*S2 02:53	I guess just when they actually diagnose the problem right makes me feel respected. But it’s kind of one of those things where sometimes it feels like they’re guessing more than they’re actually diagnosing. Sometimes I may just feel-- when you feel like they’re guessing, you don’t really feel that much respect than them trying to take the time. But there are a lot of doctors out there-- my doctor now takes a lot of time and a lot of care in trying to figure out what’s really going on, so.*

Excerpt - Document: 101 Respect Interview-cleaned.docx, Position: 1119-2477

*What about the other medical staff? How could they show respect?*

*S2 03:50	I think nurses and them show quite a bit of respect, but just taking the time to actually talk to you and explain what they’re doing instead of just, “Sit and hold out your arm.” And actually tell you what they’re doing, if they’re taking your blood pressure or whatever, just explaining what’s going on and sharing. I’ve had nurses in the past where they don’t share what your blood pressure is or share what your heart rate is and actually take the time to tell you your whatever over whatever on your blood pressure and tell you if that’s decent, good, or something like that, so. Also, the nurses nowadays seem to be doing quite well with that, with letting you know what actually they’re doing and what they’re seeing and what they’re recording.*

*S1 04:54	Okay. So what I’m hearing is that you really appreciate kind of the ongoing dialogue and the back and forth of information and not so much them kind of just taking the information and not really filling you in.*

*S2 05:14	Right. And I mean, if they’re sharing with you, then they’re treating you like they respect you, that you’re capable of understanding what they’re talking about. When they used to keep it from you, it kind of felt like they were above you and that you wouldn’t understand what they were doing anyway, so.*

Excerpt - Document: 150 Respect Interview-cleaned.docx, Position: 3506-4191

*And then policy-wise, is there anything that you can see as having or showing a lack of respect?*

*S2 08:09	Yeah. There are some policies or guidelines that they have to do for everyone. And it felt just-- I guess I have to say an example, but I don't know how to say it. But when you get pregnant and you go into the doctor, like, "Okay, well we need to test you for all these STDs." And like, "Well, no. I'm not going to be-- why would I be tested for STDs? I'm married." And like, "Well, half of all pregnancies is not with the person they say." Like, "Well, that's not the case here." And it just felt like a very silly policy to have for someone who doesn't at all conform to that.*

Excerpt - Document: 154 Respect Interview-cleaned.docx, Position: 377-1283

*So could you give me some examples of the sorts of things that make you feel respected in your medical care?*

*S2 02:39	Well, being listened to and, yeah, eye contact. It's hard to describe. If someone's being dismissive, it's like a whole behavioral thing and not like-- it's hard to break down and pinpoint what it is that they're doing that's dismissive, but, I mean, yeah. Oh, I like it when doctors-- and I know that in-school doctors are told to use the proper terms for things, but it's frustrating when they don't want to break it down into layman's terms for you, especially if it's very clear that they don't know what's wrong. And instead of just coming out and saying, "I don't know," they're like, "Well, it could've been do, do, do, do, do, and it could've been do, do, do, do, do." Yeah, that stuff [laughter] and using big words. It's like, "Come on, come on. Just come clean with it." Sorry.*

Excerpt - Document: 154 Respect Interview-cleaned.docx, Position: 2573-3194

*So I was curious if you could kind of give me an idea of the sorts of things that make you feel a lack of respect.*

*S2 05:38	I feel like I kind of just laid that out. It's kind of the same thing, I mean, regardless of who it's coming from, dismissive behavior, maybe not making eye contact. Oh, kind of passing the bucks and, "It's not our responsibility. It's somebody else's responsibility," that's frustrating. That's something I've dealt with before. Yeah. I mean, even if it's not your responsibility to do something or find the answer to something, it'd be nice to say, "Hey, let me get you somebody who can." Right?*

Excerpt - Document: 172 Respect Interview - SP English translation - cleaned.docx, Position: 1388-1660

*in your medical care, is there a specific moment you can think of when you felt respected?*

*S2 03:23	In their communication when they’re going to check you out, explaining to you everything they’re doing or what they are going to do with you at the moment they check you.*

Excerpt - Document: 136 Respect Interview-cleaned.docx, Position: 6636-7747

*What about policies? We talk a little bit about policies in a positive sense. Are there any policies that a medical institution might have in place that you think shows a lack of respect? Sorry.*

*S2 04:32 I mean, we had talked about it a little bit already when I had taken my daughter for the chicken pox. I think maybe that policy needs to be stated-- or to be more clear with the staff because I was getting upset when the nurse had told me not to come in so as to not contaminate other babies and the elderly who are more prone to getting the same infections. But yeah, if I hadn't asked about maybe being secluded then she would've-- I mean, it wouldn't have happened. My daughter wouldn't have been seen. So yeah, maybe just make that policy more clear. I mean, if it is a policy because you weren't sure either, right?*

*S1 05:33 Yeah. So just communicating those policies may be, so everyone is aware?*

*S2 05:41 Yeah. Or maybe having a specific contamination room because-- I mean, everybody was just wearing extra gowns and face masks, but there wasn't any specific room that she was able to be taken into.*

Excerpt - Document: 153 Respect Interview - SP English translation - cleaned.docx, Position: 1887-2734

*Do you think you can tell me something about the providers? Something that they maybe do specifically, or maybe even the receptionist? Something that they do in particular or something that one person has done with specific action to make you feel respected?*

*S2 04:25	Yes. They always ask me before touching me or telling me something if I understand, if they can do it. My [family member] always accompanies me to the consultations and they don’t realize that he’s my [family member]. They always ask me who the person is at my side. Everyone is good, good; everyone has been very respectful and friendly towards me. I don’t have any bad experiences from anything that has happened to me, no. Even the translators, when the doctors are going to examine me, they turn their backs so as not to see where the doctors are touching me or examining me.*

Excerpt - Document: 123 Respect Interview-cleaned.docx, Position: 2212-4099

*And I guess I should ask can you think of any particular examples when you’ve really felt like you’re being really well respected?*

*S2 04:53	Yeah. So I was not well very much. I’ve had chronic stomach problems for over 20 years and I’m overweight but I’m not significantly overweight. I’m not like morbidly overweight. And so throughout the years, I got a new complaint with the stomach illness and I’ve had all sorts of weird experiences where people have drawn mice and some mice are skinny and some are fat. Last time, I was compared to fat donut which was really a weird thing. But the positive experience is that I was going to have maybe a stomach repair surgery and I went to see the surgeon. And he actually listened to all of my complaints and said, “Oh, okay. That makes sense. You’re a little overweight but you’re not that overweight and your symptomology doesn’t mean what you’re saying and then if I do this surgery without really checking into it, it can cause you a lot of harm.” He explained all the procedure to me, what that could look like and then he said I’m going to do some extra testing because I want to make sure. And then, knowing the cause because of the test and he referred me to-- they actually found the stomach illness that I’ve probably have had over 20 years, and it was because he actually listened to me and it wasn’t just like default, “Oh, you have to shed your pounds. This is severe.” Or this is like, “Hey, this doesn’t kind of match up.” I’m like, “Yeah. That’s it. That’s what I’ve been saying.” And so just that-- so his name was Dr. [name]. His attention to detail and really like, “What?” where he was just like curious about it too. I mean, it’s been a little bit of life-changing for me.*

*S1 06:33	Yeah. So it sounds like it wasn’t just a matter of showing respect but it actually had a clinical impact on your care.*

*S2 06:42	Yeah. It did.*

Excerpt - Document: 123 Respect Interview-cleaned.docx, Position: 4119-8710

*you sort of alluded to this but can you give some examples of times when you felt a lack of respect in medical care?*

*S2 06:52	Yeah. So on my journey with stomach stuff, when I was going in-- I mean, I was throwing up in my sleep and so I went into the examination room. So we don’t know if they’re primary care and the woman I saw, I said, “I’m throwing up almost twice a week in sleeping mode.” And she’s like, “Well, you only have two episodes. That’s good.” I’m like, “I don’t think that’s true.” You know, I get it. I’m a [occupation], I’m not a doctor but that doesn’t make sense because you can die when you’re hearing that phrase, that’s disrespect. And so she was just like, “No. Two episodes is good.” And I asked to talk to a specialist and she said I didn’t need to see a specialist. So she really didn’t validate my feelings nor did she wish my medical desire which I felt was ridiculous. And then, I went back. A week later, once again I was throwing up in my sleep. I went in and saw-- I don’t know if he was a nurse practitioner or something, and he was just like, “What? Some people are thin donuts and some people are fat donuts.” And he actually compared me to like a glazed, sprinkled Mustang donut and was saying that my digestive system is like the center of that donut and if I were just as more compact donut that was more fit, my food would just go through me. But since I was more spread out like that fat sprinkled donut, then I was going to continue to have problems, and I was just more shattered. Then, I said, “You know, as I’ve gone through this journey and I extraordinarily lost a bunch of weight and I’m still working out.” And then I got shin splint. I tore my rotator cuff. I’ve had a series of cortisone shots and all of these things, and I’m kind of afraid to jump back. And your family is like, “Well, a cortisone shot is better than not being fat.” And I think it could probably be two ways.*

*S2 08:50	So again, he was just so quick to jump on the weight bandwagon. He wasn’t listening to my symptomology at all, and he was just like, “Well, you know.” And then, this was like minimize shame and it seemed ridiculous to me also like is that really the best solution our medical providers can give us. It’s like, “Well, you’re obviously in some pain. Just go get another cortisone shot” which is weird because there’s research about cortisone shots that you’re only supposed to have so many and he was just dismissive of it. But then I had a really bad example where I had had a bad pedicure but not a chronic issue. But I had a bad pedicure and I had gotten an ingrown toenail. So I went in and I was telling the provider. They’re like, “Oh, just soak it.” So I did and it didn’t resolve. And so it ended up being where I had to have it removed. And I don’t what he was like a physician’s assistant or something. He had a student come in and said, “Do you mind the assistance?” I’m like, “You know, I really kind of prefer, no offense to you.” Whilst she’s trying to learn and now what has happened is-- so I felt pressured. He said, “She’s a very nice old student.” So I’m like, “Okay. She can assist.” They didn’t know my foot. My foot ended up being-- I don’t know. Well, at least 12 shots in my toe, so the toe ended up being bruised for a week. They removed half of my toenail. And because students used like a little bottlebrush with the root. They killed the toenail root. My toenail now can only grow about a quarter inch out and then it dies because they’ve permanently damaged my root. So I don’t know. Well, it wasn’t respectful. I didn’t want the student. They probably shouldn’t ask if a student is right there because it puts the patient in a precarious spot. When I said no, he didn’t accept no, “Oh, common. She’s just trying to learn; I’ll be here.” And then, she failed with that and now I have a permanent like-- does it kill me? No.*

*S2 10:52	But I’ve had to have that toenail removed a couple of times since then. I never had ingrown toenails before. It wasn’t like I had a chronic problem where I needed that level of treatment. And now, it literally dies off and it turns like almost a bluish color. It’s unsightly that doesn’t kill me but had he just listened to me in the first place perhaps I wouldn’t have this and I actually asked to see a specialist for it. And my providers said I didn’t need to.*

*S1 11:25	Yikes. Well, I’m sorry to hear you have so many examples of-- that sounds rough so I’m sorry you had to go through that.*

*S2 11:35	Like if I’m old, over 45 years, this wouldn’t have been bad, but I’m [in my early 30s].*

Excerpt - Document: 123 Respect Interview-cleaned.docx, Position: 9072-9894

*I’m going to ask the same question as before, which is are there any policies that you could think of that might show a lack of respect from an institutional level?*

*S2 12:17	No. Well, I don’t know if they have the policy but I-- first of all, [healthcare institution] is not necessarily a teaching hospital. But I understand they provide the interns their practicums from a university. So I honor and respect that but I think that at a point, you should probably ask and I don’t know if they have policies about it. So maybe they should implement those where they ask the patients privately without the student being present so we’re not put in a precarious spot. And then, if there’s any hesitancy, you can explain that or you can just accept no and move on. I’m sure there’s plenty of patients that would gladly consent.*

Excerpt - Document: 142 Respect Interview- cleaned.docx, Position: 1230-2067

*Can you tell me what it is-- one thing I want to kind of focus on is that you mentioned the one-on-one time, the listening, the eye-contact. And that all kind of reminds me of the same thing. Can you expand on those things? What is it about that, that makes you feel respected, being listened to?*

*S2 04:25	I guess, I just shows that they care and not making me feel like I'm just one of many appointments that they have to get through. But that they actually want to put time and effort into patient care and in my health. The body language says a lot too. If they are facing towards me and, like I said, making that eye contact, makes it seem like they're trying to be more understanding and relatable in that sense, in that whatever I'm going through is. Okay, maybe relaying information the way that I would be able to understand it.*

Excerpt - Document: 129 Respect Interview-cleaned.docx, Position: 413-958

*So could you tell me or-- sorry, could give me some examples of the sort of things that make you feel respected in your medical care?*

*S2 02:47	Well, like the appointments that I had even when I was pregnant or before, I felt like the doctors were really listening to my concerns, and also trying to explain how things would go and all the things that could be helpful for me to know without overwhelming my brain with too much information.*

*S1 03:14	Okay. All right. So for you, it’s a lot about the communication aspect of it.*

*S2 03:22	Yes, yes.*

Excerpt - Document: 129 Respect Interview-cleaned.docx, Position: 2302-3652

*So with this one, can you give me some examples of the sorts of things that might make you feel a lack of respect with your medical care?*

*S2 05:36	Well, I do feel and maybe I’m-- I mean, it’s just the way I feel. And I’m sorry to just kind of bring back again my pregnancy and stuff but that’s been my biggest experience with the hospital and medical care in the state. So I would have to say that I kind of felt a little pressure to have an induced delivery which I was not really-- which I did not want. So I did feel like they were kind of pressuring me too much and at the moment, I was-- well, of course, it was the first time for me having a baby so I was not really thinking and I just felt I got too much pressure into telling me, “You have to do this and you cannot leave,” and that kind of thing. So that was the only thing that I was not very happy with anybody. I just wanted like a little bit of time and maybe like some more time to talk to doctors and that they will kind of sit and tell me more like consequences and all those things.*

*S1 06:59	Okay. So feeling a little bit of pressure and not having the opportunity to kind of start that line of dialogue or the communication that we spoke about earlier.*

*S2 07:10	Yes. I mean, before that I had like-- I don’t really have any complaints but just like for my delivery, I do have some.*

Excerpt - Document: 156 Respect Interview-cleaned.docx, Position: 2856-3619

*And so I want to ask you if you could give me some examples of the sorts of things that might make you feel a lack of respect in your medical care.*

*S2 07:04	I feel like with doctors, it's almost like they think they're better, the ones that I've hand anyways. There's been a few that just act like they know for sure when there's not really necessarily a certain answer for something, so I don't like that. And then not fully being aware of somebody's medical history--*

*S1 07:49	On the doctor's end?*

*S2 07:52	Yeah. That they don't double-check a chart to see if everything's the same before they actually talk to you or something. But I think the biggest one is just not talking to the patient like they're an actual person. It's almost like being talked down to.*

Excerpt - Document: 174 Respect Interview - SP English translation - cleaned.docx, Position: 907-1738

*I’d like to step back and ask you to think about your experiences with medical care, including the doctors and clinics that you visit. So, could you give me some examples of the things that make you feel respected in your medical care?*

*S2 03:00	Well, they attend to me, they explain the problem well, about what I'm going through. They always treat me with respect, I mean, they always resolve my doubts about what I have. I mean, if I have any questions, they always provide me with a good answer.*

*S1 03:30	Okay. And, was there ever a moment when you felt respect-- sorry you already told me that with your answer. But, what can you tell me about the providers or the doctors? Have they done something different than-- even at the clinic or would you say that everyone who works at the clinic acts the same?*

*S2 03:57	Everyone.*

Excerpt - Document: 116 Respect Interview-cleaned.docx, Position: 433-990

*So could you give me some examples of the sorts of things that make you feel respected in your medical care?*

*S2 02:58	I think, so far - knock on wood - I haven’t had to get much medical care lately. But when I have, I like-- I’m trying to think here. I guess the big things for me would be consideration of privacy and their timeliness on how they get back to me about results on stuff if I have any tests done or whatnot. And just giving me as much information as possible about any questions I may ask about medical issues that I have, that sort of thing.*

Excerpt - Document: 113 Respect Interview_cleaned.docx, Position: 398-1146

*So could you give me some examples of the sorts of things that make you feel respected in your medical care?*

*S2 02:46	Well, I recently had a stroke, so I've been going to see a doctor often. I like it when they're truthful and honest and when they don't beat around the bush. I feel more respected when people are just honest and look you straight in the eye and tell you what the conditions or what their concerns or what you need to do get better. Instead of going around the bush and saying, "Well, maybe you could try this," or, "Maybe if you try that." I'd rather just be frank and honest and I find that more respectful than beating around the bush. But I haven't had a bad experience. I do find [the healthcare system to be] very respectful.*

Excerpt - Document: 148 Respect Interview - SP English Translation-cleaned.docx, Position: 939-1477

*Could you give me some examples of the things that make you feel respected by your medical care?*

*S2 02:45	By medical care?*

*S1 02:46	Yes.*

*S2 02:48	The way that the assistants as well as the doctors treat me is always respectful.*

*S1 02:57	Okay. And do you have examples of things that the nurses or doctors at the clinic have done?*

*S2 03:14	For example, at any checkup, before anything else, they first ask me if it’s okay for them to perform whatever procedures may be necessary. They don’t do anything without asking for my opinion first.*

Excerpt - Document: 133 Respect Interview-cleaned.docx, Position: 4624-5731

*Now I’d like to kind of go on the opposite side of that and see if there are any examples that you could give me of things that make you feel a lack of respect in your medical care?*

*S2 06:03	Yeah. Again, I don’t have experiences, like I said, I’m generally very healthy. From what I had to deal with [inaudible], for example, I had surgery a while back on my hand. I don’t think I felt disrespected but my doctor didn’t-- I feel like he [inaudible] some portion of it, telling me about the downtime after the procedure, of the surgery, how long I would be down for. I feel like I didn’t get a true understanding of how long I was going to be truly down and not be able to use my hand for the activities that I do daily. So in that sense, I wouldn’t say I feel disrespected or I felt a lack of respect there, but I did feel that they didn’t provide me with enough information to say, “Okay, this is what I want to do.” Or try and get an idea of what I’ll be facing later on. So in that aspect, it’s a little bit of it-- I feel, there was a little bit lack of respect there for my job and my daily activities.*

Excerpt - Document: 168 Respect Interview - SP English translation - cleaned.docx, Position: 1574-1962

*Could you give me some examples of things that make you feel respected during your medical care?*

*S2 03:08	[inaudible](Sorry), some examples of what? Sorry.*

*S1 03:13	How you feel respected when you go, for example, to the clinic to--*

*S2 03:19	Okay. For example when they give me information or ask me if I have any questions, and they’re attentive to how, exactly, I feel at that moment.*

Excerpt - Document: 143 Respect Interview-cleaned.docx, Position: 1445-2516

*So maybe beyond thinking of just the doctors themselves, when you, say, interact with maybe the intake staff or the nurses, is there anything there that kind of jumps out at you?*

*S2 [04:23]	If they hear one of my concerns and then ask a follow-up question, it feels like they’re really listening and trying to figure it out rather than just checking me in.*

*S1 [04:34]	All right. Questions and concerns.*

*S2 [04:41]	When they explain not just what it is that’s causing the problem but what could be done to [inaudible].*

*[call disconnected]*

*S1 [00:01]	All right. I’ve restarted the recording. And sorry about that. So let’s--*

*S2 [00:04]	All right. No worries.*

*S1 [00:07]	All right. So the last thing I was able to hear was that when you had questions, the staff had some follow up-- or there were follow-up questions, excuse me, to your concerns that you raised.*

*S2 [00:21]	Yes. Right. And then the next thing I was saying was that when I explained whatever my symptoms are, beyond just telling me what the problem is, when they explain what may have caused that.*

Excerpt - Document: 131 Respect Interview-cleaned.docx, Position: 3072-5095

*So for this next question, I’d like to kind of flip the previous question on its head and ask you to give me some examples of the sorts of things that might make you feel a lack of respect in medical care.*

*S2 07:16	Okay.*

*S1 07:17	So I imagine that a lot of it could be just kind of the reverse of what we had talked about before with communication, and confidentiality, and privacy. But if there’s anything else that stands out to you or maybe any experiences that you’ve had in the past--*

*S2 07:36	Sure. Well, yeah, like the thing I mentioned before, where random self issues come up and the doctors blames weight management for the problem, rather than listening to the problem. I would say that that is something that not only has applied to me in the past, but people I know, where they don’t even want to go to the doctor. Because they don’t want to get lectured about their weight, or their diabetes, or whatever disability they have that they feel like the doctor blames every health problem on the one thing rather than looking at the person as a whole. But not everything is all about their diagnoses. And yeah, just the opposites of what we talked about a moment ago, like just feeling lot listened to, or dismissed, or lectured, rather than sharing information and feeling like you’re supported.*

*S1 08:45	Okay.*

*S2 08:49	And I’m also a person who likes to ask a lot of questions. So I appreciate it when a doctor is honest with me, like, “Well, we don’t know why it’s this way. But what we’re going to do to try to find out is this, this, and this.” Or explaining what the diagnosis means, rather than just saying, “Oh, it’s this thing. Here’s your prescription. Call me if it doesn’t get better.”*

*S1 09:13	Okay. And so I think those are interesting. Because there are also aspects of that communication element that we talked about with honesty being kind of an open line of communication. Again, you just mentioned being given information instead of handed a prescription and sent out the door.*

*S2 09:44	Right.*

Excerpt - Document: 117 Respect Interview-cleaned.docx, Position: 900-1307

*Do you have any specific examples that you’d like to share of a time when you felt respected?*

*S2 03:48	When Dr. [name] told me about my brain tumor, what was some of the side effects, what might happen, just him being straight with me of all the complication that might occur during the surgery, I think, he really showed me a lot of respect there. Just being open, putting everything out in the open. Yeah.*

Excerpt - Document: 145 Respect Interview-cleaned.docx, Position: 2110-3095

*So now, I want to kind of flip all those questions on their head and ask you if you could give me some examples of sorts of things that might make you feel a lack of respect from your medical care?*

*S2 05:15	Not following up in a timely manner if you're waiting for any type of results. I don't know if it's disrespectful, but I think it goes back to what I was saying before. Just making sure that someone gives you time and then again, that feeling where actually makes me feel like my issue is not necessarily important. And not having a professional demeanor. I mean, I know we all have bad days, but sometimes people get snappy and healthcare is a stressful job. Let's be honest. So I get it to some degree but not taking that out on your patients.*

*S1 06:09	Okay.*

*S2 06:19	Let's see. And I think if you don't get a full picture of what medical costs are going to be. So I guess not being thorough and transparent and sort of fees-- or again what's happening and why it's happening.*
